# Morphoelasticity of large bending deformations of cell sheets during development

**DOI:** 10.1103/PhysRevE.103.022411

**Published:** 2021-02-01

**Authors:** Pierre A. Haas, Raymond E. Goldstein

**Affiliations:** 1 Department of Applied Mathematics and Theoretical Physics, Centre for Mathematical Sciences, University of Cambridge, Wilberforce Road, Cambridge CB3 0WA, United Kingdom; 2 Mathematical Institute, University of Oxford, Woodstock Road, Oxford OX2 6GG, United Kingdom

## Abstract

Deformations of cell sheets during morphogenesis are driven by developmental processes such as cell division and cell shape changes. In morphoelastic shell theories of development, these processes appear as variations of the intrinsic geometry of a thin elastic shell. However, morphogenesis often involves large bending deformations that are outside the formal range of validity of these shell theories. Here, by asymptotic expansion of three-dimensional incompressible morphoelasticity in the limit of a thin shell, we derive a shell theory for large intrinsic bending deformations and emphasize the resulting geometric material anisotropy and the elastic role of cell constriction. Taking the invagination of the green alga *Volvox* as a model developmental event, we show how results for this theory differ from those for a classical shell theory that is not formally valid for these large bending deformations and reveal how these geometric effects stabilize invagination.

## I Introduction

Cell division, cell shape changes, and related processes can drive deformations of cell sheets during animal and plant development [1–6]. In elastic continuum theories of the development of the green alga *Volvox* [7–10], of tissue folding in *Drosophila* [11,12], or of more abstract active surfaces [13], these driving processes appear as changes of the reference or intrinsic geometry of thin elastic shells.

Just as classical thin shell theories arise from an asymptotic expansion of bulk elasticity in the small thickness of the shell [14–16], these morphoelastic shell theories should be asymptotic limits of a bulk theory. While there is now a well-established framework of three-dimensional morphoelasticity [17,18], based on a multiplicative decomposition of the deformation gradient tensor into intrinsic and elastic deformations [19], studies of this asymptotic limit have mostly been restricted to the case of flat morphoelastic plates. Extensions of the classical Föppl–von Kármán equations [20,21] have been derived and residual stresses in Kirchhoff plate theories [22] have been studied in this case. A theory of non-Euclidean plates [23] has been developed in parallel. Apart from a general geometric theory of morphoelastic surfaces [24], studies of morphoelastic shells have remained more phenomenological, however: Some models [7,8,11–13] simply replaced the elastic strains in classical shell theories [15,25,26] with measures of the difference of the intrinsic and deformed geometries. Other studies [9,10] took a more geometric approach, mirroring geometric derivations of classical shell theories [25] based on the so-called Kirchhoff “hypothesis”. This is the asymptotic result [15] that the normals of the midsurface of the undeformed shell remain, at leading order, normal to the deformed midsurface.

**Fig. 1:**
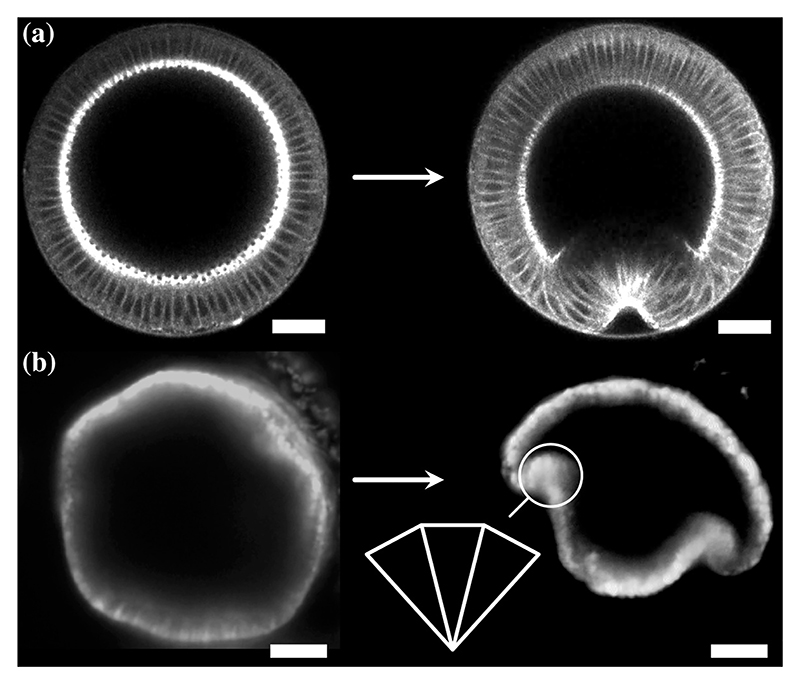
Large bending deformations during morphogenesis: Even if the thickness of the cell sheet is small compared to the undeformed radius of curvature, the local radius of curvature need not remain large compared to the cell sheet thickness as the sheet deforms. (a) Cross section of ventral furrow formation in *Drosophila*, reproduced from Ref. [29]. (b) Midsagittal cross section of invagination in the spherical alga *Volvox globator*, reproduced from Ref. [8]. Inset: Cartoon of constricted triangular cells in the bend region. Scale bars: 20 *μ*m.

There is, however, one more serious limitation of these models: Tissues in development undergo large bending deformations (Fig. 1) that are outside the formal range of validity of the underlying thin shell theories, which assume that the thickness of the shell is much smaller than all length scales of the midsurface of the shell [15,25,26]. However, even if the thickness of the cell sheet is much smaller than its undeformed radius of curvature, this radius of curvature may become comparable, locally, to the thickness of the cell sheet as it deforms (Fig. 1). This is associated with cells contracting at one cell pole to splay and thereby bend the cell sheet [4].

Here, we derive a theory of thin incompressible morphoelastic shells undergoing large bending deformations by asymptotic expansion of three-dimensional elasticity. We reveal how, even in a constitutively isotropic material, this biological scaling limit of large bending deformations induces, in the thin shell limit, a geometric anisotropy absent from classical shell theories: different deformation directions exhibit different deformation responses. We stress how this geometric effect is associated with the geometric singularity of cell constriction, i.e., the limit of wedged triangular cells [Fig. 1(b), inset] associated with these large bending deformations. Specializing to the invagination of the green alga *Volvox* [27,28], we then show how results for this theory differ from those for a classical theory that is not formally valid in this large bending limit and reveal how invagination is stabilized by the geometry of large bending deformations.

## II Elastic Model

**Fig. 2:**
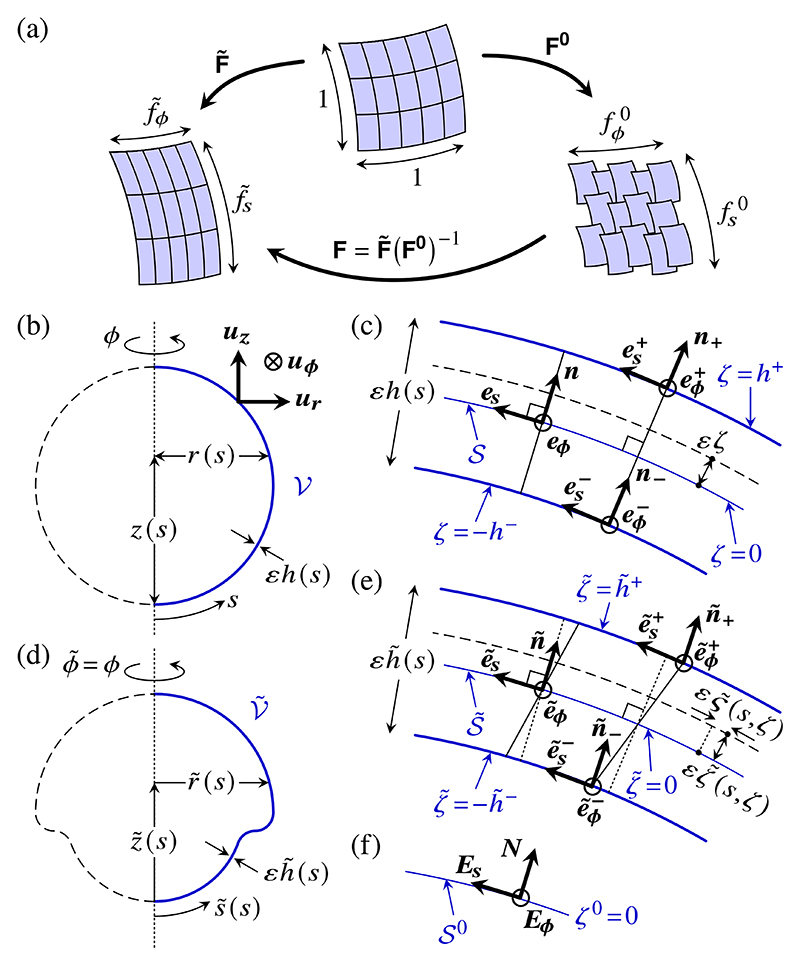
Morphoelasticity of an axisymmetric shell. (a) The undeformed (top), deformed (left), and intrinsic (right) configurations of the shell are related by the three tensors $\tilde{F},F^{0},$ and $F=\tilde{F}{\left(F^{0}\right)}^{-1}$. The geometric and intrinsic midsurface stretches are $\tilde{f_{s}},{\tilde{f}}_{\phi }$ and $f_{s}^{0},f_{\phi }^{0}$. (b) Undeformed configuration *V* of an axisymmetric shell of thickness *εh*(*s*), described by coordinates *r*(*s*), *z*(*s*), where *s* is arclength, with respect to the basis {***u***_***r***_, ***u***_***ϕ***_, ***u***_***z***_} of cylindrical polars. (c) Cross section of the undeformed shell, defining a basis *ℬ*= {***e***_***s***_, ***e***_***ϕ***_, ***n***} and the transverse coordinate *ζ*. The surfaces of the undeformed shell are at *ζ* = ±*h*^±^(*s*), where the tangent vectors are $e_{s}^{\pm },e_{\phi }^{\pm }$, and the normal is ***n***^**±**^. (d) Deformed configuration $\tilde{V}$ of the shell: After a torsionless deformation, the shell has thickness $\varepsilon \tilde{h}(s)$, arclength $\tilde{s}$, and is described by coordinates $\tilde{r}(s),\tilde{z}(s)$ with respect to cylindrical polars. (e) Cross section of the deformed shell, defining a basis $\tilde{ℬ}=\left\{{\tilde{e}}_{s},{\tilde{e}}_{\phi },\tilde{n}\right\}$. Normals to the midsurface rotate so that a point at a distance *εζ* from the undeformed midsurface *𝒮* is at a distance $\varepsilon \tilde{\zeta }(s,\zeta )$ from the deformed midsurface $\tilde{S}$ and displaced by a distance $\varepsilon \tilde{\zeta }(s,\zeta )$ parallel to $\tilde{S}$. At the surfaces $\tilde{\zeta }=\pm {\tilde{h}}^{\pm }(s)$ of the deformed shell, the tangent vectors are ${\tilde{e}}_{s}^{\pm },{\tilde{e}}_{\phi }^{\pm }$, and the normal is ***ñ***^**±**^. (f) The intrinsic midsurface *S*^0^, on which *ζ* ^0^ = 0, embeds, locally, into three-dimensional space to define an intrinsic basis *ℬ*^0^ = {***E***_***s***_, ***E***_***ϕ***_, ***N***}.

In this section, we describe large bending deformations of a thin incompressible morphoelastic shell, starting from three-dimensional morphoelasticity. We shall have to distinguish between three configurations of the shell [Fig. 2(a)]: (i) the undeformed configuration of the shell, (ii) the deformed configuration of the shell, and (iii) the intrinsic configuration of the shell that encodes the local, intrinsic deformations of the shell, i.e., the cell shape changes or cell division in the biological system. These intrinsic deformations are not in general compatible with the global geometry of the shell: in other words, this intrinsic configuration cannot, in general, be embedded into three-dimensional Euclidean space [17]. Elasticity must therefore intervene to “glue” the intrinsically deformed infinitesimal patches of cell sheet back together, as illustrated in Fig. 2(a). Configurations (i) and (ii) are related by the geometric deformation gradient $\tilde{F}$. This tensor decomposes multiplicatively into an intrinsic contribution **F**^**0**^ that relates configurations (i) and (iii), and an elastic contribution $F=\tilde{F}{\left(F^{0}\right)}^{-1}$. This is the multiplicative decomposition of morphoelasticity [17,18].

In this section, we restrict to torsionless deformations of an axisymmetric shell. The analysis can be extended to more general deformations of the shell, and, for the sake of completeness, we do so in Appendix A, but the restriction to axisymmetric deformations eschews the mire of tensorial notation that arises in the general case.

The derivation of the shell theory for large bending deformations divides, like derivations of classical shell theories, into two steps: First, in Sec. II A, we describe the kinematics of the deformation and derive expressions for the geometric, intrinsic, and elastic deformations gradients. Second, in Sec. II B, we analyze the mechanics of the shell and expand the three-dimensional elastic energy and equilibrium conditions asymptotically. At the end of this section, in Sec. II C, we discuss the limit of small bending deformations that gives rise to classical shell theories.

### A Axisymmetric deformations of an elastic shell

We consider an elastic shell of undeformed thickness *εh*, where *ε* ≪ 1 is a small asymptotic parameter expressing the thinness of the shell compared to other length scales associated with its midsurface. Large bending deformations will be introduced in Sec. II B by allowing one of the intrinsic radii of curvature of the shell to be of order *O*(*ε*). We begin by deriving an expression for the elastic deformation gradient **F** for torsionless deformations of an axisymmetric shell.

#### 1 Undeformed configuration of the shell

We will describe the undeformed configuration *𝒱* of the shell with reference to a midsurface *S* that we will choose later. With respect to the basis {***u***_***r***_, ***u***_***ϕ***_, ***u***_***z***_} of cylindrical coordinates, we define the position vector of a point on *𝒮*,

(1) $$\rho (s,\phi )=r(s)u_{r}(\phi )+z(s)u_{z},$$

with *s* denoting arclength and *ϕ* being the azimuthal coordinate [Fig. 2(b)]. The tangent angle *ψ* (*s*) of *𝒮* is defined by

(2) $$r^{\prime }(s)=\cos\psi (s),\;z^{\prime }(s)=\sin\psi (s),$$

in which dashes denote differentiation with respect to *s*. The vectors

(3) $$e_{s}(s,\phi )=\cos\psi (s)u_{r}(\phi )+\sin\psi (s)u_{z},\;e_{\phi }(\phi )=u_{\phi }(\phi )$$

thus constitute a basis of the tangent space of *𝒮* [Fig. 2(c)], which we extend to a (right-handed) orthonormal basis *ℬ* = {***e***_***s***_, ***e***_***ϕ***_, ***n***} for *𝒱* by adjoining the normal to *𝒮*,

(4) $$n(s,\phi )=\cos\psi (s)u_{z}-\sin\psi (s)u_{r}(\phi ).$$

In particular, ***n*** = ***e***_***s***_ × ***e***_***ϕ***_. We complete the description of *𝒮* by computing its curvatures,

(5) $$\varkappa_{s}(s)=\psi^{\prime }(s),\;\varkappa_{\phi }(s)=\frac{\sin\psi (s)}{r(s)}.$$

Now, the position of a point in *𝒱* is

(6) r(s,ϕ,ζ)=ρ(s,ϕ)+εζn(s,ϕ),

where we have introduced the transverse coordinate *ζ*, which is such that the shell surfaces are at *ζ* = ±*h*^±^(*s*) [Fig. 2(c)]. Noting the derivatives ∂***n****/*∂*s* = −ϰ_*s*_***e***_***s***_ and ∂***n****/*∂*ϕ* = −ϰ_*ϕ*_***e***_***ϕ***_, we obtain the tangent basis of *𝒱*,

(7) $$\frac{\partial r}{\partial s}=\left(1-\varepsilon \varkappa_{s}\zeta \right)e_{s},\;\frac{\partial r}{\partial \phi }=r\left(1-\varepsilon \varkappa_{\phi }\zeta \right)e_{\phi },\;\frac{\partial r}{\partial \zeta }=\varepsilon n,$$

from which follows the expression for the Riemannian metric of the undeformed configuration,

(8a) $$\chi_{s}^{2}\;\text{d}s^{2}+\chi_{\phi }^{2}\;\text{d}\phi^{2}+\chi_{\zeta }^{2}\;\text{d}\zeta^{2},$$

with associated scale factors

(8b) $$\chi_{s}=1-\varepsilon \varkappa_{s}\zeta ,\;\chi_{\phi }=r\;(1-\varepsilon \varkappa_{\phi }\zeta ),\;\chi_{\zeta }=\varepsilon ,$$

and hence volume element

(8c) $$\begin{array}{l} \text{d}V=\chi_{s}\chi_{\phi }\chi_{\zeta }\text{d}s\;\text{d}\phi \;\text{d}\zeta \\ \;=\varepsilon (1-\varepsilon \varkappa_{s}\zeta )(1-\varepsilon \varkappa_{\phi }\zeta )r\;\text{d}s\;\text{d}\phi \;\text{d}\zeta . \end{array}$$

The position vectors of the surfaces *ζ* = ±*h*^±^(*s*) of the undeformed shell are

(9a) $$r^{\pm }(s,\phi ,\zeta )=\rho (s,\phi )\pm \varepsilon h^{\pm }(s)\;n(s,\phi )$$

so, using commata to denote partial differentiation,

(9b) $$\frac{\partial r^{\pm }}{\partial s}=\left(1\mp \varepsilon \varkappa_{s}h^{\pm }\right)e_{s}\pm \varepsilon h_{,s}^{\pm }n.$$

The unit tangent vectors to the shell surfaces are $e_{s}^{\pm }∥\partial r^{\pm }/\partial s$ and $e_{\phi }^{\pm }=e_{\phi }$, in which the symbol || expresses parallelism and hides a normalization factor for the unit vector on the left-hand side. By definition, the unit normals ***n***^**±**^ to the undeformed shell surfaces [Fig. 2(c)] obey $n^{\pm }∥e_{s}^{\pm }\times e_{\phi }^{\pm }$. Now introducing the normalization factor explicitly, we find

(10) $$n^{\pm }=\frac{n\mp v_{\pm }e_{s}}{\sqrt{1+v_{\pm }^{2}}}\;\;\text{with}\;\;v_{\pm }=\frac{\varepsilon h_{,s}^{\pm }}{1\mp \varepsilon \varkappa_{s}h^{\pm }}.$$

#### 2 Deformed configuration of the shell

As the shell deforms into its deformed configuration $\tilde{V}$, the midsurface *𝒮* maps to the deformed midsurface $\tilde{S}$ [Fig. 2(d)], with position vector

(11) $$\tilde{\rho }(s,\phi )=\tilde{r}(s)u_{r}(\phi )+\tilde{z}(s)u_{z},$$

where, in particular, *s* is again the undeformed arclength. Denoting by $\tilde{s}$ the deformed arclength, we define the stretches

(12) $${\tilde{f}}_{s}(s)=\frac{\text{d}\tilde{s}}{\text{d}s},\;{\tilde{f}}_{\phi }(s)=\frac{\tilde{r}(s)}{r(s)},$$

which enable us to define the tangent angle $\tilde{\psi }(s)$ of $\tilde{S}$ by

(13) $${\tilde{r}}^{\prime }(s)={\tilde{f}}_{s}\cos\tilde{\psi }(s),\;{\tilde{z}}^{\prime }(s)={\tilde{f}}_{s}\sin\tilde{\psi }(s),$$

where dashes still denote differentiation with respect to *s*. Similarly to the analysis of the undeformed configuration, we introduce the tangent vectors

(14) $${\tilde{e}}_{s}(s,\phi )=\cos\tilde{\psi }(s)u_{r}(\phi )+\sin\tilde{\psi }(s)u_{z},\;{\tilde{\;e}}_{\phi }(\phi )=u_{\phi }(\phi ),$$

and the normal vector

(15) $$\tilde{n}(s,\phi )=\cos\tilde{\psi }(s)u_{z}-\sin\tilde{\psi }(s)u_{r}(\phi ),$$

so $\tilde{n}={\tilde{e}}_{s}\times {\tilde{e}}_{\phi }$. This defines a (right-handed) orthonormal basis $\tilde{ℬ}=\left\{{\tilde{e}}_{s},{\tilde{e}}_{\phi },\tilde{n}\right\}$ describing $\tilde{V}$ [Fig. 2(e)]. The curvatures of the deformed shell are

(16) $${\tilde{\kappa }}_{s}(s)=\frac{{\tilde{\psi }}^{\prime }(s)}{{\tilde{f}}_{s}(s)},\;{\tilde{\kappa }}_{\phi }(s)=\frac{\sin\tilde{\psi }(s)}{\tilde{r}(s)}.$$

As the shell deforms, the normals to *𝒮* need not remain normal to $\tilde{S}$, and so a point in *𝒱* at a distance *εζ* from *𝒮* will end up, in $\tilde{V}$, at a distance $\varepsilon \tilde{\zeta }$ from $\tilde{S}$, and displaced by a distance $\varepsilon \tilde{𝜍}$ parallel to $\tilde{S}$ [Fig. 2(e)]. By definition of the midsurface, $\tilde{\zeta }=\tilde{𝜍}=0$ if *ζ* = 0. The position of a point in $\tilde{V}$ is thus

(17) $$\tilde{r}(s,\phi ,\zeta )=\tilde{\rho }(s,\phi )+\varepsilon \tilde{\zeta }(s,\zeta )\tilde{n}(s,\phi )+\varepsilon \tilde{𝜍}(s,\zeta ){\tilde{e}}_{s}(s,\phi ).$$

Continuing to use commata to denote partial differentiation, we find

(18a) $$\frac{\partial \tilde{r}}{\partial s}=\left[{\tilde{f}}_{s}\left(1-\varepsilon {\tilde{\kappa }}_{s}\tilde{\zeta }\right)+\varepsilon {\tilde{𝜍}}_{,s}\right]{\tilde{e}}_{s}+\varepsilon \left({\tilde{\zeta }}_{,s}+{\tilde{f}}_{s}{\tilde{\kappa }}_{s}\tilde{𝜍}\right)\tilde{n}$$

and

(18b) $$\frac{\partial \tilde{r}}{\partial \phi }=\left[\tilde{r}\left(1-\varepsilon {\tilde{\kappa }}_{\phi }\tilde{\zeta }\right)+\varepsilon \tilde{𝜍}\cos\tilde{\psi }\right]{\tilde{e}}_{\phi },\frac{\partial \tilde{r}}{\partial \zeta }=\varepsilon \left({\tilde{\zeta }}_{,\zeta }\tilde{n}+{\tilde{𝜍}}_{,\zeta }{\tilde{e}}_{s}\right).$$

Noting that $\tilde{r}={\tilde{f}}_{\phi }r$ from definitions (12), the Riemannian metric of $\tilde{V}$ is therefore

(19a) $$\begin{array}{l} \left\{{\left[{\tilde{f}}_{s}\left(1-\varepsilon {\tilde{\kappa }}_{s}\tilde{\zeta }\right)+\varepsilon {\tilde{𝜍}}_{,s}\right]}^{2}+\varepsilon^{2}{\left({\tilde{\zeta }}_{,s}+{\tilde{f}}_{s}{\tilde{\kappa }}_{s}\tilde{𝜍}\right)}^{2}\right\}\text{d}s^{2}\\ \;+{\left[{\tilde{f}}_{\phi }r\left(1-\varepsilon {\tilde{\kappa }}_{\phi }\tilde{\zeta }\right)+\varepsilon \tilde{𝜍}\cos\tilde{\psi }\right]}^{2}\text{d}\phi^{2}+\varepsilon^{2}\left[{\left({\tilde{\zeta }}_{,\zeta }\right)}^{2}+{\left({\tilde{𝜍}}_{,\zeta }\right)}^{2}\right]\text{d}\zeta^{2}\\ \;+2\varepsilon \left\{{\tilde{𝜍}}_{,\zeta }\left[{\tilde{f}}_{s}\left(1-\varepsilon {\tilde{\kappa }}_{s}\tilde{\zeta }\right)+\varepsilon {\tilde{𝜍}}_{,s}\right]+\varepsilon {\tilde{\zeta }}_{,\zeta }\left({\tilde{\zeta }}_{,s}+{\tilde{f}}_{s}{\tilde{\kappa }}_{s}\tilde{𝜍}\right)\right\}\text{d}s\;\text{d}\zeta . \end{array}$$

From $\tilde{\zeta }=\tilde{𝜍}=0$ on *ζ* = 0, it follows that ${\tilde{\zeta }}_{,s}={\tilde{𝜍}}_{,s}=0$ on *ζ* = 0. Hence the metric of $\tilde{S}$ is simply

(19b) $${\tilde{f}}_{s}^{2}\text{d}s^{2}+{\tilde{f}}_{\phi }^{2}r^{2}\text{d}\phi^{2}.$$

At the surfaces $\tilde{\zeta }=\pm {\tilde{h}}^{\pm }(s)$ of the deformed shell, the unit tangent vectors are ${\tilde{e}}_{s}^{\pm }$ and ${\tilde{e}}_{\phi }^{\pm }={\tilde{e}}_{\phi }$. They define the normals ${\tilde{n}}^{\pm }∥{\tilde{e}}_{s}^{\pm }\times {\tilde{e}}_{\phi }^{\pm }$ [Fig. 2(e)].

#### 3 Intrinsic configuration of the shell: Incompatibility

To specify the intrinsic configuration *𝒱*^0^ of the shell, we introduce the intrinsic stretches $f_{s}^{0},f_{\phi }^{0}$ and the intrinsic curvatures $\kappa_{s}^{0},\kappa_{\phi }^{0}$ and the intrinsic normal displacement *ζ* ^0^. We assume that $f_{s}^{0},f_{\phi }^{0}$ and $\kappa_{s}^{0},\kappa_{\phi }^{0}$ are functions of *s* only, while *ζ* ^0^(*s*, *ζ*) is strictly increasing in *ζ*, with *ζ* ^0^ = 0 on *ζ* = 0. Further, we assume that the analog of the displacement parallel to the midsurface vanishes, *𝜍*^0^ = 0.

Although we have named these functions with reference to similar quantities defined for the deformed configuration, they lack a geometric meaning at this stage. In fact, the Riemannian metric that we can write down by analogy with Eq. (19a),

(20a) $$\begin{array}{l} \left\{{[f_{s}^{0}\left(1-\varepsilon \kappa_{s}^{0}\zeta^{0}\right)]}^{2}+\varepsilon^{2}{\left(\zeta_{\mathrm{,s}}^{0}\right)}^{2}\right\}\text{d}s^{2}+{\left[f_{\phi }^{0}\left(1-\varepsilon \kappa_{\phi }^{0}\zeta^{0}\right)\right]}^{2}r^{2}\text{d}\phi^{2}\\ \;+\varepsilon^{2}{\left(\zeta_{,\zeta }^{0}\right)}^{2}\text{d}\zeta^{2}+2\varepsilon^{2}\zeta_{,\zeta }^{0}\zeta_{\mathrm{,s}}^{0}\;\text{d}s\;\text{d}\zeta , \end{array}$$

is not in general compatible: Its Riemann curvature tensor does not vanish in general, so it cannot, in general, be embedded into three-dimensional Euclidean space [17]. Mechanically, this means that relieving all stresses in the shell requires an infinite number of cuts [17]. This is not surprising because, in the biological system, each cell undergoes independent shape changes or division in general and, since cells are infinitesimal in this continuum description, isolating these infinitesimal building blocks requires infinitely many cuts.

We now define the intrinsic midsurface *S*^0^ of the shell by its Riemannian metric, which is, by analogy with Eq. (19b) and consistently with Eq. (20a),

(20b) $${\left(f_{s}^{0}\right)}^{2}\text{d}s^{2}+{\left(f_{\phi }^{0}\right)}^{2}r^{2}\text{d}\phi^{2}.$$

It follows from a local embedding theorem for Riemannian metrics [30,31] that this two-dimensional metric can be embedded, at least locally, into three-dimensional Euclidean space. In particular, this means that there exists a local (right-handed) orthonormal intrinsic basis *ℬ*^0^ = {***E***_***s***_, ***E***_***ϕ***_, ***N***} of three-dimensional space such that ***E***_***s***_, ***E***_***ϕ***_ = ***u***_***ϕ***_ are tangent to *𝒮*^0^, and ***N*** is normal to it [Fig. 2(f)]. From this basis, we compute the curvatures of *S*^0^, $\varkappa_{s}^{0}=-E_{s}\cdot N_{,s}$ and $\varkappa_{\phi }^{0}=-E_{\phi }\cdot N_{,\phi }$. The intrinsic curvatures $\kappa_{s}^{0},\kappa_{\phi }^{0}$ are specified independently from these as they do not enter the definition of *𝒮*^0^ in Eq. (20b). In particular, $\kappa_{s}^{0},\kappa_{\phi }^{0}$ are in general different from $\varkappa_{s}^{0},\varkappa_{\phi }^{0}$. This expresses the incompatibility of metric (20a). While Eq. (20b) assigns a geometric meaning to the intrinsic stretches $f_{s}^{0},f_{\phi }^{0}$, these intrinsic curvatures therefore remain without the direct geometric realisation that would result from an embedding into three-dimensional Euclidean space, as does the intrinsic normal displacement *ζ* ^0^.

We specify the latter by requiring the intrinsic deformations to conserve volume. This assumption is, for example, appropriate for *Volvox* inversion [Fig. 1(b)]: the cell measurements of Ref. [28] suggest that the cell shape changes driving inversion preserve volume. For other developmental processes that include cell division, the assumption of intrinsic volume conservation would be replaced with a position-dependent constraint that takes account of this growth. Since *ζ* ^0^(*s*, *ζ*) is increasing and can hence be inverted to yield *ζ* (*s*, *ζ* ^0^), Eq. (20a) becomes, on changing coordinates from {*s, ϕ*, *ζ*} to {*s, ϕ*, *ζ*^0^},

(21a) $${\left(\chi_{s}^{0}\right)}^{2}\text{d}s^{2}+{\left(\chi_{\phi }^{0}\right)}^{2}\text{d}\phi^{2}+{\left(\chi_{\zeta^{0}}^{0}\right)}^{2}{\left(\text{d}\zeta^{0}\right)}^{2},$$

with scale factors

(21b) $$\chi_{s}^{0}=f_{s}^{0}(1-\varepsilon \kappa_{s}^{0}\zeta^{0}),\;\chi_{\phi }^{0}\;=f_{\phi }^{0}r(1-\varepsilon \kappa_{\phi }^{0}\zeta^{0}),\;\chi_{\zeta^{0}}^{0}=\varepsilon .$$

Its volume element is therefore

(21c) $$\begin{array}{l} \text{d}V^{0}=\chi_{s}^{0}\chi_{\phi }^{0}\chi_{\zeta^{0}}^{0}\;\text{d}s\;\text{d}\phi \;\text{d}\zeta^{0}\\ \;=\varepsilon f_{s}^{0}f_{\phi }^{0}\left(1-\varepsilon \kappa_{s}^{0}\zeta^{0}\right)\left(1-\varepsilon \kappa_{\phi }^{0}\zeta^{0}\right)r\;\text{d}s\;\text{d}\phi \;\text{d}\zeta^{0}. \end{array}$$

Intrinsic volume conservation requires d*V* = d*V* ^0^, so Eqs. (8c) and (21c) combine to yield a differential equation for *ζ* ^0^ as a function of *ζ*, which we will eventually integrate in Sec. II B under the scaling assumptions of our shell theory.

At this stage, *𝒮*, $\tilde{S}$, and *𝒮*^0^ are defined to be corresponding surfaces within the shell. Indeed, it would it be possible to develop a shell theory for any choice of surfaces that correspond to each other in this way. We add that there is no obvious correspondence between the shell theories that result from different choices of the intrinsic midsurface *𝒮*^0^ belonging to *𝒱*^0^ since the latter cannot be embedded into three-dimensional space.

We now make a particular choice of the surfaces *𝒮*, $\tilde{S}$, and *𝒮*^0^ that, as we shall see in the discussion at the end of Sec. II B, justifies referring to these surfaces as midsurfaces. We do so by imposing the following condition: the surfaces of the shell, at *ζ* = ±*h*^±^(*s*) and $\tilde{\zeta }=\pm {\tilde{h}}^{\pm }(s)$ in *𝒱* and $\tilde{V}$ respectively, correspond to *ζ* ^0^ = ±*h*^0^(*s*)*/*2; the calculations in Sec. II B will show that this choice can be made. We stress that, like *ζ* ^0^, the intrinsic thickness *h*^0^(*s*) lacks a direct geometric realization.

We close by noting that *ζ* ^0^(*s*, *ζ*) and hence *h*^0^(*s*) can also be specified without reference to the incompatible metric (20a), by imposing the condition det **F**^**0**^ = 1. Indeed, with the intrinsic deformation gradient **F**^**0**^ defined as in Eq. (24) below, this is easily seen to be equivalent with d*V* = d*V* ^0^. Conversely, the condition det **F**^**0**^ =1 can be used to define the intrinsic volume element d*V* ^0^ without reference to Eq. (20a).

#### 4 Calculation of the deformation gradient tensors

The geometric deformation gradient is $\tilde{F}=\text{Grad}\;\tilde{r}$ [17], in which the gradient with respect to the undeformed configuration is [17]

(22) $$\text{Grad}\;=\frac{1}{\chi_{s}^{2}}\frac{\partial }{\partial s}⊗\frac{\partial r}{\partial s}+\frac{1}{\chi_{\phi }^{2}}\frac{\partial }{\partial \phi }⊗\frac{\partial r}{\partial \phi }+\frac{1}{\chi_{\zeta }^{2}}\frac{\partial }{\partial \zeta }⊗\frac{\partial r}{\partial \zeta }.$$

Combining Eqs. (7), (8b), and (18), we thus obtain the geometric deformation gradient,

(23) $$\tilde{F}=\left(\begin{array}{ccc} \frac{{\tilde{f}}_{s}\left(1-\varepsilon {\tilde{\kappa }}_{s}\tilde{\zeta }\right)+\varepsilon {\tilde{𝜍}}_{,s}}{1-\varepsilon \varkappa_{s}\zeta } & 0 & {\tilde{𝜍}}_{,\zeta }\\ 0 & \frac{{\tilde{f}}_{\phi }\left(1-\varepsilon {\tilde{\kappa }}_{\phi }\tilde{\zeta }\right)+\varepsilon \tilde{𝜍}\cos\tilde{\psi }/r}{1-\varepsilon \varkappa_{\phi }\zeta } & 0\\ \frac{\varepsilon \left({\tilde{\zeta }}_{,s}+{\tilde{f}}_{s}{\tilde{\kappa }}_{s}\tilde{𝜍}\right)}{1-\varepsilon \varkappa_{s}\zeta } & 0 & {\tilde{\zeta }}_{,\zeta } \end{array}\right),$$

expressed here with respect to the mixed basis $\tilde{ℬ}⊗ℬ$. We now complete specifying the intrinsic configuration *𝒱*^0^ by writing down an analogous expression for the intrinsic deformation gradient with respect to the mixed basis *ℬ*^0^ ⊗ *ℬ*, viz.,

(24) $$F^{0}=\left(\begin{array}{ccc} \frac{f_{s}^{0}\left(1-\varepsilon \kappa_{s}^{0}\zeta^{0}\right)}{1-\varepsilon \varkappa_{s}\zeta } & 0 & 0\\ 0 & \frac{f_{\phi }^{0}\left(1-\varepsilon \kappa_{\phi }^{0}\zeta^{0}\right)}{1-\varepsilon \varkappa_{\phi }\zeta } & 0\\ \frac{\varepsilon \zeta_{,s}^{0}}{1-\varepsilon \varkappa_{s}\zeta } & 0 & \zeta_{,\zeta }^{0} \end{array}\right).$$

The elastic deformation gradient is, therefore, with respect to the natural mixed basis $\tilde{ℬ}⊗{ℬ}^{0}$,

(25) $$F=\tilde{F}{\left({\text{F}}^{0}\right)}^{-1}=(\begin{array}{ccc} \frac{{\tilde{f}}_{s}(1-\varepsilon {\tilde{\kappa }}_{s}\tilde{\zeta })+\varepsilon ({\tilde{𝜍}}_{,s}+{\tilde{𝜍}}_{,\zeta^{0}}\zeta_{,s}^{0})}{f_{s}^{0}(1-\varepsilon \kappa_{s}^{0}\zeta^{0})} & 0 & {\tilde{𝜍}}_{,\zeta^{0}}\\ 0 & \frac{{\tilde{f}}_{\phi }(1-\varepsilon {\tilde{\kappa }}_{\phi }\tilde{\zeta })+\varepsilon \tilde{𝜍}\cos\tilde{\psi }/r}{f_{\phi }^{0}(1-\varepsilon \kappa_{\phi }^{0}\zeta^{0})} & 0\\ \frac{\varepsilon ({\tilde{\zeta }}_{,s}+{\tilde{f}}_{s}{\tilde{\kappa }}_{s}\tilde{𝜍}-\zeta_{,s}^{0}{\tilde{\zeta }}_{,\zeta^{0}})}{f_{s}^{0}(1-\varepsilon \kappa_{s}^{0}\zeta^{0})} & 0 & {\tilde{\zeta }}_{,\zeta^{0}} \end{array}).$$

### B Thin shell theory for large bending deformations

In this subsection, we derive the effective elastic energy for the shell by asymptotic expansion of three-dimensional elasticity. We assume the simplest constitutive law, that the shell is made of an incompressible neo-Hookean material [17], so its elastic energy is

(26) $$ℰ=\;\int \int \int_{V^{0}}\;e\;\text{d}V^{0},\;\;\text{with}\;\;e=\frac{C}{2}({ℐ}_{1}-3),$$

wherein *C >* 0 is a material parameter, and *ℐ*_1_ is the first invariant of the right Cauchy–Green tensor **C** = **F**^⊤^**F** [17]. The integration of the strain energy density *e* is over the intrinsic configuration *𝒱*^0^ of the shell, with volume element d*V* ^0^. As we have noted above, this can be defined from the condition det **F**^**0**^ = 1, independently of the incompatible metric (20a).

The force on an area element $\text{d}\tilde{S}$ with unit normal $\tilde{m}$ of the deformed configuration is $\text{T}\tilde{m}\;\text{d}\tilde{S}$ [17,32]. In this expression, **T** is the Cauchy stress tensor, which, for this neo–Hookean material, is related to the deformation gradient by [21]

(27) $$T=C\left(FF^{⊤}-pI\right),$$

in which **I** is the identity and the Lagrange multiplier *p* is proportional to pressure and imposes the incompressibility condition det **F** = 1. To this area element of the deformed configurations corresponds, in the undeformed configuration, an area element d*S* with unit normal ***m***. Nanson’s relation [17,32] states that $\tilde{m}\;\text{d}\tilde{S}=\tilde{J}{\tilde{\text{F}}}^{-⊤}m\;\text{d}S$, where $\tilde{J}=\det\;\tilde{\text{F}}=\det\;\text{F}\;\det\;F^{0}=1$. We introduce the tensor

(28) $$P=T{\tilde{F}}^{-⊤}=CQ\;\;\text{with}\;\;Q=F{\left(F^{0}\right)}^{-⊤}-p{\tilde{F}}^{-⊤}.$$

In particular, if **F**^**0**^ = **I**, then **P** =**TF**^−T^ is the familiar (first) Piola–Kirchhoff tensor [17]. By definition, $T\tilde{m}\;\text{d}\tilde{S}=Pm\;\text{d}S$, and hence, similarly to the derivation of the familiar Cauchy equation of classical elasticity [17,32], the configuration of the shell minimizing the energy (26) is determined by

(29a) $$\text{Div}\;Q^{⊤}=0,$$

where the divergence (with respect to the undeformed configuration of the shell) is defined by contracting the first and last indices of the gradient in Eq. (22). Since *ℬ* is independent of *ζ* by definition, and using the nabla operator to denote the gradient on *𝒮*, this becomes, on separating the components parallel and perpendicular to the midsurface,

(29b) $$\frac{{\left(\text{Q}n\right)}_{,\zeta }}{\varepsilon }+\nabla \cdot {\text{Q}}^{⊤}=0.$$

#### 1 Scaling assumptions

At this point, we break the complete generality of our description by making scaling assumptions appropriate for a shell theory of large intrinsic bending deformations.

First, we introduce large intrinsic bending deformations explicitly by scaling the intrinsic curvatures so as to allow small radii of curvature in the meridional direction, viz.,

(30) $$\kappa_{s}^{0}=f_{s}^{0}f_{\phi }^{0}\frac{\lambda_{s}^{0}}{\varepsilon },\;\kappa_{\phi }^{0}=f_{s}^{0}f_{\phi }^{0}\lambda_{\phi }^{0},$$

in which the scaled intrinsic curvatures $\lambda_{s}^{0},\lambda_{\phi }^{0}$ are assumed to be *O*(1) quantities. This scaling regime in which the meridional intrinsic radius of curvature becomes comparable to the thickness of the cell sheet is, as shown in Fig. 1(b), the one relevant for *Volvox* invagination, which we shall analyze in Sec. III. Appendix A treats the general case in which all components of the curvature tensor are allowed to be large.

Second, we make the standard scaling assumptions of shell theory, that the elastic strains are small, i.e., that the stretches and curvatures in the deformed configuration do not differ “too much” from the intrinsic stretches and curvatures. In particular, while we have allowed the radius of curvature $1/\kappa_{s}^{0}$ to become comparable to the shell thickness in Eqs. (30), we shall assume the deviations from this to remain small. More formally, we introduce the shell strains *E*_*s*_, *E*_*ϕ*_ by writing

(31) $${\tilde{f}}_{s}=f_{s}^{0}\left(1+\varepsilon E_{s}\right),\;{\tilde{f}}_{\phi }=f_{\phi }^{0}\left(1+\varepsilon E_{\phi }\right),$$

and the curvature strains *L*_*s*_, *L*_*ϕ*_ by letting

(32) $${\tilde{\kappa }}_{s}=f_{s}^{0}f_{\phi }^{0}(\frac{\lambda_{s}^{0}}{\varepsilon }+L_{s}),\;{\tilde{\kappa }}_{\phi }=f_{s}^{0}f_{\phi }^{0}(\lambda_{\phi }^{0}+L_{\phi }).$$

Finally, we introduce the scaled variables

(33) $$Z^{0}=f_{s}^{0}f_{\phi }^{0}\zeta^{0},\;Z=f_{s}^{0}f_{\phi }^{0}\tilde{\zeta },\;S=f_{s}^{0}f_{\phi }^{0}\tilde{\zeta }.$$

While we will come back to discussing the factors $f_{s}^{0}f_{\phi }^{0}$ that arise in Eqs. (30), (32), and (33), we note, for now and from Eq. (20b), the following: the intrinsic midsurface *𝒮*^0^ has surface element $\text{d}S^{0}=f_{s}^{0}f_{\phi }^{0}r\;\text{d}r\;\text{d}\phi =f_{s}^{0}f_{\phi }^{0}\text{d}S$, with d*S* the surface element of the undeformed midsurface *𝒮*. Hence these rescalings by $f_{s}^{0}f_{\phi }^{0}$ absorb the intrinsic stretching of the midsurface. This will turn out to simplify expressions that arise in subsequent calculations.

#### 2 Boundary and incompressibility conditions

We solve the Cauchy equation (29b) subject to the incompressibility condition det **F** =1 and force-free boundary conditions. These boundary conditions, that there be no external forces on the surfaces of the shell, are relevant for many problems in developmental biology, where deformations are, as discussed in the Introduction, driven by changes of the intrinsic geometry only; including external forces does not pose any additional difficulty though.

These force-free boundary conditions read **T**^**±**^***ñ***^**±**^ = **0** [17], where **T**^**±**^ are the Cauchy tensors evaluated on the surfaces $\tilde{\zeta }=\pm {\tilde{h}}^{\pm }$ of $\tilde{V}$. By the above, these are equivalent with **P**^**±**^***n***^**±**^ = **0**, where, from Eq. (28), **P**^**±**^ = *C***Q**^**±**^ are evaluated on the surfaces *ζ* = ±*h*^±^ of *V*, the normal vectors ***n***^**±**^ of which are given by Eqs. (10). The latter yields the expansion

(34) $$n^{\pm }=n\mp \varepsilon h_{,s}^{\pm }e_{s}+O\left(\varepsilon^{2}\right).$$

The incompressibility condition is det **F** = 1. Since the bases $\tilde{ℬ}$ and *ℬ*^0^ are orthonormal, there exist rotations, represented by proper orthogonal matrices $\tilde{\text{R}}$ and R^0^, that map the standard Cartesian basis *χ* onto $\tilde{ℬ}$ and *ℬ*^0^, respectively. Hence, if **F** denotes the matrix in Eq. (25) that represents **F** with respect to the mixed basis $\tilde{ℬ}⊗{ℬ}^{0}$, then **F** is represented by ${\tilde{\text{R}}}^{⊤}{\text{FR}}^{0}$ with respect to *χ* ⊗ *χ*. Since det $\tilde{\text{R}}=\text{det}\;{\text{R}}^{0}=1$, $\text{det}\;\text{F}=\text{det}\left({\tilde{\text{R}}}^{⊤}\text{F}{\text{R}}^{0}\right)=\text{det}\;\text{F}$. The incompressibility condition can therefore be evaluated using the matrix in Eq. (25), but it is important to recognize that incompressibility is a tensorial condition. For the general, nonaxisymmetric deformations discussed in Appendix A, we shall indeed have to distinguish more carefully between tensors and the matrices representing them with respect to mixed nonorthogonal bases, which is why we have already introduced different notations, based on Ogden’s [32], for matrices (sans serif font) and tensors (bold sans serif font) that could be used interchangeably here.

#### 3 Intrinsic volume conservation

Before expanding the boundary and incompressibility conditions asymptotically, we determine the dependence of *ζ* ^0^ and hence *Z*^0^ on *ζ* that results from the condition d*V* = d*V* ^0^ of intrinsic volume conservation. On recalling that $\kappa_{s}^{0}=O\left(\varepsilon^{-1}\right)$, the expressions for d*V* in Eq. (8c) and d*V* ^0^ in Eq. (21c) yield, at leading order, a differential equation for *Z*^0^(*ζ*),

(35) $$\left(1-\lambda_{s}^{0}Z^{0}\right)Z_{,\zeta }^{0}=1\Rightarrow Z^{0}=\frac{1}{\lambda_{s}^{0}}\left(1-\sqrt{1-2\lambda_{s}^{0}\zeta }\right),$$

where we have imposed *Z*^0^ = 0 at *ζ* = 0. Let $H^{0}=h^{0}f_{s}^{0}f_{\phi }^{0}$. Since *ζ* ^0^ = ±*h*^0^*/*2 ⇔ *Z*^0^ = ±*H* ^0^*/*2 at *ζ* = ±*h*^±^ by definition, Eq. (35) implies

(36) $$h^{\pm }=\frac{H^{0}}{2}\left(1\mp \frac{\lambda_{s}^{0}}{4}H^{0}\right)\;\Rightarrow \;h=h^{+}+h^{-}=H^{0},\;$$

wherein *h* is again the undeformed thickness of the cell sheet [Fig. 2(c)]. We note that Eq. (36) is a leading-order result only, since we have ignored *O*(*ε*) corrections in Eq. (35).

#### 4 Expansion of the boundary and incompressibility conditions

To expand the incompressibility and boundary conditions in the small parameter *ε*, we posit regular expansions

(37) $$Z=Z_{\left(0\right)}+\varepsilon Z_{\left(1\right)}+O(\varepsilon^{2}),\;S=S_{\left(0\right)}+O(\varepsilon ),$$

for the scaled transverse and parallel displacements. Throughout this paper, we shall use subscripts in parentheses in this way to denote the different terms in asymptotic expansions in *ε*. We further expand

(38) $$Q=Q_{\left(0\right)}+\varepsilon Q_{\left(1\right)}+O(\varepsilon^{2}),\;p=p_{\left(0\right)}+O(\varepsilon ).$$

##### (a) Expansion at order O(1)

At leading order, Eq. (29b) yields (**Q**_**(0)**_***n***)_,*ζ*_ = **0**, so **Q**_**(0)**_***n*** = ***Q***(*s*) is independent of *ζ*. It follows that $0={\text{Q}}^{\pm }n^{\pm }={\text{Q}}_{\left(0\right)}^{\pm }n+O(\varepsilon )=\pm Q+O(\varepsilon )$ using Eq. (34). Thus $0\equiv Q={\text{Q}}_{\left(0\right)}n=\left(q_{\left(0\right)}^{s},0,q_{\left(0\right)}^{n}\right)$. Expanding definition (28) using Eqs. (23)–(25), this yields [33]

(39a) $$0=q_{\left(0\right)}^{s}=f_{s}^{0}f_{\phi }^{0}\left(1-\lambda_{s}^{0}Z^{0}\right)\frac{\lambda_{s}^{0}S_{\left(0\right)}\left[p_{\left(0\right)}-{\left(S_{(0),Z^{0}}\right)}^{2}\right]+\left(1-\lambda_{s}^{0}Z_{\left(0\right)}\right)S_{(0),Z^{0}}Z_{(0),Z^{0}}}{\left(1-\lambda_{s}^{0}Z_{\left(0\right)}\right)Z_{(0),Z^{0}}-\lambda_{s}^{0}S_{\left(0\right)}S_{(0),Z^{0}}},$$

(39b) $$0=q_{\left(0\right)}^{n}=f_{s}^{0}f_{\phi }^{0}\left(1-\lambda_{s}^{0}Z^{0}\right)\frac{\left(1-\lambda_{s}^{0}Z_{\left(0\right)}\right)\left[{\left(Z_{(0),Z^{0}}\right)}^{2}-p_{\left(0\right)}\right]-\lambda_{s}^{0}S_{\left(0\right)}S_{(0),Z^{0}}Z_{(0),Z^{0}}}{\left(1-\lambda_{s}^{0}Z_{\left(0\right)}\right)Z_{(0),Z^{0}}-\lambda_{s}^{0}S_{\left(0\right)}S_{(0),Z^{0}}},$$

where we have used ${\left(\zeta^{0}{,}_{\zeta }\right)}^{-1}=f_{s}^{0}f_{\phi }^{0}\left(1-\lambda_{s}^{0}Z^{0}\right)+O(\varepsilon )$, which follows from Eq. (35) on recalling the rescalings (33). Moreover, on expanding the incompressibility condition using Eq. (25), we find

(40) $$1=\text{det}\;F=1-\frac{1-\lambda_{s}^{0}Z^{0}-\left(1-\lambda_{s}^{0}Z_{\left(0\right)}\right)Z_{(0),Z^{0}}+\lambda_{s}^{0}S_{\left(0\right)}S_{(0),Z^{0}}}{1-\lambda_{s}^{0}Z^{0}}+O(\varepsilon ).\;$$

Equations (39) and (40) define a system of three simultaneous linear algebraic equations for *p*_(0)_,$Z_{(0),Z^{0}}$, and $S_{(0),Z^{0}}$, with solution

(41a) $$p_{\left(0\right)}=\frac{{\left(1-\lambda_{s}^{0}Z^{0}\right)}^{2}}{{\left(1-\lambda_{s}^{0}Z_{\left(0\right)}\right)}^{2}+{\left(\lambda_{s}^{0}S_{\left(0\right)}\right)}^{2}},$$

(41b) $$Z_{(0),Z^{0}}=\frac{\left(1-\lambda_{s}^{0}Z^{0}\right)\left(1-\lambda_{s}^{0}Z_{\left(0\right)}\right)}{{\left(1-\lambda_{s}^{0}Z_{\left(0\right)}\right)}^{2}+{\left(\lambda_{s}^{0}S_{\left(0\right)}\right)}^{2}},$$

(41c) $$S_{(0),Z^{0}}=-\frac{\lambda_{s}^{0}S_{\left(0\right)}\left(1-\lambda_{s}^{0}Z^{0}\right)}{{\left(1-\lambda_{s}^{0}Z_{\left(0\right)}\right)}^{2}+{\left(\lambda_{s}^{0}S_{\left(0\right)}\right)}^{2}}.$$

Equation (40) or Eqs. (41b) and (41c) imply

(42a) $$-2Z_{(0),Z^{0}}\left(1-\lambda_{s}^{0}Z_{\left(0\right)}\right)+2\lambda_{s}^{0}S_{\left(0\right)}S_{(0),Z^{0}}=-2\left(1-\lambda_{s}^{0}Z^{0}\right).$$

Integrating and using the fact that *Z*_(0)_ = *S*_(0)_ = 0 at *Z*^0^ = 0 by definition of the midsurfaces, we obtain

(42b) $${\left(1-\lambda_{s}^{0}Z_{\left(0\right)}\right)}^{2}+{\left(\lambda_{s}^{0}S_{\left(0\right)}\right)}^{2}={\left(1-\lambda_{s}^{0}Z^{0}\right)}^{2}.\;$$

Equation (41a) now becomes *p*_(0)_ = 1. Moreover, on substituting Eq. (42b) into Eq. (41b),

(43) $$\frac{\partial Z_{\left(0\right)}}{\partial Z^{0}}=\frac{1-\lambda_{s}^{0}Z_{\left(0\right)}}{1-\lambda_{s}^{0}Z^{0}}\;\Rightarrow \;\frac{1-\lambda_{s}^{0}Z_{\left(0\right)}}{1-\lambda_{s}^{0}Z^{0}}=\;\text{const}.,$$

which, using *Z*_(0)_ = 0 at *Z*^0^ = 0 again, yields *Z*_(0)_ ≡ *Z*^0^. Hence *S*_(0)_ ≡ 0 from Eq. (42b). The last equality is the Kirchhoff “hypothesis” [15]: normals to the intrinsic midsurface remain, at lowest order, normal to the deformed midsurface.

##### (b) Expansion at order O(ε)

We now expand the incompressibility condition further, finding

(44) $$0=\det\text{F}-1=\varepsilon \left(E_{s}+E_{\phi }-L_{\phi }Z^{0}+\frac{\partial Z_{\left(1\right)}}{\partial Z^{0}}-\frac{L_{s}Z^{0}+\lambda_{s}^{0}Z_{\left(1\right)}}{1-\lambda_{s}^{0}Z^{0}}\right)+O\left(\varepsilon^{2}\right).$$

On solving the resulting differential equation for *Z*_(1)_ by imposing *Z*_(1)_ = 0 at *Z*^0^ = 0, we obtain

(45) $$Z_{\left(1\right)}=-\frac{Z^{0}\left\{6\left(E_{s}+E_{\phi }\right)-3Z^{0}\left[L_{s}+L_{\phi }+\lambda_{s}^{0}\left(E_{s}+E_{\phi }\right)\right]+2\lambda_{s}^{0}L_{\phi }{\left(Z^{0}\right)}^{2}\right\}}{6\left(1-\lambda_{s}^{0}Z^{0}\right)}.$$

##### (c) Expansion at order O(ε^2^)

It will turn out not to be necessary to expand the deformation gradient explicitly beyond order *O*(*ε*). Indeed, it will suffice to consider a formal expansion,

(46) $$F=\left(\begin{array}{ccc} 1+\varepsilon a_{\left(1\right)}+\varepsilon^{2}a_{\left(2\right)}+O\left(\varepsilon^{3}\right) & 0 & \varepsilon v_{\left(1\right)}+O\left(\varepsilon^{2}\right)\\ 0 & 1+\varepsilon b_{\left(1\right)}+\varepsilon^{2}b_{\left(2\right)}+O\left(\varepsilon^{3}\right) & 0\\ \varepsilon w_{\left(1\right)}+O\left(\varepsilon^{2}\right) & 0 & 1+\varepsilon c_{\left(1\right)}+\varepsilon^{2}c_{\left(2\right)}+O\left(\varepsilon^{3}\right) \end{array}\right),$$

with the leading-order terms found from Eq. (25). This also yields, using Eq. (45),

(47) $$a_{\left(1\right)}=\frac{6E_{s}-6\;[L_{s}+\lambda_{s}^{0}(E_{s}-E_{\phi })]\;Z^{0}+3\lambda_{s}^{0}[L_{s}-L_{\phi }+\lambda_{s}^{0}(E_{s}-E_{\phi })]{\left(Z^{0}\right)}^{2}+2{\left(\lambda_{s}^{0}\right)}^{2}L_{\phi }{\left(Z^{0}\right)}^{3}}{6\;{\left(1-\lambda_{s}^{0}Z^{0}\right)}^{2}},\;b_{\left(1\right)}=E_{\phi }-Z^{0}L_{\phi }.$$

Expressions for *a*_(2)_, *b*_(2)_, *c*_(1)_, *c*_(2)_, *v*_(1)_, *w*_(1)_ could similarly be obtained in terms of the expansions (37), but, as announced, will turn out to be of no consequence. Using Eq. (46), the incompressibility condition becomes

(48) $$1=\det\text{F}=1+\varepsilon (a_{\left(1\right)}+b_{\left(1\right)}+c_{\left(1\right)})+\varepsilon^{2}(a_{\left(2\right)}+b_{\left(2\right)}+c_{\left(2\right)}+a_{\left(1\right)}b_{\left(1\right)}+b_{\left(1\right)}c_{\left(1\right)}+c_{\left(1\right)}a_{\left(1\right)}-v_{\left(1\right)}w_{\left(1\right)})+O(\varepsilon^{3}).$$

Next, using Eq. (24), we introduce an analogous formal expansion for the intrinsic deformation gradient, viz.,

(49) $$F^{0}=\left(\begin{array}{ccc} a_{\left(0\right)}^{0}+O(\varepsilon ) & 0 & 0\\ 0 & b_{\left(0\right)}^{0}+O(\varepsilon ) & 0\\ \varepsilon w_{\left(1\right)}^{0}+O\left(\varepsilon^{2}\right) & 0 & c_{\left(0\right)}^{0}+O(\varepsilon ) \end{array}\right),$$

Where $c_{\left(0\right)}^{0}={\left[f_{s}^{0}f_{\phi }^{0}\left(1-\lambda_{s}^{0}Z^{0}\right)\right]}^{-1}$ using Eq. (35), and the values of $a_{\left(0\right)}^{0},b_{\left(0\right)}^{0},w_{\left(1\right)}^{0}$ are of no consequence. Hence, using Eq. (46),

(50) $$\tilde{F}=FF^{0}=\left(\begin{array}{ccc} a_{\left(0\right)}^{0}+O(\varepsilon ) & 0 & \varepsilon c_{\left(0\right)}^{0}v_{\left(1\right)}+O\left(\varepsilon^{2}\right)\\ 0 & b_{\left(0\right)}^{0}+O(\varepsilon ) & 0\\ \varepsilon \left(w_{\left(1\right)}^{0}+a_{\left(0\right)}^{0}w_{\left(1\right)}\right)+O\left(\varepsilon^{2}\right) & 0 & c_{\left(0\right)}^{0}+O(\varepsilon ). \end{array}\right),$$

and thus, since *p* = 1 + *O*(*ε*),

(51) $$\text{Q}=\left(\begin{array}{ccc} O(\varepsilon ) & 0 & \varepsilon \left(v_{\left(1\right)}+w_{\left(1\right)}\right)/c_{\left(0\right)}^{0}+O\left(\varepsilon^{2}\right)\\ 0 & O(\varepsilon ) & 0\\ O(\varepsilon ) & 0 & O(\varepsilon ). \end{array}\right)\;\Rightarrow \;{\text{Q}}_{\left(0\right)}=\text{O},\;{\text{Q}}_{\left(1\right)}n=\left(\begin{array}{c} \left(v_{\left(1\right)}+w_{\left(1\right)}\right)/c_{\left(0\right)}^{0}\\ 0\\ O(1) \end{array}\right).$$

In particular, Eq. (29b) at order *O*(1) is just (**Q**_**(1)**_***n***)_,*ζ*_ = **0**. Moreover $0={\text{Q}}^{\pm }n^{\pm }=\varepsilon {\text{Q}}_{\left(1\right)}^{\pm }n+O\left(\varepsilon^{2}\right)$, since **Q**_**(0)**_ = **O** and using Eq. (34). Similarly to above, this implies **Q**_**(1)**_***n*** ≡ **0**. From this and from Eq. (48), we infer

(52a) $$w_{\left(1\right)}=-v_{\left(1\right)},\;c_{\left(1\right)}=-\;(a_{\left(1\right)}+b_{\left(1\right)}),$$

(52b) $$c_{\left(2\right)}=a_{\left(1\right)}^{2}+a_{\left(1\right)}b_{\left(1\right)}+b_{\left(1\right)}^{2}-a_{\left(2\right)}-b_{\left(2\right)}+v_{\left(1\right)}w_{\left(1\right)}.\;$$

#### 5 Asymptotic expansion of the constitutive relations

On computing the expansion of **C** = **F**^⊤^**F** from Eq. (46) and hence that of *ℐ*_1_= tr **C**, and simplifying using Eqs. (52), we obtain

(53a) $$\begin{array}{l} {ℐ}_{1}=3+\varepsilon [2(a_{\left(1\right)}+b_{\left(1\right)}+c_{\left(1\right)})]+\varepsilon^{2}[a_{\left(1\right)}^{2}+b_{\left(1\right)}^{2}+c_{\left(1\right)}^{2}+v_{\left(1\right)}^{2}+w_{\left(1\right)}^{2}+2(a_{\left(2\right)}+b_{\left(2\right)}+c_{\left(2\right)})]+O(\varepsilon^{3})\\ \;=3+\varepsilon^{2}[4(a_{\left(1\right)}^{2}+a_{\left(1\right)}b_{\left(1\right)}+b_{\left(1\right)}^{2})]+O(\varepsilon^{3}).\; \end{array}$$

Hence, from Eqs. (47) and on introducing $x=\lambda_{s}^{0}Z^{0}$,

(53b) $$\begin{array}{l} {ℐ}_{1}=3+\frac{\varepsilon^{2}}{{\left(1-x\right)}^{4}}\{{\left[1+{\left(1-x\right)}^{2}\right]}^{2}E_{s}^{2}+2[1+{\left(1-x\right)}^{2}]E_{s}E_{\phi }+(4-12x+18x^{2}-12x^{3}+3x^{4})E_{\phi }^{2}\\ \;\;-\frac{1}{\lambda_{s}^{0}}[2x(4-6x+4x^{2}-x^{3})E_{s}L_{s}-2x(2-x)E_{\phi }L_{s}-\frac{2x}{3}(6-12x+11x^{2}-5x^{3}+x^{4})E_{s}L_{\phi }\\ \;-\frac{2x}{3}(12-39x+55x^{2}-36x^{3}+9x^{4})E_{\phi }L_{\phi }]\\ \;+\frac{1}{{\left(\lambda_{s}^{0}\right)}^{2}}[x^{2}{\left(2-x\right)}^{2}L_{s}^{2}+\frac{2x^{2}}{3}(6-9x+5x^{2}-x^{3})L_{s}L_{\phi }+\frac{x^{2}}{9}(36-126x+177x^{2}-114x^{3}+28x^{4})L_{\phi }^{2}]\}\\ \;+O(\varepsilon^{3}). \end{array}$$

This determines the leading-order term in the asymptotic expansion of the energy density in Eqs. (26). On defining, from Eq. (46), the (symmetric) effective two-dimensional deformation gradient and associated two-dimensional strain,

(54) $$\hat{F}=\left(\begin{array}{cc} 1+\varepsilon a_{\left(1\right)} & 0\\ 0 & 1+\varepsilon b_{\left(1\right)} \end{array}\right)+O\left(\varepsilon^{2}\right),\;\hat{E}=\frac{{\hat{F}}^{⊤}\hat{F}-I}{2\varepsilon },$$

wherein **I** is again the identity, we rewrite Eq. (53a) as

(55) $${ℐ}_{1}-3=2\varepsilon^{2}\left[{\left(\text{tr}\;\hat{E}\right)}^{2}+\text{tr}\;{\hat{E}}^{2}\right]+O\left(\varepsilon^{3}\right)$$

This shows how, at leading order, the energy density depends only on the two invariants of the effective two-dimensional strain. In the asymptotic limit of a thin shell, the constitutive relations have thus become effectively two-dimensional.

#### 6 Derivation of the thin shell theory

We are now set up to average out the transverse coordinate and thus obtain the thin shell theory. We compute, from Eq. (21c), the leading-order expansion for the volume element in the intrinsic configuration:

(56) $$\begin{array}{l} \text{d}V^{0}=\varepsilon (1-\lambda_{s}^{0}Z^{0})r\;\text{d}s\;\text{d}\phi \;\text{d}Z^{0}+O(\varepsilon^{2})\\ \;=\frac{1-x}{\lambda_{s}^{0}}\varepsilon \;r\;\text{d}s\;\text{d}\phi \;\text{d}x+O(\varepsilon^{2}). \end{array}$$

Moreover, we introduce $\eta =\lambda_{s}^{0}h/2$, so that the shell surfaces *ζ* ^0^ = ±*h*^0^/2 correspond to *x* = ±*η*.

On substituting Eqs. (53b) and (56) into Eqs. (26), integrating with respect to *x*, and using axisymmetry, we then obtain

(57a) $$ℰ=\;\iint_{S}\hat{e}\;r\;\text{d}s\;\text{d}\phi =2\pi \;\int_{C}\hat{e}\;r\;\text{d}s,$$

with the first integration over the undeformed axisymmetric midsurface *𝒮* and the second over the curve *𝒞* generating *𝒮*. The effective two-dimensional energy density *ê* in Eq. (57a) is

(57b) $$\begin{array}{l} \hat{e}=\frac{\varepsilon }{\lambda_{s}^{0}}\int_{-\eta }^{\eta }e(x)(1\;-x)dx=\frac{C}{2}\varepsilon^{3}\{h[\alpha_{ss}E_{s}^{2}+(\alpha_{s\phi }+\alpha_{\phi s})E_{s}E_{\phi }+\alpha_{\phi \phi }E_{\phi }^{2}]+2h^{2}[\beta_{ss}E_{s}L_{s}+\beta_{s\phi }E_{s}L_{\phi }+\beta_{\phi s}E_{\phi }L_{s}+\beta_{\phi \phi }E_{\phi }L_{\phi }]\;+h^{3}[\gamma_{ss}L_{s}^{2}+(\gamma_{s\phi }+\gamma_{\phi s})L_{s}L_{\phi }+\gamma_{\phi \phi }L_{\phi }^{2}]\}+O(\varepsilon^{4}), \end{array}$$

wherein

(58a) $$\alpha_{ss}=\frac{\eta^{4}-2\eta^{2}+2}{{\left(1-\eta^{2}\right)}^{2}}+\frac{2\;{\text{tanh}}^{-1}\;\eta }{\eta },$$

(58b) $$\alpha_{s\phi }=\alpha_{\phi s}=\frac{1}{{\left(1-\eta^{2}\right)}^{2}}+\frac{{\text{tanh}}^{-1}\;\eta }{\eta },$$

(58c) $$\alpha_{\phi \phi }=\frac{3\eta^{4}-6\eta^{2}+4}{{\left(1-\eta^{2}\right)}^{2}},$$

(58d) $$\beta_{ss}=-\frac{\eta \left(2-\eta^{2}\right)}{2{\left(1-\eta^{2}\right)}^{2}},$$

(58e) $$\beta_{s\phi }=\frac{\eta^{6}+4\eta^{4}-11\eta^{2}+3}{18\eta {\left(1-\eta^{2}\right)}^{2}}-\frac{{\text{tanh}}^{-1}\;\eta }{6\eta^{2}},$$

(58f) $$\beta_{\phi s}=-\frac{1}{2\eta {\left(1-\eta^{2}\right)}^{2}}+\frac{{\text{tanh}}^{-1}\;\eta }{2\eta^{2}},$$

(58g) $$\beta_{\phi \phi }=\frac{3\eta^{5}-5\eta^{3}+\eta }{6{\left(1-\eta^{2}\right)}^{2}},$$

(58h) $$\gamma_{ss}=\frac{\eta^{4}-2\eta^{2}+2}{4\eta^{2}{\left(1-\eta^{2}\right)}^{2}}-\frac{{\text{tanh}}^{-1}\;\eta }{2\eta^{3}},$$

(58i) $$\gamma_{s\phi }=\gamma_{\phi s}=\frac{\eta^{6}-2\eta^{4}+\eta^{2}+3}{36\eta^{2}{\left(1-\eta^{2}\right)}^{2}}-\frac{{\text{tanh}}^{-1}\;\eta }{12\eta^{3}},$$

(58j) $$\gamma_{\phi \phi }=\frac{10\eta^{4}-21\eta^{2}+12}{36{\left(1-\eta^{2}\right)}^{2}}$$

are functions of the large bending parameter

(59) $$\eta =\frac{\lambda_{s}^{0}}{2}h=\frac{\kappa_{s}^{0}}{2f_{s}^{0}f_{\phi }^{0}}(\varepsilon h)=\frac{\kappa_{s}^{0}}{2}\left(\varepsilon h^{0}\right)$$

only. Moreover, from Eqs. (31) and (32), the shell strains in Eq. (57b) are

(60) $$\varepsilon E_{s}=\frac{{\tilde{f}}_{s}-f_{s}^{0}}{f_{s}^{0}},\;\varepsilon E_{\phi }=\frac{{\tilde{f}}_{\phi }-f_{\phi }^{0}}{f_{\phi }^{0}},$$

while the curvature strains are

(61a) $$L_{s}=\frac{{\tilde{\kappa }}_{s}-\kappa_{s}^{0}}{f_{s}^{0}f_{\phi }^{0}}=K_{s}-\frac{2\eta }{h}E_{s}+O(\varepsilon ),$$

(61b) $$L_{\phi }=\frac{{\tilde{\kappa }}_{\phi }-\kappa_{\phi }^{0}}{f_{s}^{0}f_{\phi }^{0}}=K_{\phi }+O(\varepsilon ),$$

where we have defined

(62) $$K_{s}=\frac{{\tilde{f}}_{s}{\tilde{\kappa }}_{s}-f_{s}^{0}\kappa_{s}^{0}}{{\left(f_{s}^{0}\right)}^{2}f_{\phi }^{0}},\;K_{\phi }=\frac{{\tilde{f}}_{\phi }{\tilde{\kappa }}_{\phi }-f_{\phi }^{0}\kappa_{\phi }^{0}}{f_{s}^{0}{\left(f_{\phi }^{0}\right)}^{2}}.$$

Shell theories are expressed more naturally in terms of the alternative curvature strains *K*_*s*_, *K*_*ϕ*_. Indeed, *K*_*s*_, *K*_*ϕ*_ vanish for pure stretching deformations, whereas *L*_*s*_, *L*_*ϕ*_ do not: Consider a shell, the undeformed (and intrinsic) configuration of which is a sphere of radius *R*, and which deforms into a sphere of radius *R*′ = *f R*, for example because of a pressure difference between the inside and outside. For this deformation, $f_{s}^{0}=f_{\phi }^{0}=1$, $\kappa_{s}^{0}=\kappa_{\phi }^{0}=1/R$, while ${\tilde{f}}_{s}={\tilde{f}}_{\phi }=f,\;{\tilde{\kappa }}_{s}={\tilde{\kappa }}_{\phi }=1/fR$ and so *L*_*s*_ = *L*_*ϕ*_ = (1 − *f*)/*f* ^3^*R* ≠ 0 for *f* ≠ 1, but *K*_*s*_ = *K*_*ϕ*_ = 0. Reference [15] has also discussed this point, noting that *L*_*s*_, *L*_*ϕ*_ and *K*_*s*_, *K*_*ϕ*_ can be used interchangeably in classical shell theories. However, Eq. (61a) shows that, in the large bending limit considered here, *L*_*s*_ − *K*_*s*_ = *O*(1). Even at leading order, the stretching deformations associated with changes in curvature cannot therefore be neglected in this limit. In terms of the alternative curvature strains *K*_*s*_, *K*_*ϕ*_, Eq. (57b) becomes

(63) $$\begin{array}{l} \hat{e}=\frac{C}{2}\varepsilon^{3}\{h\left[{\bar{\alpha }}_{ss}E_{s}^{2}+\left({\bar{\alpha }}_{s\phi }+{\bar{\alpha }}_{\phi s}\right)E_{s}E_{\phi }+\alpha_{\phi \phi }E_{\phi }^{2}\right]\\ \;+2h^{2}\left[{\bar{\beta }}_{ss}E_{s}K_{s}+{\bar{\beta }}_{s\phi }E_{s}K_{\phi }+\beta_{\phi s}E_{\phi }K_{s}+\beta_{\phi \phi }E_{\phi }K_{\phi }\right]\\ \;+h^{3}\left[\gamma_{ss}K_{s}^{2}+\left(\gamma_{s\phi }+\gamma_{\phi s}\right)K_{s}K_{\phi }+\gamma_{\phi \phi }K_{\phi }^{2}\right]\}+O\left(\varepsilon^{4}\right), \end{array}$$

where *α*_*ϕϕ*_, *β*_*ϕs*_, *β*_*ϕϕ*_, *γ*_*ss*_, *γ*_*sϕ*_ *= γ*_*ϕs*_, *γ*_*ϕϕ*_ are still given by Eqs. (58), while

(64a) $${\bar{\alpha }}_{ss}=\alpha_{ss}-4\eta \beta_{ss}+4\eta^{2}\gamma_{ss}=\frac{4}{{\left(1-\eta^{2}\right)}^{2}},$$

(64b) $${\bar{\alpha }}_{s\phi }={\bar{\alpha }}_{\phi s}=\alpha_{s\phi }-2\eta \beta_{\phi s}=\frac{2}{{\left(1-\eta^{2}\right)}^{2}},$$

(64c) $${\bar{\beta }}_{ss}=\beta_{ss}-2\eta \gamma_{ss}=-\frac{1}{\eta {\left(1-\eta^{2}\right)}^{2}}+\frac{\tanh^{-1}\;\eta }{\eta^{2}},$$

(64d) $${\bar{\beta }}_{s\phi }=\beta_{s\phi }-2\eta \gamma_{s\phi }=-\frac{\eta \left(2-\eta^{2}\right)}{3{\left(1-\eta^{2}\right)}^{2}}.$$

This completes the derivation of the elastic energy (57a) of a thin shell undergoing large axisymmetric bending deformations. In Appendix B, we derive the associated governing equations, using the expression (63) of the energy density in terms of the alternative curvature strains defined in Eqs. (62).

#### 7 Discussion

Several features of the shell theory that we have obtained here are worth discussing in some detail.

##### (a) Stretching, coupling, and bending energies

The terms that appear in the elastic energy (63) separate into stretching, coupling, and bending terms, viz.,

(65) $$\hat{e}={\hat{e}}_{\text{stretch}\;}+{\hat{e}}_{\text{couple}\;}+{\hat{e}}_{\text{bend}\;}+O\left(\varepsilon^{4}\right),\;$$

with

(66a) $${\hat{e}}_{\text{stretch}\;}=\frac{Ch}{2}\varepsilon^{3}\left[{\bar{\alpha }}_{ss}E_{s}^{2}+\left({\bar{\alpha }}_{s\phi }+{\bar{\alpha }}_{\phi s}\right)E_{s}E_{\phi }+\alpha_{\phi \phi }E_{\phi }^{2}\right],$$

(66b) $${\hat{e}}_{\text{couple}\;}=Ch^{2}\varepsilon^{3}\left[{\bar{\beta }}_{ss}E_{s}K_{s}+{\bar{\beta }}_{s\phi }E_{s}K_{\phi }+\beta_{\phi s}E_{\phi }K_{s}+\beta_{\phi \phi }E_{\phi }K_{\phi }\right],$$

(66c) $${\hat{e}}_{\text{bend}\;}=\frac{Ch^{3}}{2}\varepsilon^{3}\left[\gamma_{ss}K_{s}^{2}+\left(\gamma_{s\phi }+\gamma_{\phi s}\right)K_{s}K_{\phi }+\gamma_{\phi \phi }K_{\phi }^{2}\right].\;$$

As ${\left({\bar{\alpha }}_{s\phi }+{\bar{\alpha }}_{\phi s}\right)}^{2}-4{\bar{\alpha }}_{ss}{\bar{\alpha }}_{\phi \phi }=-\;48{\left(1-\eta^{2}\right)}^{-2}<0$ for |*η*| < 1, the stretching energy *ê*_stretch_ is positive semidefinite. Numerically, we also find that (*γ*_*sϕ*_ + *γ*_*ϕs*_)^2^ − 4*γ*_*ss*_*γ*_*ϕϕ*_ < 0 for |*η*| < 1, and hence the bending energy *ê*_bend_ is positive semidefinite, too. However, the coupling energy *ê*_couple_ can clearly be of either sign, though *ê* is of course positive semidefinite.

##### (b) Constriction limit: Divergence

All of the coefficient functions in Eqs. (58) and (64) diverge as *η* → ±1. More precisely, the coefficients diverge like (1 − |*η*|)^−2^, and so Eq. (63) loses asymptoticity when $1-|\eta |=O(\sqrt{\varepsilon })$, and hence the shell theory is not formally valid in this limit. This is mirrored by a similar breakdown of asymptoticity at other places in the analysis: for example, Eqs. (47) show that the expansion of the deformation gradient in Eq. (46) also breaks down when $1-|\eta |=O(\sqrt{\varepsilon })$. However, this divergence, absent from theories not valid for large bending deformations, is not surprising in the first place. Indeed, the limit *η* → ± 1 corresponds to constricted cells, i.e., wedge-shaped, triangular cells [Fig. 1(b), inset] for which the intrinsic meridional radius of curvature is half the intrinsic cell sheet thickness: one of the surfaces of the shell has contracted to a point in the intrinsic configuration, so is geometrically singular. As the intrinsic configuration approaches this constricted limit somewhere, deviations from the intrinsic configuration become more and more expensive energetically there compared to other positions in the shell, unless the divergence of *ê* as *η* → ±1 is suppressed. This happens if *ê*_couple_ ≈ − (*ê*_stretch_ + *ê*_bend_) < 0 or the divergence of each of *ê*_stretch_, *ê*_couple_, *ê*_bend_ is suppressed, which is possible for special values of *E*_*s*_, *E*_*ϕ*_, *K*_*s*_, *K*_*ϕ*_, as discussed in more detail below.

##### (c) Geometric anisotropy

**Fig. 3:**
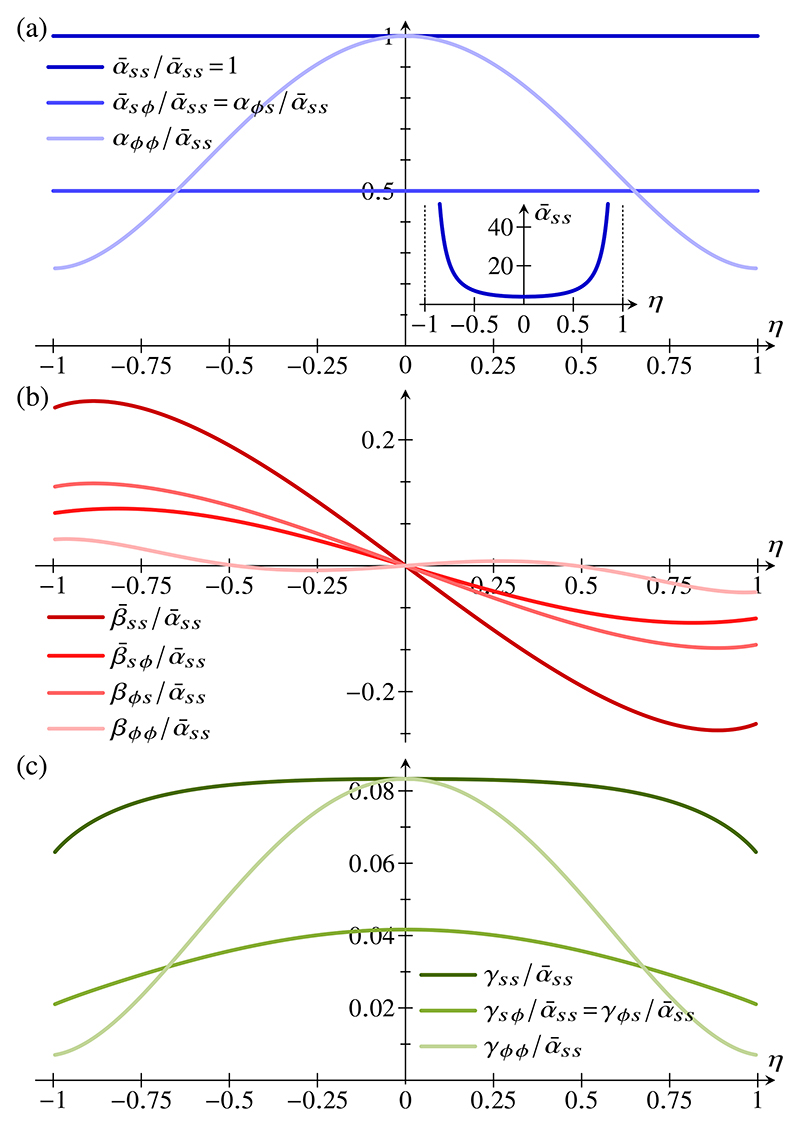
Effective two-dimensional energy density. Plots of the coefficients in Eq. (63), defined in Eqs. (58) and (64), against *η*. All coefficients are arbitrarily scaled with ${\bar{\alpha }}_{ss}$ to absorb their divergence in the constriction limit *η* → ±1. (a) Plot of the stretching coefficients ${\bar{\alpha }}_{ss},{\bar{\alpha }}_{s\phi },{\bar{\alpha }}_{\phi s}$, *α*_*ϕϕ*_. Inset: Unscaled plot of ${\bar{\alpha }}_{ss}$ against *η*, diverging as *η* → ±1. (b) Plot of the mixed co-efficients ${\bar{\beta }}_{ss},{\bar{\beta }}_{s\phi }$, *β*_*ϕs*_, *β*_*ϕϕ*_. (c) Plot of the bending coefficients *γ*_*ss*_, *γ*_*sϕ*_, *γ*_*ϕs*_, *γ*_*ϕϕ*_.

Plots of the coefficient functions in Eqs. (58) and (64), arbitrarily scaled with ${\bar{\alpha }}_{ss}$ to absorb their divergence as *η* → ±1, are shown in Fig. 3. These illustrate how the relative importance of different deformation modes depends on the amount of intrinsic bending. In other words, large bending deformations break the material isotropy, so that different directions of stretching have different effective stretching moduli; similarly, different effective bending moduli are associated with different directions of bending. This anisotropy is therefore geometric. It is not perhaps surprising since it mirrors the curvature anisotropy of the intrinsic configuration but, as discussed below, this effect is absent from the classical theories not valid for large bending deformations.

##### (d) Midsurfaces

The leading-order solution above shows that $\tilde{\zeta }=\zeta^{0}+O(\varepsilon )$. This implies that ${\tilde{h}}^{\pm }=h^{0}/2+O(\varepsilon )$, and hence $\left({\tilde{h}}^{+}-{\tilde{h}}^{-}\right)/2=O(\varepsilon )$. The middle surface $\tilde{\zeta }=\left({\tilde{h}}^{+}-{\tilde{h}}^{-}\right)/2$ of the deformed configuration $\tilde{V}$ therefore coincides with $\tilde{\zeta }=0$, i.e., with the midsurface $\tilde{S}$at leading order, which is the order to which the shell theory is valid. However, $\left(h^{+}-h^{-}\right)/2=-\lambda_{s}^{0}h^{2}/8+O(\varepsilon )=O(1)$, from Eq. (36), and so *S*, defined by *ζ* = 0, is not the middle surface *ζ* = (*h*^+^ −*h*^−^)/2 of the undeformed configuration *𝒱*. We could have derived a shell theory with similar properties to the one obtained here but in which the midsurfaces correspond to the middle surface of *𝒱* but not to that of $\tilde{V}$. Since the middle surface of $\tilde{V}$ is the one that is ultimately observed, our choice is perhaps the more natural one, and we are justified in referring to 𝒮, $\tilde{S}$, 𝒮^0^ as midsurfaces.

### C Limit of small bending deformations

We conclude our calculations by taking the limit *η* → 0, in which the bending deformations become small compared to the thickness of the shell. The energy density in Eq. (63) then limits to the form familiar from classical shell theories [15],

(67) $${\hat{e}}_{0}=2C\varepsilon^{3}\;[h(E_{s}^{2}+E_{s}E_{\phi }+E_{\phi }^{2})+\frac{h^{3}}{12}\;(K_{s}^{2}+K_{s}K_{\phi }+K_{\phi }^{2})],$$

up to corrections of order *O*(*ε*^4^). This is the energy density of a thin Hookean shell [15,25,26] with Poisson’s ratio *ν* = 1/2, implying incompressibility, and elastic modulus *E* = 3*C*. In particular, our analysis also provides a formal derivation of the morphoelastic version of this classical shell theory. Again, the energy density separates into stretching and bending terms,

(68) $${\hat{e}}_{0}={\hat{e}}_{0,\text{stretch}\;}+{\hat{e}}_{0,\text{bend}},$$

with

(69a) $${\hat{e}}_{0,\;\text{stretch}\;}=\frac{1}{2}(4Ch)\varepsilon^{3}\left[E_{s}^{2}+E_{s}E_{\phi }+E_{\phi }^{2}\right],\;$$

(69b) $${\hat{e}}_{\text{0},\;\text{stretch}\;}=\frac{1}{2}\left(\frac{Ch^{3}}{3}\right)\varepsilon^{3}\left[K_{s}^{2}+K_{s}K_{\phi }+K_{\phi }^{2}\right]$$

but there is no term that couples the strains and curvature strains. Such coupling terms do arise in the expansion corresponding to Eq. (53b), but are odd functions of *Z*^0^, so disappear on integration over [−*H*^0^, *H*^0^] and hence from Eq. (67).

In this classical theory, the same stretching modulus *E*(*εh*)/(1 − *ν*^2^) = 4*C*(*εh*) and the same bending modulus *E*(*εh*)^3^/[12(1 − *ν*^2^)] = *C*(*εh*)^3^/3 are associated with all directions of stretching or bending; to pick up on a point made earlier, it is this isotropy resulting from the constitutively assumed isotropy of the material that is broken by the geometry of large bending deformations.

Of course, Eq. (67) could be derived directly by imposing different scalings, of small intrinsic bending, replacing those for large bending deformations in Eqs. (30); these scalings would considerably simplify the solutions of Eqs. (39), (40), and (44). Indeed, the structure of these calculations would be broadly similar to the earlier asymptotic derivation of the classical shell theories in Ref. [16]. We emphasize that, in either derivation, the terms at order *O*(*ε*^2^) in the expansion (46) of the deformation gradient need not be computed explicitly.

#### 1 Stretching and bending energies for small and large bending

We compare the stretching and bending energies in the small and large bending limits by observing that

(70a) $${\hat{e}}_{\text{stretch}\;}={\hat{e}}_{\text{0},\;\text{stretch}\;}+\frac{\eta^{2}\left(2-\eta^{2}\right)}{{\left(1-\eta^{2}\right)}^{2}}{\left(2E_{s}+E_{\phi }\right)}^{2},$$

(70b) $${\hat{e}}_{\text{bend}\;}={\hat{e}}_{\text{0},\;\text{bend}\;}+\frac{\eta^{2}\left(3-2\eta^{2}\right)}{36{\left(1-\eta^{2}\right)}^{2}}\left(3K_{s}+K_{\phi }\right)\left(k(\eta )K_{s}+K_{\phi }\right),\;$$

where we have used Eqs. (58) and (64) and defined

(71) $$k(\eta )=-\frac{\eta \left(4\eta^{6}-11\eta^{4}+10\eta^{2}-6\right)+6{\left(1-\eta^{2}\right)}^{2}{\text{tanh}}^{-1}\;\eta }{\eta^{5}\left(3-2\eta^{2}\right)}.$$

This shows that the classical theory underestimates the stretching energy of large bending deformations: *ê*_stretch_ ⩾ *ê*_0,stretch_ from Eq. (70a). Moreover, *ê*_stretch_ diverges as |*η*| → 1 unless the deformations are such that *E*_*ϕ*_ = −2*E*_*s*_.

The classical theory may, however, overestimate the bending energy of large bending deformations. Indeed, numerically, we find 13/5 = *k*(0) *< k*(*η*) *< k*(±1) = 3 for |*η*| < 1, and hence, from Eq. (70b), *ê*_bend_ *< ê*_0,bend_ if and only if *K*_*s*_*K*_*ϕ*_ < 0 and *k*(*η*) |*K*_*s*_| < |*K*_*ϕ*_| < 3 |*K*_*s*_|. Also from Eq. (70b), *ê*_bend_ diverges as |*η*| →1 unless *K*_*ϕ*_ = −3*K*_*s*_.

In particular, *ê*_stretch_ and *ê*_bend_ are both bounded as |*η*| → 1 if and only if *E*_*ϕ*_ = −2*E*_*s*_ and *K*_*ϕ*_= −3*K*_*s*_. In this case, Eq. (66b) shows that *ê*_couple_ is also bounded as |*η*| → 1. The conditions *E*_*ϕ*_ = −2*E*_*s*_, *K*_*ϕ*_ = −3*K*_*s*_ thus define the special deformations that allow the stretching, bending, and coupling energies to remain bounded as |*η*| → 1 that we mentioned earlier.

#### 2 Other elastic shell theories

The energy density in Eq. (67) has the same structure as the elastic energy densities used in the models referenced in the Introduction, but the morphoelastic definitions of the shell and curvature strains in Eqs. (60) and (62) differ from those in these previous models: In models not based on morphoelasticity and its multiplicative decomposition of the deformation gradient [7,8,11–13], the shell and curvature strains are simply differences of stretches or curvatures, missing the scaling factors of $f_{s}^{0},f_{\phi }^{0}$ that appear in Eqs. (60) and (62). We also note that the expressions for the curvature strains in Eqs. (62) differ by a factor of $g^{0}=f_{s}^{0}f_{\phi }^{0}$, from those in Refs. [9,10], which, as discussed in the Introduction, used a geometric approach to derive a morphoelastic shell theory. Earlier, we noted that this factor corresponds to the stretching of the intrinsic midsurface. Moreover, since ${\tilde{h}}^{\pm }=h^{0}/2+O(\varepsilon )$ as noted above, the deformed cell sheet has thickness $\tilde{h}={\tilde{h}}^{+}+{\tilde{h}}^{-}=h^{\text{0}}+O(\varepsilon )$ Equation (36) therefore yields $h/\tilde{h}=h/h^{0}+O(\varepsilon )=g^{0}+O(\varepsilon )$. The fact that the curvature strains in Eqs. (62) decrease as *g*^0^ increases therefore expresses the fact that the shell becomes easier to bend as it thins as a result of this stretching of the midsurface, with ${\hat{e}}_{\text{bend}\;},{\hat{e}}_{0,\;\text{bend}\;}\propto g_{0}^{-2}$. This geometric role of the factor *g*^0^ has been noticed previously in the context of uniform growth of an elastic shell [34].

The geometric approach in Refs. [9,10] also leads to additional terms in the energy density. The present analysis proves that these terms are not leading-order terms in the thin shell limit. However, there is no reason to expect this geometric approach to yield all terms at next order in the asymptotics. A complete expansion could in principle be obtained by continuing the asymptotic analysis presented here. Taking the analysis to higher orders in this way would in particular answer the question: at what order does the Kirchhoff hypothesis break down, i.e., at what order do the normals to the deformed midsurface diverge from those to the undeformed midsurface? This would permit asymptotic justification of the so-called shear deformation theories [35] in which the normals to the undeformed midsurface need not remain normals in the deformed configuration, but we do not pursue this further here.

## III INVAGINATION IN *VOLVOX*

### A Biological background

**Fig. 4:**
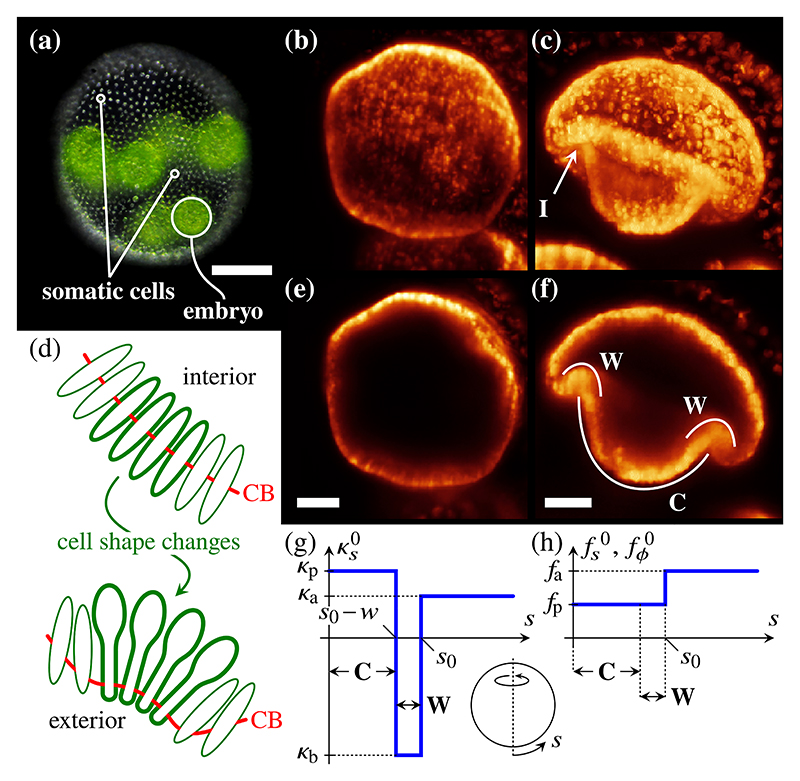
Invagination in *Volvox*. (a) *Volvox* colony, with somatic cells and one embryo labeled. (b) Light-sheet microscopy image of a spherical *Volvox* embryo before inversion. (c) Corresponding image at an early stage of inversion, when a circular invagination (I) has formed. (d) Splaying of cells and bending of the cell sheet result from the formation of wedge-shaped cells and the rearrangement of the cytoplasmic bridges (CBs); red lines indicate position of CBs. (e) Midsagittal cross section of a *Volvox* embryo before inversion. (f) Corresponding cross section during invagination, with the regions where wedge-shaped cells (W) and contracted spindle-shaped cells (C) have formed labeled. (g) Plot of the intrinsic curvature $\kappa_{s}^{0}$ against arclength *s*, defined in the inset. The plot defines the model parameters *κ*_p_, *κ*_b_, *κ*_a_, *s*_0_, and *w*. Regions of cell shape changes (W, C) as in (f) are also indicated. (h) Corresponding plot of the intrinsic Stretches $f_{s}^{0},f_{\phi }^{0}$, defining additional model parameters *f*_p_, *f*_a_. Panels (a)–(f) include microscopy images by Stephanie Höhn and have been redrawn from Ref. [8]. Scale bars: (a) 50 *μ*m; (e), (f) 20 *μ*m.

The green algal genus *Volvox* [36] has become a model for the study of the evolution of multicellularity [37,38], for biological fluid dynamics [39], and for problems in developmental biology [40,41]. Adult *Volvox* colonies [Fig. 4(a)] are spheroidal, consisting of several thousand biflagellated somatic cells that enclose a small number of germ cells [36]. Each germ cell undergoes several rounds of cell division to form a spherical embryonic cell sheet [Figs. 4(b) and 4(e)], at which stage those cell poles whence will emanate the flagella point into the sphere [36]. To acquire motility, the embryo turns itself inside out in a process called inversion [27,42].

In some species of *Volvox* [27,28], inversion starts with the formation of a circular invagination [Figs. 4(c) and 4(f)], reminiscent of the cell sheet folds associated with processes such as gastrulation or neurulation in higher organisms. At the cell level, this invagination results from two types of cell shape changes [7,28]: (1) cells near the equator become wedge-shaped [Fig. 4(d)], while the cytoplasmic bridges (cell-cell connections resulting from incomplete division) rearrange to connect the cells at their thin wedge ends, and (2) cells in the posterior hemisphere narrow in the meridional direction. These cell shape changes arise simultaneously, with (1) splaying the cells and thereby bending the cell sheet [Fig. 4(d)] and (2) contracting the posterior hemisphere to facilitate the subsequent inversion of the posterior hemisphere inside the as yet uninverted anterior hemisphere.

At later stages of inversion, other cell shape changes arise in different parts of the cell sheet [9,28] to ease the peeling of the anterior hemisphere over the inverted posterior and thus complete inversion. In particular, the anterior hemisphere of the cell sheet thins as cells there stretch anisotropically [9,28].

### B Results

Following our earlier work [7–10], we model *Volvox* inversion by considering the deformations of an incompressible elastic spherical shell under quasistatic axisymmetric variations of its intrinsic stretches and curvatures representing the cell shape changes driving inversion. The slow speed of inversion—it takes about an hour for a *Volvox* embryo to turn itself inside out [27,28]—justifies this quasistatic approximation. In more detail, Figs. 4(g) and 4(h) show functional forms of the intrinsic stretches and curvatures encoding the cell shape changes driving invagination and define the model parameters *κ*_p_, *κ*_b_, *κ*_a_, *f*_p_, *f*_a_, *s*_0_, and *w* that encode the intrinsic curvatures and intrinsic stretches of different regions of the cell sheet and the extent of these regions. In numerical calculations, we regularize the step discontinuities in the definitions of the intrinsic stretches and curvatures in Figs. 4(g) and 4(h), we nondimensionalize all lengths with the preinversion radius *R* of the embryo, and we take *εh* = 0.15, appropriate for *Volvox globator* [7,9].

We solve the governing equations derived in Appendix B numerically using the boundary value problem solver bvp4c of MATLAB (The MathWorks, Inc.) and the continuation software AUTO [43].

**Fig. 5:**
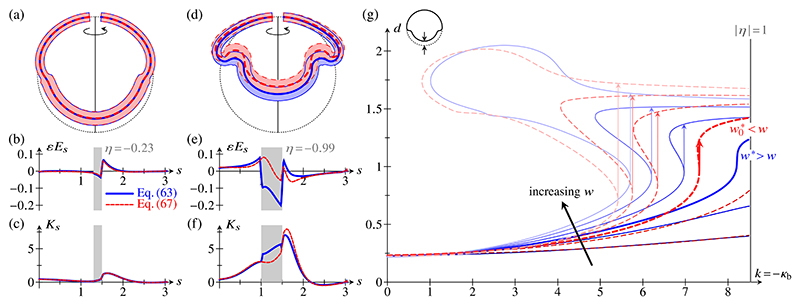
Comparison of the elastic model for large bending deformations and the classical model. Solid lines: Large bending model with energy density given by Eq. (63); dashed lines: classical model with energy density given by Eq. (67). (a) Early invagination stage: the two models yield very similar shapes. Thick lines: Midline of the cell sheet. Thin lines and shaded area: Transverse extent of the shell, illustrating the thickness variations resulting from the cell shape changes. Dotted line: midline of the undeformed spherical shell. Parameter values: *κ*_p_ = *κ*_a_ = 1, *κ*_b_ = −2, *f*_p_ = 0.8, *f*_a_ = 1, *s*_0_ =1.5, *w* = 0.2. (b) Corresponding plot of the meridional shell strain *E*_*s*_. The grey shaded area marks the bend region *s*_0_ − *w* < *s* < *s*_0_. (c) Corresponding plot of the meridional curvature strain *K*_*s*_. (d) Later invagination stage: As the cells in the bend region approach the constriction limit, the shapes resulting from the two models differ increasingly. Parameter values are as in (a), except *κ*_b_ = −8.5, *w* = 0.5. (e) Corresponding plot of the meridional shell strain *E*_*s*_. (f) Corresponding plot of the meridional curvature strain *K*_*s*_. (g) Bifurcation diagram, for different values of *w*, in (*k, d*) space, where *k* = −*κ*_b_ and *d* is the posterior displacement defined in the axis inset. Different lines correspond to parameter values *w* = 0.3, 0.5, 0.6, 0.7, 0.8, 0.9. Other parameter values are as in (a). The vertical line |*η*| = 1 corresponding to the constriction limit is also shown. For *w* > w* (in the large bending model) or $w>w_{0}^{*}$ (in the classical model), discontinuous jumps in *d*, denoted by vertical arrows, arise as *k* is increased. The thick lines correspond to *w* = 0.6 and show that $w^{*}>w_{0}^{*}$.

During the invagination stage, the radius of curvature in the bend region of wedge-shaped cells [Fig. 4(f)] becomes comparable to the thickness of the cell sheet: This is the scaling limit of large bending deformations studied in Sec. II. We therefore compare the resulting elastic model, with energy density (63), to the classical theory, in which the energy density is given by Eq. (67). For weakly invaginated stages of *Volvox* inversion (corresponding to small values of *η* in the large bending theory), the two models yield, unsurprisingly, very similar shapes [Fig. 5(a)], mirrored by very similar profiles of meridional shell strain [Fig. 5(b)] and meridional curvature strain [Fig. 5(c)]. The contraction of the posterior hemisphere leads to thickening of the cell sheet there [Fig. 5(a)]. However, the more the intrinsic configuration of the cell sheet approaches the limit of cell constriction, the more the shapes resulting from the two models differ [Fig. 5(d)]. Correspondingly, the meridional shell strain [Fig. 5(e)] and meridional curvature strain [Fig. 5(f)] in the two models differ increasingly. It may seem counterintuitive that these strains are larger in the bend region of nearly constricted cells for the large-bending model than for the classical model [Figs. 5(e) and 5(f)], since the stretching and bending cost of these larger strains is much higher in the large-bending model than in the classical model. Indeed, on computing the stretching and bending energies (not shown) of the shapes in Fig. 5(d), we find them to be much larger in the large-bending model than in the classical model. However, these large energies are balanced by a correspondingly large and negative coupling energy: for example, *E*_*s*_ < 0 and *K*_*s*_ > 0 in the bend region [Figs. 5(e) and 5(f)], while $\eta <0\;\Rightarrow {\bar{\beta }}_{ss}>0$ [Fig. 3(b)], and so ${\bar{\beta }}_{ss}E_{s}K_{s}<0$. This negative coupling energy therefore explains the large strains in the bend region that arise in the large-bending model.

The largest curvature strains [Fig. 5(f)] arise, however, in the anterior fold, i.e., in the second bend region that arises as a passive mechanical consequence of the wedge-shaped cells in the bend region just next to it [7,9]. As a result of the contraction of the posterior hemisphere, the cell sheet is thinner in the anterior [Fig. 5(d)], and hence is easier to bend there, as discussed earlier. In fact, around the invagination stage in Fig. 5(d), cells in the anterior fold begin to stretch in the meridional direction [9,28], leading to further thinning and increased bendability of the cell sheet there.

The examples in Figs. 5(a) and 5(d) indicate that the results of the two models differ at a quantitative, if not at a qualitative level. We extend this observation by plotting, for both models, *k* = −*κ*_b_ against the displacement *d* of the posterior pole [Fig. 5(g), inset] for different values of the width *w* of the bend region in Fig. 5(g). Again, the solution curves show similar behavior in the two models, but differ at a quantitative level. They confirm what one observes in Fig. 5(d), that the cell sheet is more invaginated, at the same parameter values and for sufficiently large *k*, in the classical model than in the large-bending model. Nonetheless, the cell sheet invaginates completely even in the large-bending model as *w* increases [Fig. 5(g)], i.e., as more cells become wedge-shaped and the bend region widens, as observed during *Volvox* inversion [28]. Moreover, one can argue that invagination is actually more stable in the large-bending model: There is a critical bend region width, w* in the large-bending model and $w_{0}^{*}$ in the classical model, such that the solution curves in the (*k, d*) diagram are single-valued for *w* < w_*_ or $w<w_{0}^{*}$, but become multivalued for *w* > w_*_ or $w<w_{0}^{*}$, respectively, leading to discontinuous jumps in *d* as *k* is varied. Where multiple solutions exist for a given value of *k*, the one with the lowest value of *d* has the lowest energy (not shown). For the classical theory, we have discussed this bifurcation behavior in Ref. [8], and rationalized it by constructing an effective energy that estimates different elastic contributions. It is therefore not surprising that, here, we find qualitatively identical bifurcation behavior in the two models, but that again, there are quantitative differences in the bifurcation behavior. However, Fig. 5(g) shows that $w*>w_{0}^{*}$. In other words, continuous invagination is possible in a larger region of parameter space in the large bending theory than in the classical theory: in this sense, invagination is stabilized in the large-bending theory.

This discussion shows how the geometry of large bending deformations modifies the mechanical picture of invagination suggested by the classical theory. When we introduced the problem of large bending deformations, we argued that classical shell theories cannot describe these deformations because of the assumption of large radii of curvature inherent in them. At this stage, we must therefore ask: can the large-bending theory derived here provide a complete description of the mechanics of invagination? This is first a question of self-consistency: Is the intrinsic configuration not too incompatible? In other words, are the deformations resulting from the imposed intrinsic stretches and curvatures consistent with the scalings (31) and (32) assumed in the derivation of the shell theory? Even for the late invagination stage in Fig. 5(d), the meridional shell strain remains small [Fig. 5(e)], although the meridional curvature strain reaches values of order *O*(1/*ε*) [Fig. 5(f)]. Of course, the invagination stage in Fig. 5(d) does not satisfy the restriction $1-|\eta |\gg \sqrt{\varepsilon }$ of our shell theory discussed earlier. This kind of condition is particularly restrictive for biological tissues in which *ε* is not “that small” (Fig. 1). While results remain qualitatively unchanged for somewhat smaller values of |*η*| within that range of validity, this hints that understanding the elasticity of the constriction limit |*η*| → 1 remains a key open problem for future work.

## VI Conclusion

In this paper, we have derived a morphoelastic shell theory valid for the large bending deformations that are commonly observed in developmental biology (Fig. 1), and have shown how this scaling limit of large bending deformations induces a purely geometric effective material anisotropy absent from classical shell theories. Taking the invagination of the green alga *Volvox* as an example, we have compared this large-bending theory to a simpler, classical theory not formally valid for large bending deformations. Since the classical theory does not account for the geometric material anisotropy or the singularity of cell constriction, it differs, for strongly invaginated shapes as in Figs. 1(b), 4(c), and 4(f), from the theory for large bending deformation at a quantitative, if not at a qualitative level. In particular, we have argued that these geometric effects stabilize *Volvox* invagination.

This and the growing interest in quantitative rather than merely qualitative analyses of morphogenesis [44,45] emphasize the importance of this scaling limit of large bending deformations for studies of the mechanics of developmental biology. The theory we have derived here is not, however, the most general theory of these large bending deformations. Indeed, when writing down the expression for the intrinsic deformation gradient in Eq. (24), we assumed that there is no intrinsic displacement parallel to the midsurface, *𝜍*^0^ = 0. The nonlinear differential equations extending Eqs. (39) and (40) that arise in the expansions of the boundary and incompressibility conditions for *𝜍*^0^ ≠ 0 still admit a trivial solution *p*_(0)_ = 1, *Z*_(0)_ ≡ *Z*^0^, *S*_(0)_ ≡ *S*^0^, where $S^{0}=f_{s}^{0}f_{\phi }^{0}{𝜍}^{0}$.We were, however, unable to extend our calculations in Sec. II to prove that this solution is unique; a similar issues arises when extending the calculations of this paper to more general constitutive relations, as discussed below and in Appendix C. It therefore remains unclear what form the extension of the Kirchhoff “hypothesis” [15] to this case takes.

In this paper, we assumed the simplest, incompressible neo-Hookean constitutive relations when deriving our shell theory for large bending deformations. The restriction to incompressible elastic materials is justified by the biological context of our analysis, in which the models derived here describe sheets of fluid-filled cells that are therefore indeed incompressible to a first approximation. However, the bulk elastic response of biological materials such as brain tissue is not linear [46–48]. The restriction to linear neo-Hookean relations may therefore appear to be a limitation of the analysis, but that turns out not to be the case: in the thin shell limit, general hyperelastic constitutive relations reduce to neo-Hookean relations. This result has been established previously for thin plates [20,49], and, in Appendix C, we (partially) extend it to the large bending deformations of thin shells considered here. In the context of shell theories, the problem of specifying the nonlinear constitutive relations of biological tissues does not therefore arise. However, we have recently shown that the continuum limit of a class of discrete models of cell sheets involves not only nonlinear elastic, but also non-local, nonelastic terms [50]. Moreover, adding the geometric singularity of apical constriction (corresponding to triangular cells in the underlying discrete model) as a constraint to the variational problem that arises in this continuum limit remains an important open problem [50]. Solving this may provide a regularization of the singularity that breaks asymptoticity as |*η*| →1 in the theory derived here, and hence a yet more complete mechanical picture of the bend region of wedge-shaped cells in *Volvox* invagination [Fig. 4(d)]. Meanwhile, all of this suggests that the journey toward understanding the continuum mechanics of biological materials, on which we have taken another step with the present analysis of large bending deformations of thin elastic shells, will continue to abound with new problems in nonlinear mechanics.

## Supplementary Material

Supplementary Materials
