# Supplementary material for: Morphoelasticity of large bending deformations of cell sheets during development
Source: Phys Rev E. Author manuscript; Available in PMC 2024 Jul 10. (PMC7616142; doi:10.1103/PhysRevE.103.022411)
Supplement: Supplementary Materials [file EMS196597-supplement-Supplementary_Materials.pdf]

## APPENDIX A: THIN-SHELL THEORY FOR LARGE BENDING DEFORMATIONS OF AN ELASTIC SHELL

In this Appendix, we extend the calculations for axisymmetric deformations of an elastic shell in Sec. II to general deformations.

### 1. Deformations of an elastic shell

As in Sec. II, we begin by deriving expressions for the deformation gradient tensors of an elastic shell of thickness $\varepsilon h$, where $\varepsilon$ is, again, a small asymptotic parameter that expresses the thinness of the shell.

#### a. Undeformed configuration of the shell

We parameterize the undeformed midsurface $\mathcal{S}$ of the shell in terms of generalized, not necessarily orthogonal coordinates; we shall use Greek letters to denote these coordinates. Thus, if $\boldsymbol{\rho}$ is the position of a point on $\mathcal{S}$, the tangent vectors there are $\boldsymbol{e_\alpha} = \partial\boldsymbol{\rho}/\partial\alpha$. The metric $\mathsf{g}$ of the midsurface thus has components $g_{\alpha\beta} = \boldsymbol{e_\alpha}\cdot\boldsymbol{e_\beta}$, and we set $g = \det\mathsf{g}$.

Next, we define a basis $\mathcal{B}$ for the shell by adjoining the unit normal vector $\boldsymbol{n}$ to this tangent basis. This obeys the Weingarten equation [51]

$$\boldsymbol{n}_{,\alpha} = -\varkappa_\alpha{}^\beta \boldsymbol{e_\beta}, \tag{A1}$$

in which commata denote partial differentiation and the (symmetric) curvature tensor is $\varkappa_{\alpha\beta} = -\boldsymbol{e_\alpha}\cdot\boldsymbol{n}_{,\beta}$.

The position of a point in the undeformed configuration $\mathcal{V}$ of the shell is $\boldsymbol{r} = \boldsymbol{\rho} + \varepsilon\zeta\boldsymbol{n}$, where $\zeta$ denotes the transverse coordinate, as defined for axisymmetric deformations in Fig. 2(c). Hence

$$\boldsymbol{r}_{,\alpha} = \left(\delta_\alpha{}^\beta - \varepsilon\zeta\varkappa_\alpha{}^\beta\right)\boldsymbol{e_\beta}, \quad \boldsymbol{r}_{,\zeta} = \varepsilon\boldsymbol{n}, \tag{A2}$$

wherein we have used the Weingarten equation (A1), and where $\delta$ is the Kronecker delta. The metric $\mathsf{G}$ of the undeformed configuration therefore has components

$$G_{\zeta\zeta} = \varepsilon^2, \quad G_{\alpha\zeta} = G_{\zeta\alpha} = 0, \tag{A3a}$$

and

$$G_{\alpha\beta} = g_{\alpha\gamma}(\delta^\gamma{}_\delta - \varepsilon\zeta\varkappa^\gamma{}_\delta)(\delta^\delta{}_\beta - \varepsilon\zeta\varkappa^\delta{}_\beta), \tag{A3b}$$

where we have used the symmetry of the curvature tensor. In particular, its inverse has components

$$G^{\zeta\zeta} = \varepsilon^{-2}, \quad G^{\alpha\zeta} = G^{\zeta\alpha} = 0, \quad G^{\alpha\beta}. \tag{A4}$$

The position vectors of the surfaces $\zeta = \pm h^\pm$ of the undeformed shell are $\boldsymbol{r}^\pm = \boldsymbol{\rho} \pm \varepsilon h^\pm\boldsymbol{n}$, and hence the tangent vectors to these surfaces are

$$\boldsymbol{e}^\pm_{\boldsymbol{\alpha}} = \boldsymbol{r}^\pm{}_{,\alpha} = \left(\delta_\alpha{}^\beta \mp \varepsilon h^\pm\varkappa_\alpha{}^\beta\right)\boldsymbol{e_\beta} \pm \varepsilon h^\pm{}_{,\alpha}\boldsymbol{n}. \tag{A5}$$

We now order $\mathcal{B} = \{\boldsymbol{e_1}, \boldsymbol{e_2}, \boldsymbol{n}\}$ as a right-handed basis by exchanging $\boldsymbol{n} \leftrightarrow -\boldsymbol{n}$ if required. Expanding in components, this implies that $\boldsymbol{e_1}\times\boldsymbol{e_2} = \sqrt{g}\boldsymbol{n}$, and hence $\boldsymbol{e_1}\times\boldsymbol{n} = -\boldsymbol{e}^2/\sqrt{g}$, $\boldsymbol{e_2}\times\boldsymbol{n} = \boldsymbol{e}^1/\sqrt{g}$. Continuing to expand in components and after some calculations, we infer

$$\begin{aligned}\boldsymbol{e}^\pm_{\boldsymbol{1}}\times\boldsymbol{e}^\pm_{\boldsymbol{2}} &= [1 \mp 2\varepsilon h^\pm H + \varepsilon^2(h^\pm)^2K]\sqrt{g}\boldsymbol{n}\\ &\quad \mp \varepsilon h^\pm{}_{,\alpha}\left[(1 \pm \varepsilon h^\pm H)\delta^\alpha{}_\beta \mp \varepsilon\varkappa_\beta{}^\alpha h^\pm\right]\frac{\boldsymbol{e^\beta}}{\sqrt{g}},\end{aligned} \tag{A6}$$

wherein we have identified $H = \frac{1}{2}\varkappa_\alpha{}^\alpha$ and $K = \det\varkappa_\alpha{}^\beta$ as the mean and Gaussian curvatures [51] of $\mathcal{S}$. On normalizing these vectors, we obtain the normals to the shell surfaces,

$$\boldsymbol{n}^\pm = \frac{\boldsymbol{n} \mp \nu^\pm{}_\alpha\boldsymbol{e^\alpha}}{\sqrt{1 + \nu^\pm{}_\beta\nu^{\pm\beta}}}, \tag{A7a}$$

with

$$\nu^\pm{}_\alpha = \frac{\varepsilon h^\pm{}_{,\beta}[(1 \pm \varepsilon h^\pm H)\delta^\beta{}_\alpha \mp \varepsilon\varkappa_\alpha{}^\beta h^\pm]}{g[1 \mp 2\varepsilon h^\pm H + \varepsilon^2(h^\pm)^2K]}. \tag{A7b}$$

#### b. Deformed configuration of the shell

We take the same generalized coordinates to parameterize the deformed midsurface $\tilde{\mathcal{S}}$ of the shell. The tangent vectors at a point $\tilde{\boldsymbol{\rho}}$ on $\tilde{\mathcal{S}}$ are thus $\tilde{\boldsymbol{e}}_{\boldsymbol{\alpha}} = \partial\tilde{\boldsymbol{\rho}}/\partial\alpha$. The metric $\tilde{\mathsf{g}}$ of the midsurface has components $\tilde{g}_{\alpha\beta} = \tilde{\boldsymbol{e}}_{\boldsymbol{\alpha}}\cdot\tilde{\boldsymbol{e}}_{\boldsymbol{\beta}}$, and we let $\tilde{g} = \det\tilde{\mathsf{g}}$. We extend the tangent basis of $\tilde{\mathcal{S}}$ to a basis $\tilde{\mathcal{B}}$ for the deformed shell by adding the unit normal $\tilde{\boldsymbol{n}}$, and introduce the (symmetric) curvature tensor $\tilde{\kappa}_{\alpha\beta} = -\tilde{\boldsymbol{e}}_{\boldsymbol{\alpha}}\cdot\tilde{\boldsymbol{n}}_{,\beta}$. The Weingarten and Gauß equations [51]

$$\tilde{\boldsymbol{n}}_{,\alpha} = -\tilde{\kappa}_\alpha{}^\beta\tilde{\boldsymbol{e}}_{\boldsymbol{\beta}}, \quad \tilde{\boldsymbol{e}}_{\boldsymbol{\alpha},\beta} = \tilde{\kappa}_{\alpha\beta}\tilde{\boldsymbol{n}} + \tilde{\Gamma}_{\alpha\beta}{}^\gamma\tilde{\boldsymbol{e}}_{\boldsymbol{\gamma}} \tag{A8}$$

express the derivatives of the normal and tangent vectors in terms of the curvature tensor and Christoffel symbols associated with the deformed midsurface metric [51]. The position of a point in the deformed configuration $\tilde{\mathcal{V}}$ of the shell is

$$\tilde{\boldsymbol{r}} = \tilde{\boldsymbol{\rho}} + \varepsilon(\tilde{\zeta}\tilde{\boldsymbol{n}} + \tilde{\xi}^\alpha\tilde{\boldsymbol{e}}_{\boldsymbol{\alpha}}), \tag{A9}$$

where $\tilde{\zeta}$ and $\tilde{\xi}^\alpha$ are the transverse and parallel displacements of this point relative to the midsurface, defined for axisymmetric deformations in Fig. 2(e). In particular, the displacement parallel to the midsurface is now no longer a scalar. Using the Weingarten and Gauß equations (A8), we find

$$\tilde{\boldsymbol{r}}_{,\alpha} = \left[\delta_\alpha{}^\beta + \varepsilon\left(\tilde{\xi}^\beta{}_{;\alpha} - \tilde{\zeta}\tilde{\kappa}_\alpha{}^\beta\right)\right]\tilde{\boldsymbol{e}}_{\boldsymbol{\beta}} + \varepsilon(\tilde{\zeta}_{,\alpha} + \tilde{\xi}^\beta\tilde{\kappa}_{\alpha\beta})\tilde{\boldsymbol{n}}, \tag{A10a}$$

$$\tilde{\boldsymbol{r}}_{,\zeta} = \varepsilon\left(\tilde{\zeta}_{,\zeta}\tilde{\boldsymbol{n}} + \tilde{\xi}^\alpha{}_{,\zeta}\tilde{\boldsymbol{e}}_{\boldsymbol{\alpha}}\right), \tag{A10b}$$

in which $\tilde{\xi}^\beta{}_{;\alpha} = \tilde{\xi}^\beta{}_{,\alpha} + \tilde{\Gamma}_{\alpha\gamma}{}^\beta\tilde{\xi}^\gamma$ is a covariant derivative. It follows that the metric $\tilde{\mathsf{G}}$ of $\tilde{\mathcal{V}}$ has components

$$\tilde{G}_{\zeta\zeta} = \varepsilon^2[(\tilde{\zeta}_{,\zeta})^2 + \tilde{\xi}^\alpha{}_{,\zeta}\tilde{\xi}_{\alpha,\zeta}], \tag{A11a}$$

$$\begin{aligned}\tilde{G}_{\alpha\zeta} = \tilde{G}_{\zeta\alpha} &= \varepsilon\tilde{\xi}_{\alpha,\zeta} + \varepsilon^2\left[\tilde{\zeta}_{,\zeta}(\tilde{\zeta}_{,\alpha} + \tilde{\xi}^\beta\tilde{\kappa}_{\alpha\beta})\right.\\ &\quad \left.+ \tilde{\xi}_{\beta,\zeta}\left(\tilde{\xi}^\beta{}_{;\alpha} - \tilde{\zeta}\tilde{\kappa}_\alpha{}^\beta\right)\right],\end{aligned} \tag{A11b}$$

$$\begin{aligned}\tilde{G}_{\alpha\beta} &= \tilde{g}_{\alpha\gamma}\left[\delta^\gamma{}_\delta + \varepsilon\left(\tilde{\xi}_\delta{}^{;\gamma} - \tilde{\zeta}\tilde{\kappa}^\gamma{}_\delta\right)\right]\left[\delta_\beta{}^\delta + \varepsilon\left(\tilde{\xi}^\delta{}_{;\beta} - \tilde{\zeta}\tilde{\kappa}_\beta{}^\delta\right)\right]\\ &\quad + \varepsilon^2(\tilde{\zeta}_{,\alpha} + \tilde{\xi}^\gamma\tilde{\kappa}_{\alpha\gamma})(\tilde{\zeta}_{,\beta} + \tilde{\xi}^\delta\tilde{\kappa}_{\beta\delta}).\end{aligned} \tag{A11c}$$

#### c. Intrinsic configuration of the shell: Incompatibility

We define the intrinsic configuration of the shell by specifying the symmetric positive-definite intrinsic metric

components ${g^0}_{\alpha\beta}$, the symmetric intrinsic curvatures ${\kappa^0}_{\alpha\beta}$, and the intrinsic transverse displacement $\zeta^0$, which is an increasing function of $\zeta$. It follows from a local embedding theorem for Riemannian metrics [30,31] that the surface $\mathcal{S}^0$ with metric components ${g^0}_{\alpha\beta}$ can be embedded into three-dimensional Euclidean space, and we denote by $\mathcal{B}^0$ the corresponding intrinsic basis containing the tangent vectors $\boldsymbol{E_\alpha}$ and the normal $\boldsymbol{N}$ such that ${g^0}_{\alpha\beta} = \boldsymbol{E_\alpha}\cdot\boldsymbol{E_\beta}$.

The components of the curvature tensor ${\varkappa^0}_{\alpha\beta} = -\boldsymbol{E_\alpha}\cdot\boldsymbol{N}_{,\beta}$ associated with $\mathcal{S}^0$ are in general different from the intrinsic curvatures ${\kappa^0}_{\alpha\beta}$, since the latter are specified independently from the definition of $\mathcal{S}^0$. This expresses the incompatibility of the intrinsic metric $\mathsf{G}^0$ of the intrinsic configuration $\mathcal{V}^0$ of the shell. This metric has components

$$ {G^0}_{\zeta\zeta} = \varepsilon^2\big({\zeta^0}_{,\zeta}\big)^2,\quad {G^0}_{\alpha\zeta} = {G^0}_{\zeta\alpha} = \varepsilon^2{\zeta^0}_{,\zeta}{\zeta^0}_{,\alpha}, \tag{A12a}$$

and

$$ {G^0}_{\alpha\beta} = {g^0}_{\alpha\gamma}\big({\delta^\gamma}_\delta - \varepsilon\zeta^0{\kappa^0}^\gamma{}_\delta\big)\big({\delta^\delta}_\beta - \varepsilon\zeta^0{\kappa^0}_\beta{}^\delta\big) + \varepsilon^2{\zeta^0}_{,\alpha}{\zeta^0}_{,\beta}, \tag{A12b}$$

that we write down by analogy with Eqs. (A11), assuming, as we did in Sec. II, that there is no intrinsic displacement parallel to the midsurface, $\varsigma^{0\alpha} = 0$. We emphasize again that, in contrast with the intrinsic metric components ${g^0}_{\alpha\beta}$, the intrinsic curvatures ${\kappa^0}_{\alpha\beta}$ and the intrinsic transverse displacement $\zeta^0$ remain without a direct geometric realization.

As in Sec. II, we specify $\zeta^0$ by imposing intrinsic volume conservation. The condition of intrinsic volume conservation reads $\sqrt{\det\mathsf{G}^0} = \sqrt{\det\mathsf{G}}$, or, as we argue in what follows and equivalently, $\det\mathbf{F^0} = 1$, where the intrinsic deformation gradient $\mathbf{F^0}$ is given by Eq. (A17b) below. We shall integrate the differential equation resulting from this condition under the scaling assumptions of shell theory later, and we shall again choose the midsurfaces $\mathcal{S}$, $\tilde{\mathcal{S}}$, and $\mathcal{S}^0$ in such a way that the shell surfaces $\zeta = \pm h^\pm$ and $\tilde{\zeta} = \pm\tilde{h}^\pm$ correspond to $\zeta^0 = \pm h^0/2$. We recall that the intrinsic thickness $h^0$ also lacks a direct geometric realization.

### *d. Calculation of the deformation gradient tensors*

The geometric deformation gradient is $\tilde{\mathbf{F}} = \operatorname{Grad}\tilde{\boldsymbol{r}}$, where, by definition, $\operatorname{Grad}\tilde{\boldsymbol{r}} = \tilde{\boldsymbol{r}}_{,\alpha}\otimes\boldsymbol{r}^{,\alpha} + \tilde{\boldsymbol{r}}_{,\zeta}\otimes\boldsymbol{r}^{,\zeta}$. Now, from Eqs. (A4),

$$ \boldsymbol{r}^{,\alpha} = G^{\alpha\gamma}(g_{\gamma\beta} - \varepsilon\zeta\varkappa_{\gamma\beta})\boldsymbol{e^\beta},\quad \boldsymbol{r}^{,\zeta} = \varepsilon^{-1}\boldsymbol{n}. \tag{A13}$$

Using Eqs. (A10), it follows that

$$\begin{aligned}\tilde{\mathbf{F}} &= \big({\delta^\alpha}_\gamma - \varepsilon\tilde{\zeta}{\tilde{\kappa}^\alpha}{}_\gamma + \varepsilon{\tilde{\varsigma}^\alpha}{}_{;\gamma}\big)G^{\gamma\delta}(g_{\delta\beta} - \varepsilon\zeta\varkappa_{\delta\beta})\tilde{\boldsymbol{e}}_{\boldsymbol{\alpha}}\otimes\boldsymbol{e^\beta}\\ &\quad + \varepsilon\big(\tilde{\zeta}^{,\alpha} + {\tilde{\kappa}^\alpha}{}_\epsilon\tilde{\varsigma}^\epsilon\big)\tilde{g}_{\alpha\delta}G^{\delta\gamma}(g_{\gamma\beta} - \varepsilon\zeta\varkappa_{\gamma\beta})\tilde{\boldsymbol{n}}\otimes\boldsymbol{e^\beta}\\ &\quad + {\tilde{\varsigma}^\alpha}{}_{,\zeta}\tilde{\boldsymbol{e}}_{\boldsymbol{\alpha}}\otimes\boldsymbol{n} + \tilde{\zeta}_{,\zeta}\tilde{\boldsymbol{n}}\otimes\boldsymbol{n},\end{aligned} \tag{A14a}$$

or, in block matrix notation [52],

$$ \tilde{\mathbf{F}} = \left(\begin{array}{c|c}\tilde{\mathsf{A}}\mathsf{H} & \tilde{\varsigma}_{,\zeta}\\ \hline \tilde{\mathsf{b}}^\top\tilde{\mathsf{g}}\mathsf{H} & \tilde{\zeta}_{,\zeta}\end{array}\right)\quad[\tilde{\mathcal{B}}\otimes\mathcal{B}^*], \tag{A14b}$$

in which the asterisk denotes a dual basis, and where we have introduced

$$ {H^\alpha}_\beta = G^{\alpha\gamma}g_{\gamma\delta}{A^\delta}_\beta\quad\text{with}\quad {A^\alpha}_\beta = {\delta^\alpha}_\beta - \varepsilon\zeta{\varkappa^\alpha}_\beta, \tag{A15}$$

and where we have also let

$$ {\tilde{A}^\alpha}{}_\beta = {\delta^\alpha}_\beta - \varepsilon\tilde{\zeta}{\tilde{\kappa}^\alpha}{}_\beta + \varepsilon{\tilde{\varsigma}^\alpha}{}_{;\beta},\quad \tilde{b}^\alpha = \varepsilon\big(\tilde{\zeta}^{,\alpha} + {\tilde{\kappa}^\alpha}{}_\beta\tilde{\varsigma}^\beta\big). \tag{A16}$$

By analogy with Eqs. (A14), the intrinsic deformation gradient tensor is

$$\begin{aligned}\mathbf{F^0} &= \big({\delta^\alpha}_\gamma - \varepsilon\zeta^0{\kappa^{0\alpha}}_\gamma\big)G^{\gamma\delta}(g_{\delta\beta} - \varepsilon\zeta\varkappa_{\delta\beta})\boldsymbol{E_\alpha}\otimes\boldsymbol{e^\beta}\\ &\quad + \varepsilon\zeta^{0,\alpha}{g^0}_{\alpha\delta}G^{\delta\gamma}(g_{\gamma\beta} - \varepsilon\zeta\varkappa_{\gamma\beta})\boldsymbol{N}\otimes\boldsymbol{e^\beta} + {\zeta^0}_{,\zeta}\boldsymbol{N}\otimes\boldsymbol{n},\end{aligned} \tag{A17a}$$

or, in block matrix notation,

$$ \mathbf{F^0} = \left(\begin{array}{c|c}\mathsf{A}^0\mathsf{H} & 0\\ \hline {\mathsf{b}^0}^\top\mathsf{g}^0\mathsf{H} & {\zeta^0}_{,\zeta}\end{array}\right)\quad[\mathcal{B}^0\otimes\mathcal{B}^*]. \tag{A17b}$$

Here we have again assumed that there is no intrinsic displacement parallel to the midsurface, $\varsigma^{0\alpha} = 0$, and we have introduced

$$ {A^{0\alpha}}_\beta = {\delta^\alpha}_\beta - \varepsilon\zeta^0{\kappa^{0\alpha}}_\beta,\quad b^{0\alpha} = \varepsilon\zeta^{0,\alpha}. \tag{A18}$$

At this stage, we interrupt the computation of the deformation gradient tensors and we discuss the condition of intrinsic volume conservation. From Eq. (A3b) and definition (A15), $G_{\alpha\beta} = g_{\alpha\gamma}{A^\gamma}_\delta{A^\delta}_\beta$. Now $\det\mathsf{MN} = \det\mathsf{M}\det\mathsf{N}$ for matrices $\mathsf{M}$, $\mathsf{N}$, so, from Eqs. (A3a),

$$ \det\mathsf{G} = \varepsilon^2 g(\det\mathsf{A})^2, \tag{A19a}$$

where we recall the definition $g = \det\mathsf{g}$. Similarly, on introducing $g^0 = \det\mathsf{g}^0$ and on evaluating the determinant of a block matrix [53], Eqs. (A12) yield

$$ \det\mathsf{G}^0 = \varepsilon^2({\zeta^0}_{,\zeta})^2 g^0(\det\mathsf{A}^0)^2. \tag{A19b}$$

Above, we have claimed that the intrinsic volume conservation condition $\sqrt{\det\mathsf{G}^0} = \sqrt{\det\mathsf{G}}$ is equivalent with the tensorial condition $\det\mathbf{F^0} = 1$. Since Eq. (A17b) expresses the intrinsic deformation gradient with respect to a mixed non-orthogonal basis, we shall need the following observation to evaluate the determinant and hence prove our claim:

*Proposition 1.* Let $\{\boldsymbol{e_\alpha}\}$ and $\{\boldsymbol{E_\beta}\}$ be right-handed bases with corresponding metrics $g_{\alpha\beta} = \boldsymbol{e_\alpha}\cdot\boldsymbol{e_\beta}$, and $G_{\alpha\beta} = \boldsymbol{E_\alpha}\cdot\boldsymbol{E_\beta}$, and let $\mathbf{M} = {M^\alpha}_\beta\boldsymbol{e_\alpha}\otimes\boldsymbol{E^\beta}$ be a tensor represented by the matrix $\mathsf{M} = ({M^\alpha}_\beta)$ with respect to $\{\boldsymbol{e_\alpha}\}\otimes\{\boldsymbol{E^\beta}\}$. Let $g = \det g_{\alpha\beta}$ and $G = \det G_{\alpha\beta}$. Then

$$ \det\mathbf{M} = \sqrt{\frac{g}{G}}\det\mathsf{M}. $$

*Proof.* Let $\{\boldsymbol{X_i}\}$ be the standard Cartesian basis, and write $\boldsymbol{e_\alpha} = e_{\alpha i}\boldsymbol{X_i}$, $\boldsymbol{E_\alpha} = E_{\alpha i}\boldsymbol{X_i}$. Let $e = \det e_{\alpha i}$, $E = \det E_{\alpha i}$. By assumption, $e, E > 0$. By definition, $g_{\alpha\beta} = \boldsymbol{e_\alpha}\cdot\boldsymbol{e_\beta} = e_{\alpha i}e_{\beta i}$ as $\boldsymbol{X_i}\cdot\boldsymbol{X_j} = \delta_{ij}$. Since $\det e_{\beta i} = \det e_{i\beta}$, $e^2 = g$. Similarly, $E^2 = G$. Now

$$ \mathbf{M} = e_{\alpha i}{M^\alpha}_\beta G^{\beta\gamma}E_{\gamma j}\boldsymbol{X_i}\otimes\boldsymbol{X_j}, $$

which implies, since $\det\mathsf{G}^{-1} = G^{-1}$, $\det\mathbf{M} = e(\det\mathsf{M})G^{-1}E$. This completes the proof [54]. ∎

Since the normal vectors $\boldsymbol{n}$ in $\mathcal{B}$ and $\boldsymbol{N}$ in $\mathcal{B}^0$ are, by definition, unit vectors perpendicular to the remaining basis vectors, Proposition 1 and Eq. (A17b) yield

$$\det \mathbf{F^0} = \sqrt{\frac{g^0}{g}} \det \mathsf{F}^0 = \sqrt{\frac{g^0}{g}} \zeta^0{}_{,\zeta} \det \mathsf{A}^0 \det \mathsf{H}. \tag{A20a}$$

Now definition (A15) implies, since $G_{\alpha\beta} = g_{\alpha\gamma} A^\gamma{}_\delta A^\delta{}_\beta$, that

$$\det \mathsf{H} = [g(\det \mathsf{A})^2]^{-1} g \det \mathsf{A} = \frac{1}{\det \mathsf{A}}, \tag{A20b}$$

Since we assume $\zeta^0{}_{,\zeta} > 0$, Eqs. (A19) and (A20) show that $\sqrt{\det \mathsf{G}^0} = \sqrt{\det \mathsf{G}} \iff \det \mathbf{F^0} = 1$, as claimed. Because we have written down Eqs. (A12) and (A17) defining the incompatible metric of $\mathcal{V}^0$ and the intrinsic deformation gradient $\mathbf{F^0}$ by analogy with the corresponding results for the deformation configuration $\tilde{\mathcal{V}}$, but have not derived them from an embedding of $\mathcal{V}^0$, it is not *a priori* clear that these expressions are consistent. This is why we needed to show, as we did in Sec. II, that the expression for $\mathbf{G^0}$ is consistent with that for $\mathbf{F^0}$ as far the only use of the former (i.e., intrinsic volume conservation or the definition of the intrinsic volume element) is concerned. Equivalently, intrinsic volume conservation can be imposed without reference to the incompatible metric $\mathbf{G}^0$; consequently, as also noted in Sec. II, the volume element $dV^0$ of $\mathcal{V}^0$ can be also be defined with reference to $\mathbf{F^0}$ only.

We now return to the computation of the elastic deformation gradient $\mathbf{F} = \tilde{\mathbf{F}}(\mathbf{F^0})^{-1}$. On inverting the block-lower triangular matrix in Eq. (A17b), we find

$$(\mathbf{F^0})^{-1} = \left(\begin{array}{c|c} \mathsf{H}^{-1}(\mathsf{A}^0)^{-1} & 0 \\ \hline -\dfrac{\mathsf{b}^{0\top}\mathsf{g}^0(\mathsf{A}^0)^{-1}}{\zeta^0{}_{,\zeta}} & \dfrac{1}{\zeta^0{}_{,\zeta}} \end{array}\right) \quad [\mathcal{B}\otimes(\mathcal{B}^0)^*]. \tag{A21}$$

From this and from Eq. (A14b), we obtain

$$\mathbf{F} = \left(\begin{array}{c|c} (\tilde{\mathsf{A}} - \tilde{\varsigma}_{,\zeta^0}\mathsf{b}^{0\top}\mathsf{g}^0)(\mathsf{A}^0)^{-1} & \varsigma_{,\zeta^0} \\ \hline (\tilde{\mathsf{b}}^\top\tilde{\mathsf{g}} - \tilde{\zeta}_{,\zeta^0}\mathsf{b}^{0\top}\mathsf{g}^0)(\mathsf{A}^0)^{-1} & \tilde{\zeta}_{,\zeta^0} \end{array}\right) \quad [\tilde{\mathcal{B}}\otimes(\mathcal{B}^0)^*]. \tag{A22}$$

### 2. Thin shell theory for large bending deformations

As in Sec. II, we assume that the shell is made of an incompressible neo-Hookean material, with energy given by Eqs. (26). Equation (28) still provides an expression for the stress tensor $\mathbf{Q}$, now with respect to $\tilde{\mathcal{B}}\otimes(\mathcal{B}^0)^*$, and with the deformation gradients $\tilde{\mathbf{F}}, \mathbf{F^0}, \mathbf{F}$ now given by Eqs. (A14b), (A17b), and (A22), respectively. Moreover, Eq. (29b) still holds.

#### *a. Scaling assumptions*

Again as in Sec. II, we rescale the intrinsic and deformed curvature tensors, $\boldsymbol{\kappa^0} = \kappa^{0\alpha}{}_\beta \boldsymbol{E_\alpha}\otimes\boldsymbol{E^\beta}$ and $\tilde{\boldsymbol{\kappa}} = \tilde{\kappa}^\alpha{}_\beta\tilde{\boldsymbol{e}}_{\boldsymbol{\alpha}}\otimes\tilde{\boldsymbol{e}}^{\boldsymbol{\beta}}$, to introduce large bending deformations explicitly and absorb the intrinsic stretching of the midsurface by writing

$$\boldsymbol{\kappa^0} = \sqrt{\frac{g^0}{g}}\frac{\boldsymbol{\lambda^0}}{\varepsilon}, \quad \tilde{\boldsymbol{\kappa}} = \sqrt{\frac{g^0}{g}}\frac{\tilde{\boldsymbol{\lambda}}}{\varepsilon}, \tag{A23}$$

In what follows, we shall need explicit representations of these tensors, $\boldsymbol{\lambda^0} = \lambda^{0\alpha}{}_\beta\boldsymbol{E_\alpha}\otimes\boldsymbol{E^\beta}$ and $\tilde{\boldsymbol{\lambda}} = \tilde{\lambda}^\alpha{}_\beta\tilde{\boldsymbol{e}}_{\boldsymbol{\alpha}}\otimes\tilde{\boldsymbol{e}}^{\boldsymbol{\beta}}$, and shall denote by $\mathsf{\lambda}^0$ and $\tilde{\mathsf{\lambda}}$ the corresponding matrices of components.

Next, we make the standard scaling assumptions of shell theory, that the elastic strains remain small. To this end, we introduce the deformation gradient restricted to the midsurface,

$$\mathbf{f} = \tilde{\boldsymbol{e}}_{\boldsymbol{\alpha}}\otimes\boldsymbol{E^\alpha}. \tag{A24}$$

First, we require that the shell strains be small: accordingly, we define the shell strain tensor $\mathbf{E}$ by

$$2\varepsilon\mathbf{E} = \mathbf{f}^\top\mathbf{f} - \mathbf{I}. \tag{A25}$$

Now $\mathbf{f}^\top = \boldsymbol{E^\alpha}\otimes\tilde{\boldsymbol{e}}_{\boldsymbol{\alpha}}$, so $\mathbf{f}^\top\mathbf{f} = \tilde{g}_{\alpha\beta}\boldsymbol{E^\alpha}\otimes\boldsymbol{E^\beta} = g^{0\alpha\gamma}\tilde{g}_{\gamma\beta}\boldsymbol{E_\alpha}\otimes\boldsymbol{E^\beta}$. Hence, if we set $\mathbf{E} = E^\alpha{}_\beta\boldsymbol{E_\alpha}\otimes\boldsymbol{E^\beta}$, then

$$2\varepsilon E^\alpha{}_\beta = g^{0\alpha\gamma}g_{\gamma\beta} - \delta^\alpha{}_\beta \quad \text{or} \quad 2\varepsilon\mathsf{E} = (\mathsf{g}^0)^{-1}\tilde{\mathsf{g}} - \mathsf{I}, \tag{A26a}$$

in equivalent matrix notation. In the calculations that follow, we shall need a consequence of this definition,

$$\tilde{\mathsf{g}} = \mathsf{g}^0(\mathsf{I} + 2\varepsilon\mathsf{E}). \tag{A26b}$$

Second, we require that the curvature strains remain small: we therefore introduce two different (scaled) curvature strain tensors,

$$\varepsilon\mathbf{L} = \mathbf{f}^{-1}\tilde{\boldsymbol{\lambda}}\mathbf{f} - \boldsymbol{\lambda^0}, \quad \varepsilon\mathbf{K} = \mathbf{f}^\top\tilde{\boldsymbol{\lambda}}\mathbf{f} - \boldsymbol{\lambda^0}. \tag{A27}$$

Since $\mathbf{f}^{-1} = \boldsymbol{E_\alpha}\otimes\tilde{\boldsymbol{e}}^{\boldsymbol{\alpha}}$, $\mathbf{f}^{-1}\tilde{\boldsymbol{\lambda}}\mathbf{f} = \tilde{\lambda}^\alpha{}_\beta\boldsymbol{E_\alpha}\otimes\boldsymbol{E^\beta}$, and hence, on writing $\mathbf{L} = L^\alpha{}_\beta\boldsymbol{E_\alpha}\otimes\boldsymbol{E^\beta}$, we find [55]

$$\varepsilon L^\alpha{}_\beta = \tilde{\lambda}^\alpha{}_\beta - \lambda^{0\alpha}{}_\beta \quad \text{or} \quad \varepsilon\mathsf{L} = \tilde{\mathsf{\lambda}} - \mathsf{\lambda}^0. \tag{A28}$$

Similarly, $\mathbf{f}^\top\tilde{\boldsymbol{\lambda}}\mathbf{f} = g^{0\alpha\gamma}\tilde{g}_{\gamma\delta}\tilde{\lambda}^\delta{}_\beta\boldsymbol{E_\alpha}\otimes\boldsymbol{E^\beta}$, whence, on letting $\mathbf{K} = K^\alpha{}_\beta\boldsymbol{E_\alpha}\otimes\boldsymbol{E^\beta}$ and from Eqs. (A26a) and (A28),

$$K^\alpha{}_\beta = L^\alpha{}_\beta + 2E^\alpha{}_\gamma\lambda^{0\gamma}{}_\beta + O(\varepsilon) \quad \text{or} \quad \mathsf{K} = \mathsf{L} + 2\mathsf{E}\mathsf{\lambda}^0 + O(\varepsilon). \tag{A29}$$

These scalings and definitions are consistent with the scalings (30) and the definitions (31) and (32) of the shell and curvature strains for the axisymmetric deformations analysed in Sec. II. Indeed, for these axisymmetric deformations,

$$\mathsf{g} = \begin{pmatrix} 1 & 0 \\ 0 & r^2 \end{pmatrix}, \quad \tilde{\mathsf{g}} = \begin{pmatrix} \tilde{f}_s^2 & 0 \\ 0 & r^2\tilde{f}_\phi^2 \end{pmatrix}, \quad \mathsf{g}^0 = \begin{pmatrix} (f_s^0)^2 & 0 \\ 0 & r^2(f_\phi^0)^2 \end{pmatrix}, \tag{A30}$$

from Eqs. (8), (19b), and (20b). In particular, $\sqrt{g^0/g} = f_s^0 f_\phi^0$. Moreover, Eq. (A26b) yields

$$\tilde{f}_s = f_s^0\sqrt{1 + 2\varepsilon E^s{}_s} = f_s^0(1 + \varepsilon E^s{}_s) + O(\varepsilon^2), \tag{A31a}$$

$$\tilde{f}_\phi = f_\phi^0\sqrt{1 + 2\varepsilon E^\phi{}_\phi} = f_s^0(1 + \varepsilon E^\phi{}_\phi) + O(\varepsilon^2), \tag{A31b}$$

while $E^s{}_\phi = E^\phi{}_s = 0$. Thus, identifying

$$E_s = E^s{}_s, \quad E_\phi = E^\phi{}_\phi, \tag{A31c}$$

we conclude that Eqs. (31) are consistent with Eq. (A26b) at leading order, i.e. at the order to which the shell theory will be valid.

Direct computation relates the components of $\tilde{\lambda}$ to the principal curvatures of $\tilde{\mathcal{S}}$ defined in Eqs. (16), viz.

$$\tilde{\lambda}^s{}_s = \frac{\tilde{\kappa}_s}{\varepsilon f_s^0 f_\phi^0}, \quad \tilde{\lambda}^\phi{}_\phi = \frac{\tilde{\kappa}_\phi}{\varepsilon f_s^0 f_\phi^0}, \tag{A32a}$$

while $\tilde{\lambda}^s{}_\phi = \tilde{\lambda}^\phi{}_s = 0$. Hence Eqs. (30) and (32) are consistent with Eqs. (A23) and (A28) if we identify

$$\lambda_s^0 = \lambda^{0s}{}_s, \quad \varepsilon\lambda_\phi^0 = \lambda^{0\phi}{}_\phi, \quad L_s = L^s{}_s, \quad L_\phi = L^\phi{}_\phi, \tag{A32b}$$

with the off-diagonal components vanishing. However, comparing Eqs. (61) and (A29) shows that the alternative curvature strains defined here are different from those defined in Eqs. (62):

$$K^s{}_s = L_s + 2E_s\lambda_s^0 + O(\varepsilon) \neq L_s + E_s\lambda_s^0 + O(\varepsilon) = K_s, \tag{A32c}$$

using Eq. (59). We are not aware of a tensorial representation of the alternative curvature strains introduced in Eqs. (62) and that vanish for pure stretching deformations.

As in the axisymmetric calculations in Sec. II, it will turn out to be convenient to scale the displacements parallel and perpendicular to the midsurfaces by absorbing the intrinsic stretching of the midsurface. We therefore introduce scaled variables

$$Z^0 = \sqrt{\frac{g^0}{g}}\zeta^0, \quad Z = \sqrt{\frac{g^0}{g}}\tilde{\zeta}, \quad \mathsf{S} = \sqrt{\frac{g^0}{g}}\tilde{\varsigma}. \tag{A33}$$

#### b. Boundary and incompressibility conditions

As in Sec. II, we solve the Cauchy equation (29b) subject to the incompressibility condition $\det \mathbf{F} = 1$ and subject to force-free boundary conditions.

Again as in Sec. II, these boundary conditions on the shell surfaces read $\mathbf{Q}^\pm \boldsymbol{n}^\pm = \mathbf{0}$, where $\mathbf{Q}^\pm$ are evaluated on the surfaces $\zeta = \pm h^\pm$ of $\mathcal{V}$. The normal vectors $\boldsymbol{n}^\pm$ to these undeformed shell surfaces are given by Eqs. (A7), which yield the expansion

$$\boldsymbol{n}^\pm = \boldsymbol{n} \mp \varepsilon\frac{h^\pm{}_{,\alpha}}{g}\boldsymbol{e}^\alpha + O(\varepsilon^2). \tag{A34}$$

The deformation gradient is given in Eq. (A22) with respect to the mixed basis $\tilde{\mathcal{B}} \otimes (\mathcal{B}^0)^*$. In what follows, we shall therefore use Proposition 1 to evaluate the tensorial incompressibility condition $\det \mathbf{F} = 1$.

#### c. Intrinsic volume conservation

We now impose volume conservation of the intrinsic configuration of the shell compared to the undeformed configuration. We need one preliminary result:

*Lemma 1.* Let $\mathsf{M}$ be a $2\times2$ matrix, and $x$ be a scalar. Then

$$\det(\mathsf{I} + x\mathsf{M}) = 1 + x \operatorname{tr} \mathsf{M} + x^2 \det \mathsf{M}.$$

*Proof.* By direct computation,

$$\det\begin{pmatrix} 1 + xM_{11} & xM_{12} \\ xM_{21} & 1 + xM_{22} \end{pmatrix} = 1 + x(M_{11} + M_{22}) + x^2(M_{11}M_{22} - M_{12}M_{21}),$$

which proves the claim. ■

Volume conservation between the undeformed and intrinsic configurations of the shell requires equality of the volume elements, $\sqrt{\det \mathsf{G}} = \sqrt{\det \mathsf{G}^0}$. Now, from definition (A15), $A^\alpha{}_\beta = \delta^\alpha{}_\beta + O(\varepsilon)$, and so Eq. (A19a) yields

$$\sqrt{\det \mathsf{G}} = \varepsilon\sqrt{g} + O(\varepsilon^2). \tag{A35a}$$

Moreover, from Eqs. (A18) and (A19b) with the scalings introduced above and invoking Lemma 1, we find

$$\sqrt{\det \mathsf{G}^0} = \varepsilon\left(\sqrt{\frac{g}{g^0}}Z^0{}_{,\zeta}\right)\left\{\sqrt{g^0}[1 - 2\mathcal{H}^0 Z^0 + \mathcal{K}^0(Z^0)^2]\right\} + O(\varepsilon^2), \tag{A35b}$$

wherein $\mathcal{H}^0 = \frac{1}{2}\lambda^0{}_\alpha{}^\alpha$ and $\mathcal{K}^0 = \det \lambda^0{}_\alpha{}^\beta$, which we think of as (scaled) intrinsic mean and Gaussian curvatures [51]. Since these are not associated with an embedding of $\mathcal{S}^0$ into three-dimensional Euclidean space, we must establish their properties from first principles, based on the assumed symmetry of the intrinsic metric and the intrinsic curvature tensor. The following results are undoubtedly folklore:

*Proposition 2.* If $\mathsf{M}$ is a symmetric matrix and $\mathsf{N}$ is a positive-definite symmetric matrix, then $\mathsf{MN}$ has real eigenvalues.

*Proof.* Since $\mathsf{N}$ is positive-definite and symmetric, it has a symmetric square root $\mathsf{N}^{1/2}$ [56]. Now

$$\mathsf{MN} = (\mathsf{N}^{1/2})^{-1}(\mathsf{N}^{1/2}\mathsf{M}\mathsf{N}^{1/2})\mathsf{N}^{1/2},$$

so $\mathsf{MN}$ is similar to and hence has the same eigenvalues [56] as $\mathsf{N}^{1/2}\mathsf{M}\mathsf{N}^{1/2}$. Since $\mathsf{M}$ and $\mathsf{N}^{1/2}$ are symmetric, so is $\mathsf{N}^{1/2}\mathsf{M}\mathsf{N}^{1/2}$, which therefore has real eigenvalues [56]. Hence $\mathsf{MN}$ has real eigenvalues, too, as claimed. ■

*Corollary 1.* If $\mathsf{M}$ is a symmetric $2\times2$ matrix and $\mathsf{N}$ is a positive-definite symmetric $2\times2$ matrix, then

$$[\operatorname{tr}(\mathsf{MN})]^2 \geqslant 4\det(\mathsf{MN}).$$

*Proof.* By Proposition 2, the $2\times2$ matrix $\mathsf{MN}$ has real eigenvalues $\mu_1, \mu_2$. Hence

$$[\operatorname{tr}(\mathsf{MN})]^2 - 4\det(\mathsf{MN}) = (\mu_1 + \mu_2)^2 - 4\mu_1\mu_2 = (\mu_1 - \mu_2)^2 \geqslant 0,$$

which completes the proof. ■

Now $\lambda^0{}_\alpha{}^\beta = \lambda^0{}_{\alpha\gamma}g^{0\gamma\beta}$. Since $\kappa^0{}_{\alpha\beta}$ is symmetric, so is its rescaling $\lambda^0{}_{\alpha\beta}$. As $g^0{}_{\alpha\beta}$ is symmetric and positive definite, so is its inverse $g^{0\alpha\beta}$. Hence the conditions of Corollary 1 are satisfied; it implies the inequality $(\mathcal{H}^0)^2 \geqslant \mathcal{K}^0$.

Next, integrating the differential equation for $Z^0(\zeta)$ resulting from Eqs. (A35) and imposing $Z^0 = 0$ at $\zeta = 0$, we find

$$Z^0 - \mathcal{H}^0(Z^0)^2 + \frac{\mathcal{K}^0}{3}(Z^0)^3 = \zeta. \tag{A36}$$

Since Eqs. (A35) neglect $O(\varepsilon^2)$ corrections, this result holds at leading order only.

We recall that, by definition, the shell surfaces are at $\zeta^0 = \pm h^0/2$ in the intrinsic configuration, and at $\zeta = \pm h^\pm$ in the undeformed configuration, so that $h^+ + h^- = h$ is the undeformed thickness of the cell sheet. On defining

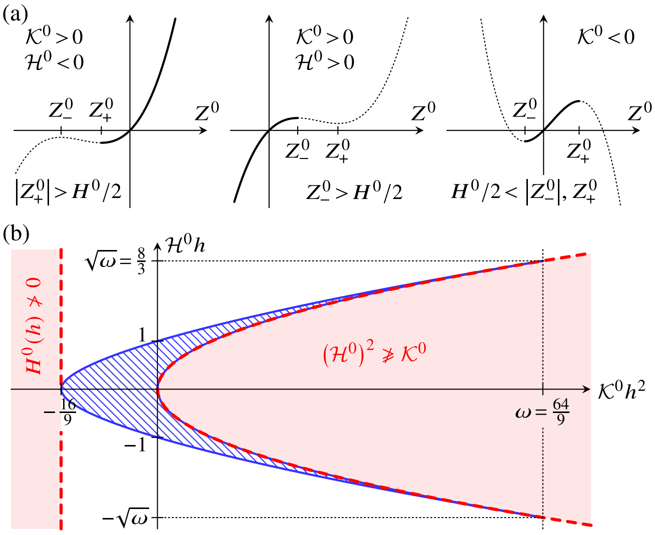


FIG. 6. Intrinsic volume conservation. (a) Plot of $\zeta(Z^0)$ defined in Eq. (A36) for the cases $\mathcal{K}^0>0$, $\mathcal{H}^0<0$; $\mathcal{K}^0>0$, $\mathcal{H}^0>0$; $\mathcal{K}^0<0$. The positions of the turning points at $Z^0=Z^0_\pm$ are indicated, and $\zeta(Z^0)$ must increase monotonically for $|Z^0|<H^0/2$. This condition excludes the dotted parts of the graphs. (b) Intrinsic volume conservation in $(\mathcal{K}^0h^2,\mathcal{H}^0h)$ space: conservation of intrinsic volume is only possible within the region of parameter space enclosed by the solid curve, in which $-16/9<h^2\mathcal{K}^0<\omega=64/9$ and $h|\mathcal{H}^0|<\sqrt{\omega}=8/3$. The dashed lines delimit the regions of parameter space excluded by the inequality $(\mathcal{H}^0)^2\geqslant\mathcal{K}^0$ and the condition that Eq. (A37b) have a positive real solution.

$H^0=h^0\sqrt{g^0/g}$, so that the shell surfaces are at $Z^0=\pm H^0/2$ in the intrinsic configuration, Eq. (A36) yields

$$h^\pm=\frac{H^0}{2}\left[1\mp\frac{\mathcal{H}^0}{2}H^0+\frac{\mathcal{K}^0}{12}(H^0)^2\right],\tag{A37a}$$

whence

$$h=h^++h^-=H^0+\frac{\mathcal{K}^0}{12}(H^0)^3,\tag{A37b}$$

which is a depressed cubic equation for $H^0(h)$ that can be solved in closed form. In particular, Eq. (A37b) has a unique positive real solution if $\mathcal{K}^0>0$, but has no positive real solution if $h^2\mathcal{K}^0<-16/9$. If $0>h^2\mathcal{K}^0>-16/9$, two positive real solutions exist; by continuity, the smaller must be chosen.

More generally, we require that $\zeta$ increase with $Z^0$, for $|Z^0|\leqslant H^0/2$. As $(\mathcal{H}^0)^2\geqslant\mathcal{K}^0$, the cubic in Eq. (A36) has two turning points [Fig. 6(a)], at $Z^0=Z^0_\pm$, where explicit expressions for $Z^0_-\leqslant Z^0_+$ in terms of $\mathcal{K}^0,\mathcal{H}^0$ can be found by solving a quadratic equation. The requirement that $\zeta$ increase with $Z^0$ translates to inequalities $Z^0_\pm\gtrless H^0(h)/2$ depending on the signs of $\mathcal{K}^0,\mathcal{H}^0$ [Fig. 6(a)]. These inequalities involving $h,\mathcal{H}^0,\mathcal{K}^0$ only depend on $\mathcal{H}^0h$ and $\mathcal{K}^0h^2$, since the curvatures can be nondimensionalized with $h$. The inequalities can then be solved numerically to determine the region in $(\mathcal{K}^0h^2,\mathcal{H}^0h)$ parameter space for which intrinsic volume conservation is possible [Fig. 6(b)]. In particular, Fig. 6(b) shows that intrinsic volume conservation requires $-16/9\leqslant\mathcal{K}^0h^2\leqslant\omega$ and $|\mathcal{H}^0h|\leqslant\sqrt{\omega}$, where $\omega$ is a numerical constant. An expression for the boundary of this region can also be determined in closed form using MATHEMATICA (Wolfram, Inc.); this can be used to show that $\omega=64/9$.

For the axisymmetric deformations considered in Sec. II, $\mathcal{K}^0=\lambda^{0s}{}_s\lambda^{0\phi}{}_\phi=O(\varepsilon)$ from Eqs. (A32b). For $\mathcal{K}^0=0$, the condition derived here is $|h\mathcal{H}^0|\leqslant 1$. But, using Eqs. (A32b) again, $h\mathcal{H}^0=h\lambda^0_s/2+O(\varepsilon)=\eta+O(\varepsilon)$ on recalling definition (59), and so this condition is equivalent, as expected, to the condition $|\eta|\leqslant 1$ found in Sec. II.

#### *d. Expansion of the boundary and incompressibility conditions*

To avoid drowning in a bath of indices, we shall use the block matrix notation for tensors [52] introduced above in the expansions that follow below. This means, however, that some care needs to be taken over distinguishing between tensor and matrix transposes and, in particular, over the bases with respect to which transposes of block matrices represent tensor transposes [52]. We shall use the following results repeatedly:

*Proposition 3.* Let $\mathcal{B}$ and $\mathcal{B}'$ be bases of three-dimensional space with corresponding metrics $\mathsf{g},\mathsf{G}$. A tensor $\mathbf{M}$ is represented by the matrix $\mathsf{M}$ with respect to $\mathcal{B}\otimes(\mathcal{B}')^*$. Then $\mathbf{M}^\top$ is represented by $\mathsf{G}^{-1}\mathsf{M}^\top\mathsf{g}$ with respect to $\mathcal{B}'\otimes\mathcal{B}^*$.

*Proof.* Let $\mathcal{B}=\{\boldsymbol{e_\alpha}\}$, $\mathcal{B}'=\{\boldsymbol{E_\alpha}\}$, so that $\mathbf{M}=M^\alpha{}_\beta\boldsymbol{e_\alpha}\otimes\boldsymbol{E^\beta}$. By definition, $\mathbf{M}^\top=M^\beta{}_\alpha\boldsymbol{E^\alpha}\otimes\boldsymbol{e_\beta}=G^{\alpha\gamma}M^\delta{}_\gamma g_{\delta\beta}\boldsymbol{E_\alpha}\otimes\boldsymbol{e^\beta}$, as claimed. ■

*Corollary 2.* Let $\mathcal{B}=\{\boldsymbol{e_\alpha}\}\cup\{\boldsymbol{n}\}$ and $\mathcal{B}'=\{\boldsymbol{E_\alpha}\}\cup\{\boldsymbol{N}\}$ be bases of three-dimensional space, where $\boldsymbol{n},\boldsymbol{N}$ are the respective unit normals to the planes spanned by $\{\boldsymbol{e_\alpha}\}$, $\{\boldsymbol{E_\alpha}\}$. Let the metrics $\mathsf{g},\mathsf{G}$ have components $g_{\alpha\beta}=\boldsymbol{e_\alpha}\cdot\boldsymbol{e_\beta}$, $G_{\alpha\beta}=\boldsymbol{E_\alpha}\cdot\boldsymbol{E_\beta}$. If $\mathbf{M}$ is a tensor such that

$$\mathbf{M}=\left(\begin{array}{c|c}\mathsf{A}&\mathsf{b}\\\hline\mathsf{c}^\top&d\end{array}\right)\quad[\mathcal{B}\otimes(\mathcal{B}')^*],$$

then

$$\mathbf{M}^\top=\left(\begin{array}{c|c}\mathsf{G}^{-1}\mathsf{A}^\top\mathsf{g}&\mathsf{G}^{-1}\mathsf{c}\\\hline\mathsf{b}^\top\mathsf{g}&d\end{array}\right)\quad[\mathcal{B}'\otimes\mathcal{B}^*].$$

*Proof.* Proposition 3 implies that $\mathbf{M}^\top$ is represented, with respect to $\mathcal{B}'\otimes\mathcal{B}^*$, by

$$\left(\begin{array}{c|c}\mathsf{G}&0\\\hline0^\top&1\end{array}\right)^{-1}\left(\begin{array}{c|c}\mathsf{A}&\mathsf{b}\\\hline\mathsf{c}^\top&d\end{array}\right)^\top\left(\begin{array}{c|c}\mathsf{g}&0\\\hline0^\top&1\end{array}\right)=\left(\begin{array}{c|c}\mathsf{G}^{-1}\mathsf{A}^\top\mathsf{g}&\mathsf{G}^{-1}\mathsf{c}\\\hline\mathsf{b}^\top\mathsf{g}&d\end{array}\right),$$

which completes the proof. ■

To expand the boundary and incompressibility conditions, we posit, analogously to Eqs. (37),

$$Z=Z_{(0)}+\varepsilon Z_{(1)}+O(\varepsilon^2),\qquad\mathsf{S}=\mathsf{S}_{(0)}+O(\varepsilon).\tag{A38}$$

*(i) Expansion at order $O(1)$.* On inserting the rescalings (A33) into Eqs. (A16) and (A18), we obtain

$$\mathsf{A}^0=\mathsf{I}-Z^0\lambda^0,\quad\tilde{\mathsf{A}}=\tilde{\mathsf{A}}_{(0)}+O(\varepsilon),\quad\text{with }\tilde{\mathsf{A}}_{(0)}=\mathsf{I}-Z_{(0)}\lambda^0,\tag{A39a}$$

and

$$\mathsf{b}^0=O(\varepsilon),\quad\tilde{\mathsf{b}}=\lambda^0\mathsf{S}_{(0)}+O(\varepsilon),\tag{A39b}$$

and thence, from Eq. (A22),

$$\mathsf{F}=\left(\begin{array}{c|c}\mathsf{B} & \mathsf{v}\\ \hline \mathsf{w}^\top & c\end{array}\right)+O(\varepsilon),\tag{A40}$$

where, with dashes now denoting differentiation with respect to $Z^0$,

$$\mathsf{B}=\tilde{\mathsf{A}}_{(0)}(\mathsf{A}^0)^{-1},\ \ \mathsf{v}=\mathsf{S}'_{(0)},\ \ \mathsf{w}=(\mathsf{A}^0)^{-\top}\mathsf{g}^0\lambda^0\mathsf{S}_{(0)},\ \ c=Z'_{(0)},\tag{A41}$$

since $\tilde{\mathsf{g}}=\mathsf{g}^0+O(\varepsilon)$ from Eq. (A26b). Recalling the definitions $\tilde{g}=\det\tilde{\mathsf{g}}$, $g^0=\det\mathsf{g}^0$ introduced earlier, this also implies $\tilde{g}/g^0=1+O(\varepsilon)$. Using Proposition 1 and on computing the determinant of the block matrix [53] in Eq. (A40), the incompressibility condition thus becomes

$$1=\det\mathsf{F}=(\det\mathsf{B})\big(c-\mathsf{w}^\top\mathsf{B}^{-1}\mathsf{v}\big)+O(\varepsilon).\tag{A42}$$

Next, on substituting the first of Eqs. (A39b) into Eq. (A21) and using Corollary 2,

$$(\mathbf{F}^0)^{-\top}=\left(\begin{array}{c|c}O(1) & O(\varepsilon)\\ \hline O(1) & \left(\zeta^0{}_{,\zeta}\right)^{-1}\end{array}\right).\tag{A43}$$

Moreover, Eqs. (A15) yield $\mathsf{H}=\mathsf{I}+O(\varepsilon)$ using Eqs. (A3), so, on substituting Eqs. (A39) into Eq. (A14b), and using definitions (A41),

$$\tilde{\mathbf{F}}=\left(\begin{array}{c|c}\mathsf{B}\mathsf{A}^0 & \zeta^0{}_{,\zeta}\mathsf{v}\\ \hline \mathsf{w}^\top\mathsf{A}^0 & \zeta^0{}_{,\zeta}c\end{array}\right)+O(\varepsilon).\tag{A44a}$$

Hence, using further properties of block matrices [53] and, again, $\tilde{\mathsf{g}}=\mathsf{g}^0+O(\varepsilon)$ and Corollary 2,

$$\tilde{\mathbf{F}}^{-\top}=\left(\begin{array}{c|c}O(1) & -\left(\zeta^0{}_{,\zeta}\right)^{-1}(\mathsf{g}^0)^{-1}\mathsf{B}^{-\top}\mathsf{w}(c-\mathsf{w}^\top\mathsf{B}^{-1}\mathsf{v})^{-1}\\ \hline O(1) & \left(\zeta^0{}_{,\zeta}\right)^{-1}(c-\mathsf{w}^\top\mathsf{B}^{-1}\mathsf{v})^{-1}\end{array}\right)+O(\varepsilon).\tag{A44b}$$

We now write, as we have done previously in Eqs. (38),

$$\mathbf{Q}=\mathbf{Q}_{(0)}+\varepsilon\mathbf{Q}_{(1)}+O(\varepsilon^2),\qquad p=p_{(0)}+O(\varepsilon).\tag{A45}$$

Inserting Eqs. (A40), (A43), and (A44b) into definition (28), we obtain

$$\mathbf{Q}_{(0)}\boldsymbol{n}=\left(\zeta^0{}_{,\zeta}\right)^{-1}\left(\begin{array}{c}\mathsf{v}+p_{(0)}(\mathsf{g}^0)^{-1}\mathsf{B}^{-\top}\mathsf{w}(c-\mathsf{w}^\top\mathsf{B}^{-1}\mathsf{v})^{-1}\\ \hline c-p_{(0)}(c-\mathsf{w}^\top\mathsf{B}^{-1}\mathsf{v})^{-1}\end{array}\right).\tag{A46}$$

Now, as in Sec. II, the governing equation (29b) of three-dimensional elasticity is, at leading order, $(\mathbf{Q}_{(0)}\boldsymbol{n})_{,\zeta}=\mathbf{0}$, and hence $\mathbf{Q}_{(0)}\boldsymbol{n}$ is independent of $\zeta$. The boundary conditions therefore become $\mathbf{0}=\mathbf{Q}^\pm\boldsymbol{n}^\pm=\mathbf{Q}_{(0)}\boldsymbol{n}+O(\varepsilon)$, where we have used Eq. (A34). It follows that $\mathbf{Q}_{(0)}\boldsymbol{n}\equiv\mathbf{0}$ as in Sec. II.

From Eqs. (A41), $\mathsf{w}^\top\mathsf{B}^{-1}=\mathsf{S}_{(0)}^\top\mathsf{D}$ with $\mathsf{D}=(\lambda^0)^\top\mathsf{g}^0\tilde{\mathsf{A}}_{(0)}^{-1}$, so $\mathsf{B}^{-\top}\mathsf{w}=\mathsf{D}^\top\mathsf{S}_{(0)}$. Equations (A42) and (A46) then yield the leading-order incompressibility and boundary conditions,

$$Z'_{(0)}-\mathsf{S}_{(0)}^\top\mathsf{D}\mathsf{S}'_{(0)}=(\det\mathsf{B})^{-1},\tag{A47a}$$

and hence

$$\mathsf{S}'_{(0)}+p_{(0)}(\det\mathsf{B})(\mathsf{g}^0)^{-1}\mathsf{D}^\top\mathsf{S}_{(0)}=0,\quad Z'_{(0)}-p_{(0)}(\det\mathsf{B})=0.\tag{A47b}$$

In particular, noting that $\mathsf{S}'^\top_{(0)}\mathsf{D}^\top\mathsf{S}_{(0)}=\mathsf{S}^\top_{(0)}\mathsf{D}\mathsf{S}'_{(0)}$ since this expression is a scalar,

$$\begin{aligned}\mathsf{S}'^\top_{(0)}\mathsf{g}^0\mathsf{S}'_{(0)}&=-p_{(0)}(\det\mathsf{B})\mathsf{S}'^\top_{(0)}\mathsf{D}^\top\mathsf{S}_{(0)}\\&=-p_{(0)}(\det\mathsf{B})\mathsf{S}^\top_{(0)}\mathsf{D}\mathsf{S}'_{(0)}\\&=-p_{(0)}(\det\mathsf{B})[Z'_{(0)}-(\det\mathsf{B})^{-1}]=p_{(0)}-(Z'_{(0)})^2.\end{aligned}\tag{A48}$$

Moreover, from Eqs. (A39) and definitions (A41) and using Lemma 1, we obtain

$$\det\mathsf{B}=\frac{\det\tilde{\mathsf{A}}_{(0)}}{\det\mathsf{A}^0}=\frac{1-2\mathcal{H}^0Z_{(0)}+\mathcal{K}^0(Z_{(0)})^2}{1-2\mathcal{H}^0Z^0+\mathcal{K}^0(Z^0)^2}.\tag{A49}$$

Substituting in the second of Eqs. (A47b) and integrating,

$$\tanh^{-1}\frac{\mathcal{K}^0Z_{(0)}-\mathcal{H}^0}{\sqrt{(\mathcal{H}^0)^2-\mathcal{K}^0}}=p_{(0)}\tanh^{-1}\frac{\mathcal{K}^0Z^0-\mathcal{H}^0}{\sqrt{(\mathcal{H}^0)^2-\mathcal{K}^0}}+t,\tag{A50}$$

in which $t$ is a constant of integration; the singular cases $\mathcal{K}^0=0$, $\mathcal{K}^0=\mathcal{H}^0=0$, or $\mathcal{K}^0=(\mathcal{H}^0)^2$ can be dealt with similarly, but we will not discuss these in detail.

Next, by definition, on the midsurface $Z^0=0$, we have $Z_{(0)}=0$ and $\mathsf{S}_{(0)}=0$. Thus $\det\mathsf{B}=1$ on $Z^0=0$, and hence, successively from Eqs. (A47), $Z'_{(0)}$=0, $\mathsf{S}'_{(0)}$=0 on $Z^0$=0, and hence $p_{(0)}=1$ (which is constant). Then taking $Z^0=Z_{(0)}=0$ in Eq. (A50) gives $t=0$; the same equation then immediately yields $Z_{(0)}\equiv Z^0$. Finally, Eq. (A48) yields $\mathsf{S}'^\top_{(0)}\mathsf{g}^0\mathsf{S}'_{(0)}=0$, so $\mathsf{S}'_{(0)}\equiv\mathsf{0}$ since $\mathsf{g}^0$ is positive definite. Now $\mathsf{S}_{(0)}=\mathsf{0}$ on $Z^0$=0, so this implies that $\mathsf{S}_{(0)}\equiv\mathsf{0}$, which proves the Kirchhoff "hypothesis" [15] for general large bending deformations.

For axisymmetric deformations, this argument provides an alternative to the direct integration of the leading-order equations in Sec. II.

*(ii) Expansion at order $O(\varepsilon)$.* We now expand further. In particular, extending Eqs. (A39a), we find

$$\tilde{\mathsf{A}}=\mathsf{A}^0-\varepsilon(Z^0\mathsf{L}+Z_{(1)}\lambda^0)+O(\varepsilon^2).\tag{A51}$$

The leading-order solution also shows that $\mathsf{b}^0$, $\tilde{\mathsf{b}}$, $\tilde{\varsigma}$ are all at the most of order $O(\varepsilon)$, whence

$$\mathbf{F}=\mathsf{I}+\varepsilon\left(\begin{array}{c|c}-(Z^0\mathsf{L}+Z_{(1)}\lambda^0)(\mathsf{A}^0)^{-1} & O(1)\\ \hline O(1) & Z'_{(1)}\end{array}\right)+O(\varepsilon^2),\tag{A52}$$

from Eq. (A22). Using Lemma 1 and Eq. (A26b), we also find

$$\begin{aligned}\sqrt{\frac{\tilde{g}}{g^0}}&=(1+2\varepsilon\operatorname{tr}\mathsf{E}+4\varepsilon^2\det\mathsf{E})^{1/2}\\&=1+\varepsilon\operatorname{tr}\mathsf{E}+\frac{\varepsilon^2}{2}[4\det\mathsf{E}-(\operatorname{tr}\mathsf{E})^2]+O(\varepsilon^3).\end{aligned}\tag{A53}$$

Accordingly, from Proposition 1 and using Lemma 1 again,

$$\det\mathbf{F}=1+\varepsilon\{Z'_{(1)}+\operatorname{tr}\mathsf{E}-\operatorname{tr}[(Z^0\mathsf{L}+Z_{(1)}\lambda^0)(\mathsf{A}^0)^{-1}]\}+O(\varepsilon^2).\tag{A54}$$

The incompressibility condition $\det\mathbf{F}=1$ thus yields, at order $O(\varepsilon)$, an ordinary differential equation for $Z_{(1)}$. To make further progress, we shall need the following result:

*Lemma 2.* Let $\mathsf{M}$ be a $2\times2$ matrix, and $x$ be a scalar. Then

$$(\mathsf{I}+x\mathsf{M})^{-1} = \frac{\mathsf{I}+x\,\mathrm{adj}\,\mathsf{M}}{1+x\,\mathrm{tr}\,\mathsf{M}+x^2\det\mathsf{M}}.$$

*Proof.* By definition of the adjugate matrix,

$$(\mathsf{I}+x\mathsf{M})^{-1} = \frac{\mathrm{adj}\,(\mathsf{I}+x\mathsf{M})}{\det(\mathsf{I}+x\mathsf{M})} = \frac{\mathrm{adj}\,(\mathsf{I}+x\mathsf{M})}{1+x\,\mathrm{tr}\,\mathsf{M}+x^2\det\mathsf{M}},$$

using Lemma 1. But, by direct computation,

$$\mathrm{adj}\,(\mathsf{I}+x\mathsf{M}) = \begin{pmatrix}1+xM_{22} & -M_{12}\\ -M_{21} & 1+xM_{11}\end{pmatrix} = \begin{pmatrix}1 & 0\\ 0 & 1\end{pmatrix}+x\begin{pmatrix}M_{22} & -M_{12}\\ -M_{21} & M_{11}\end{pmatrix} = \mathsf{I}+x\,\mathrm{adj}\,\mathsf{M}.$$

The result follows. ■

On multiplying this result by a general $2\times2$ matrix $\mathsf{N}$ and taking the trace on both sides, we obtain

*Corollary 3.* Let $\mathsf{M},\mathsf{N}$ be $2\times2$ matrices, and let $x$ be a scalar. The following equality holds:

$$\mathrm{tr}\,[\mathsf{N}(\mathsf{I}+x\mathsf{M})^{-1}] = \frac{\mathrm{tr}\,\mathsf{N}+x\,\mathrm{tr}\,(\mathsf{N}\,\mathrm{adj}\,\mathsf{M})}{1+x\,\mathrm{tr}\,\mathsf{M}+x^2\det\mathsf{M}}.$$

We shall also need the following observation:

*Lemma 3.* Let $\mathsf{M},\mathsf{N}$ be $2\times2$ matrices. Then

$$\mathrm{tr}\,(\mathsf{N}\,\mathrm{adj}\,\mathsf{M})=\mathrm{tr}\,\mathsf{M}\,\mathrm{tr}\,\mathsf{N}-\mathrm{tr}\,(\mathsf{M}\mathsf{N}) \text{ and } \mathrm{tr}\,(\mathsf{M}\,\mathrm{adj}\,\mathsf{M})=2\det\mathsf{M}.$$

*Proof.* Notice that $\mathsf{M}+\mathrm{adj}\,\mathsf{M}=(\mathrm{tr}\,\mathsf{M})\mathsf{I}$ since

$$\begin{pmatrix}M_{11} & M_{12}\\ M_{21} & M_{22}\end{pmatrix}+\begin{pmatrix}M_{22} & -M_{12}\\ -M_{21} & M_{11}\end{pmatrix} = (M_{11}+M_{22})\begin{pmatrix}1 & 0\\ 0 & 1\end{pmatrix}.$$

Hence $\mathsf{N}\mathsf{M}+\mathsf{N}\,\mathrm{adj}\,\mathsf{M}=(\mathrm{tr}\,\mathsf{M})\mathsf{N}$ on multiplication by $\mathsf{N}$. Taking the trace gives the first result. The second result follows from the definition of the adjugate, $\mathsf{M}\,\mathrm{adj}\,\mathsf{M}=(\det\mathsf{M})\mathsf{I}$, by taking the trace and noting that $\mathrm{tr}\,\mathsf{I}=2$. ■

Combining Corollary 3 and Lemma 3, and recalling the definitions $\mathrm{tr}\,\mathsf{\lambda}^0=2\mathcal{H}^0$, $\det\mathsf{\lambda}^0=\mathcal{K}^0$, we find the differential equation for $Z_{(1)}$ resulting from Eq. (A54) to be

$$Z'_{(1)}+\left(\frac{-2\mathcal{H}^0+2\mathcal{K}^0Z^0}{1-2\mathcal{H}^0Z^0+\mathcal{K}^0(Z^0)^2}\right)Z_{(1)}+\mathrm{tr}\,\mathsf{E}-\frac{Z^0\,\mathrm{tr}\,\mathsf{L}-(Z^0)^2[2\mathcal{H}^0\,\mathrm{tr}\,\mathsf{L}-\mathrm{tr}\,(\mathsf{L}\mathsf{\lambda}^0)]}{1-2\mathcal{H}^0Z^0+\mathcal{K}^0(Z^0)^2}=0. \tag{A55}$$

Integrating and imposing $Z_{(1)}=0$ at $Z^0=0$, we obtain

$$Z_{(1)} = -\frac{\left[Z^0-\mathcal{H}^0(Z^0)^2+\frac{1}{3}\mathcal{K}^0(Z^0)^3\right]\mathrm{tr}\,\mathsf{E}-\frac{1}{2}(Z^0)^2\,\mathrm{tr}\,\mathsf{L}+\frac{1}{3}(Z^0)^3[2\mathcal{H}^0\,\mathrm{tr}\,\mathsf{L}-\mathrm{tr}\,(\mathsf{L}\mathsf{\lambda}^0)]}{1-2\mathcal{H}^0Z^0+\mathcal{K}^0(Z^0)^2}. \tag{A56}$$

*(iii) Expansion at order $O(\varepsilon^2)$.* From Eq. (A52), we may write

$$\mathbf{F} = \left(\begin{array}{c|c}\mathsf{I}+\varepsilon\mathsf{B}_{(1)}+\varepsilon^2\mathsf{B}_{(2)}+O(\varepsilon^3) & \varepsilon\mathsf{v}_{(1)}+O(\varepsilon^2)\\ \hline \varepsilon\mathsf{w}_{(1)}^{\top}+O(\varepsilon^2) & 1+\varepsilon c_{(1)}+\varepsilon^2c_{(2)}+O(\varepsilon^3)\end{array}\right), \tag{A57}$$

where, in particular and using Lemma 2,

$$\mathsf{B}_{(1)} = -\frac{(Z^0\mathsf{L}+Z_{(1)}\mathsf{\lambda}^0)(\mathsf{I}-Z^0\,\mathrm{adj}\,\mathsf{\lambda}^0)}{1-2\mathcal{H}^0Z^0+\mathcal{K}^0(Z^0)^2}, \tag{A58}$$

in which $Z_{(1)}$ is given by Eq. (A56). Explicit expressions for the terms $\mathsf{B}_{(2)},\mathsf{v}_{(1)},\mathsf{w}_{(1)},c_{(1)},c_{(2)}$ of the formal expansion (A57) could be obtained in terms of the expansions defined in Eqs. (A38), but will turn out not to be required.

From the general expression for the determinant of block matrices [53] and Eq. (A57),

$$\det\mathsf{F} = \left(1+\varepsilon c_{(1)}+\varepsilon^2c_{(2)}\right)\det\left[\mathsf{I}+\varepsilon\mathsf{B}_{(1)}+\varepsilon^2\mathsf{B}_{(2)}-\left(\varepsilon\mathsf{w}_{(1)}^{\top}\right)(\varepsilon\mathsf{v}_{(1)})\right]+O(\varepsilon^3). \tag{A59a}$$

Expanding this using Lemma 1, and using Proposition 1 and Eq. (A53), we deduce that

$$\det\mathbf{F} = 1+\varepsilon(\mathrm{tr}\,\mathsf{B}_{(1)}+\mathrm{tr}\,\mathsf{E}+c_{(1)})+\varepsilon^2\big[\mathrm{tr}\,\mathsf{B}_{(2)}+c_{(2)}+c_{(1)}\,\mathrm{tr}\,\mathsf{B}_{(1)}+\det\mathsf{B}_{(1)}-\mathsf{w}_{(1)}^{\top}\mathsf{v}_{(1)} + (\mathrm{tr}\,\mathsf{B}_{(1)}+c_{(1)})\,\mathrm{tr}\,\mathsf{E}+2\det\mathsf{E}-\tfrac{1}{2}(\mathrm{tr}\,\mathsf{E})^2\big]+O(\varepsilon^3). \tag{A59b}$$

Next we introduce a formal expansion of the intrinsic deformation gradient,

$$\mathbf{F^0} = \left(\begin{array}{c|c}\mathsf{B}^0_{(0)}+O(\varepsilon) & \mathsf{0}\\ \hline \varepsilon\mathsf{w}^{0\top}_{(1)}+O(\varepsilon^2) & c^0_{(0)}+O(\varepsilon)\end{array}\right), \tag{A60a}$$

from Eq. (A17b) and using the first of Eqs. (A39b). In this expansion, $c^0_{(0)}=\zeta^0{}_{,\zeta}$, which is positive by assumption. The values of the expansion terms $\mathsf{B}^0_{(0)}$ and $\mathsf{w}^0_{(1)}$ will turn out to be of no consequence. In particular, using Corollary 2,

$$(\mathbf{F^0})^{-\top} = \left(\begin{array}{c|c}(\mathsf{g}^0)^{-1}\left(\mathsf{B}^0_{(0)}\right)^{-\top}\mathsf{g}+O(\varepsilon) & -\varepsilon\dfrac{(\mathsf{g}^0)^{-1}\left(\mathsf{B}^0_{(0)}\right)^{-\top}\mathsf{w}^0_{(1)}}{c^0_{(0)}}+O(\varepsilon^2)\\ \hline \mathsf{0}^{\top} & \dfrac{1}{c^0_{(0)}}+O(\varepsilon)\end{array}\right). \tag{A60b}$$

Moreover, from Eqs. (A57) and (A60a),

$$\tilde{\mathbf{F}} = \left(\begin{array}{c|c} \mathsf{B}^0_{(0)} + O(\varepsilon) & \varepsilon c^0_{(0)}\mathsf{v}_{(1)} + O(\varepsilon^2) \\ \hline \varepsilon\big(\mathsf{w}^\top_{(1)}\mathsf{B}^0_{(0)} + \mathsf{w}^{0\top}_{(1)}\big) + O(\varepsilon^2) & c^0_{(0)} + O(\varepsilon) \end{array}\right), \tag{A61a}$$

so that, using the general expression for the inverse of a block matrix [53] and, once again, Corollary 2 and $\tilde{\mathsf{g}} = \mathsf{g}^0 + O(\varepsilon)$,

$$\tilde{\mathbf{F}}^{-\top} = \left(\begin{array}{c|c} (\mathsf{g}^0)^{-1}\big(\mathsf{B}^0_{(0)}\big)^{-\top}\mathsf{g} + O(\varepsilon) & -\varepsilon\dfrac{(\mathsf{g}^0)^{-1}}{c^0_{(0)}}\Big[\mathsf{w}_{(1)} + \big(\mathsf{B}^0_{(0)}\big)^{-\top}\mathsf{w}^0_{(1)}\Big] + O(\varepsilon^2) \\ \hline O(\varepsilon) & \dfrac{1}{c^0_{(0)}} + O(\varepsilon) \end{array}\right). \tag{A61b}$$

On substituting Eqs. (A57), (A60b), and (A61b) into definition (28) and recalling that $p = 1 + O(\varepsilon)$, we obtain

$$\mathbf{Q} = \left(\begin{array}{c|c} O(\varepsilon) & \varepsilon\dfrac{\mathsf{v}_{(1)} + (\mathsf{g}^0)^{-1}\mathsf{w}_{(1)}}{c^0_{(0)}} + O(\varepsilon^2) \\ \hline O(\varepsilon) & O(\varepsilon) \end{array}\right), \tag{A62a}$$

and hence

$$\mathbf{Q}_{(0)} = \mathbf{O}, \quad \mathbf{Q}_{(1)}\boldsymbol{n} = \left(\begin{array}{c} \dfrac{\mathsf{v}_{(1)} + (\mathsf{g}^0)^{-1}\mathsf{w}_{(1)}}{c^0_{(0)}} \\ \hline O(1) \end{array}\right). \tag{A62b}$$

As in Sec. II, the fact that $\mathbf{Q}_{(0)} = \mathbf{O}$ implies that, at leading order, Eq. (29b) is $(\mathbf{Q}_{(1)}\boldsymbol{n})_{,\zeta} = \mathbf{0}$, with boundary conditions $\mathbf{Q}^\pm_{(1)}\boldsymbol{n}^\pm = \mathbf{0}$, which, as above, leads to $\mathbf{Q}_{(1)}\boldsymbol{n} \equiv \mathbf{0}$. This and the incompressibility condition $\det \mathbf{F} = 1$ yield, from Eqs. (A59b) and (A62b),

$$c_{(1)} = -\mathrm{tr}\,\mathsf{B}_{(1)} - \mathrm{tr}\,\mathsf{E}, \quad \mathsf{w}_{(1)} = -\mathsf{g}^0\mathsf{v}_{(1)}, \tag{A63a}$$

and hence

$$c_{(2)} = -\mathrm{tr}\,\mathsf{B}_{(2)} + (\mathrm{tr}\,\mathsf{B}_{(1)} + \mathrm{tr}\,\mathsf{E})\,\mathrm{tr}\,\mathsf{B}_{(1)} - \det\mathsf{B}_{(1)} - 2\det\mathsf{E} + \tfrac{3}{2}(\mathrm{tr}\,\mathsf{E})^2 - \mathsf{v}^\top_{(1)}\mathsf{g}^0\mathsf{v}_{(1)}. \tag{A63b}$$

### *e. Asymptotic expansion of the constitutive relations*

To expand the constitutive relations and hence obtain the asymptotic expansion of the three-dimensional energy density, we need one more result:

*Lemma 4.* Let $\mathsf{M}, \mathsf{N}$ be $2\times2$ matrices. Then
(i) $\mathrm{tr}\,(\mathsf{M}^2) = (\mathrm{tr}\,\mathsf{M})^2 - 2\det\mathsf{M}$,
(ii) $\mathrm{tr}\,(\mathsf{M}^2\mathsf{N}) = \mathrm{tr}\,\mathsf{M}\,\mathrm{tr}\,(\mathsf{M}\mathsf{N}) - \det\mathsf{M}\,\mathrm{tr}\,\mathsf{N}$.

*Proof.* The Cayley–Hamilton theorem [53] for a $2\times2$ matrix states that $\mathsf{M}^2 = (\mathrm{tr}\,\mathsf{M})\mathsf{M} - (\det\mathsf{M})\mathsf{I}$. Taking the trace on both sides of this relation and noting that $\mathrm{tr}\,\mathsf{I} = 2$, we obtain (i). Multiplying the Cayley–Hamilton relation by $\mathsf{N}$ and taking the trace yields (ii). ■

We start by computing the expansion of the (left) Cauchy–Green tensor $\mathbf{C} = \mathbf{F}^\top\mathbf{F}$. From Eq. (A57), we obtain

$$\mathbf{F}^\top = \left(\begin{array}{c|c} \mathsf{I} + \varepsilon\big[2\mathsf{E} + (\mathsf{g}^0)^{-1}\mathsf{B}^\top_{(1)}\mathsf{g}^0\big] + \varepsilon^2\big[2(\mathsf{g}^0)^{-1}\mathsf{B}^\top_{(1)}\mathsf{g}^0\mathsf{E} + (\mathsf{g}^0)^{-1}\mathsf{B}^\top_{(2)}\mathsf{g}^0\big] + O(\varepsilon^3) & \varepsilon(\mathsf{g}^0)^{-1}\mathsf{w}_{(1)} + O(\varepsilon^2) \\ \hline \varepsilon\mathsf{v}^\top_{(1)}\mathsf{g}^0 + O(\varepsilon^2) & 1 + \varepsilon c_{(1)} + \varepsilon^2 c_{(2)} + O(\varepsilon^3) \end{array}\right), \tag{A64}$$

using Corollary 2 and Eq. (A26b), and hence

$$\mathbf{C} = \left(\begin{array}{c|c} \begin{array}{l} \mathsf{I} + \varepsilon\big[2\mathsf{E} + \mathsf{B}_{(1)} + (\mathsf{g}^0)^{-1}\mathsf{B}^\top_{(1)}\mathsf{g}^0\big] + \varepsilon^2\big\{2\big[\mathsf{E}\mathsf{B}_{(1)} + (\mathsf{g}^0)^{-1}\mathsf{B}^\top_{(1)}\mathsf{g}^0\mathsf{E}\big] \\ +\mathsf{B}_{(2)} + (\mathsf{g}^0)^{-1}\mathsf{B}^\top_{(2)}\mathsf{g}^0 + (\mathsf{g}^0)^{-1}\mathsf{B}^\top_{(1)}\mathsf{g}^0\mathsf{B}_{(1)} + (\mathsf{g}^0)^{-1}\mathsf{w}_{(1)}\mathsf{w}^\top_{(1)}\big\} + O(\varepsilon^3) \end{array} & O(\varepsilon) \\ \hline O(\varepsilon) & 1 + 2\varepsilon c_{(1)} + \varepsilon^2\big(2c_{(2)} + c^2_{(1)} + \mathsf{v}^\top_{(1)}\mathsf{g}^0\mathsf{v}_{(1)}\big) + O(\varepsilon^3) \end{array}\right). \tag{A65}$$

We recall general properties of the trace operator: for matrices $\mathsf{M}, \mathsf{N}$, $\mathrm{tr}\,\mathsf{M}^\top = \mathrm{tr}\,\mathsf{M}$ and $\mathrm{tr}\,\mathsf{M}\mathsf{N} = \mathrm{tr}\,\mathsf{N}\mathsf{M}$. Since Eq. (A65) represents $\mathbf{C}$ with respect to $\mathcal{B}^0 \otimes (\mathcal{B}^0)^*$, it follows that

$$\begin{aligned} \mathcal{I}_1 &= 3 + \varepsilon[2(\mathrm{tr}\,\mathsf{B}_{(1)} + \mathrm{tr}\,\mathsf{E} + c_{(1)})] + \varepsilon^2\big\{2(\mathrm{tr}\,\mathsf{B}_{(2)} + c_{(2)}) + \big(\mathsf{v}^\top_{(1)}\mathsf{g}^0\mathsf{v}_{(1)} + \mathsf{w}^\top_{(1)}(\mathsf{g}^0)^{-1}\mathsf{w}_{(1)}\big) + c^2_{(1)} + \mathrm{tr}\,\big((\mathsf{g}^0)^{-1}\mathsf{B}^\top_{(1)}\mathsf{g}^0\mathsf{B}_{(1)}\big) \\ &\quad + 2\big[\mathrm{tr}\,(\mathsf{E}\mathsf{B}_{(1)}) + \mathrm{tr}\,\big(\mathsf{E}(\mathsf{g}^0)^{-1}\mathsf{B}^\top_{(1)}\mathsf{g}^0\big)\big]\big\} + O(\varepsilon^3) \\ &= 3 + \varepsilon^2\big\{2(\mathrm{tr}\,\mathsf{E} + \mathrm{tr}\,\mathsf{B}_{(1)})^2 + 2\,\mathrm{tr}\,\mathsf{E}^2 + \mathrm{tr}\,\mathsf{B}^2_{(1)} + \mathrm{tr}\,\big((\mathsf{g}^0)^{-1}\mathsf{B}^\top_{(1)}\mathsf{g}^0\mathsf{B}_{(1)}\big) + 2\,\big[\mathrm{tr}\,(\mathsf{E}\mathsf{B}_{(1)}) + \mathrm{tr}\,\big(\mathsf{E}(\mathsf{g}^0)^{-1}\mathsf{B}^\top_{(1)}\mathsf{g}^0\big)\big]\big\} + O(\varepsilon^3), \end{aligned} \tag{A66a}$$

using Eqs. (A63) and Lemma 4. Recasting this result into a more symmetric form,

$$\mathcal{I}_1 - 3 = 2\varepsilon^2[(\mathrm{tr}\,\hat{\mathsf{E}})^2 + \mathrm{tr}\,\hat{\mathsf{E}}^2] + O(\varepsilon^3), \quad \text{where} \quad \hat{\mathsf{E}} = \mathsf{E} + \tfrac{1}{2}[\mathsf{B}_{(1)} + (\mathsf{g}^0)^{-1}\mathsf{B}_{(1)}^{\top}\mathsf{g}^0], \tag{A66b}$$

so $\hat{\mathsf{E}}$ is the effective two-dimensional strain. Thus Eq. (A66b) determines the leading-order term in the expansion of the three-dimensional energy density $e$ defined in Eqs. (26), analogously to Eq. (55). This completes its asymptotic expansion in the limit of a thin shell that undergoes general large bending deformations.

We are left to express the leading-order expansion of $e$ in terms of tensorial invariants of the midsurface, thereby emphasizing the tensorial nature of the shell theory. We substitute Eq. (A29) into Eq. (A58) to find

$$\mathsf{B}_{(1)} = -\frac{Z^0\mathsf{K} - 2Z^0\mathsf{E}\lambda^0 + Z_{(1)}\lambda^0 - (Z^0)^2\mathsf{K}\,\mathrm{adj}\,\lambda^0 + 2\mathcal{K}^0(Z^0)^2\mathsf{E} - Z_{(1)}Z^0\mathcal{K}^0\mathsf{I}}{1 - 2\mathcal{H}^0Z^0 + \mathcal{K}^0(Z^0)^2} + O(\varepsilon). \tag{A67a}$$

By assumption and definitions (A25) and (A27), tensors $\boldsymbol{\lambda}^0$, $\mathbf{E}$, $\mathbf{K}$ are symmetric. We note that the curvature strain $\mathbf{L}$ is, from its definition in Eq. (A27), not necessarily symmetric. Our choice to switch to a different measure of curvature strain at this stage is therefore motivated by symmetry, and not geometric interpretation as in Sec. II. Now, using Proposition 3, it follows that

$$(\mathsf{g}^0)^{-1}\mathsf{B}_{(1)}^{\top}\mathsf{g}^0 = -\frac{Z^0\mathsf{K} - 2Z^0\lambda^0\mathsf{E} + Z_{(1)}\lambda^0 - (Z^0)^2(\mathrm{adj}\,\lambda^0)\mathsf{K} + 2\mathcal{K}^0(Z^0)^2\mathsf{E} - Z_{(1)}Z^0\mathcal{K}^0\mathsf{I}}{1 - 2\mathcal{H}^0Z^0 + \mathcal{K}^0(Z^0)^2} + O(\varepsilon). \tag{A67b}$$

Moreover, on substituting Eq. (A29) into Eq. (A56), and using Lemma 4, we find

$$Z_{(1)} = -\frac{Z^0\left[1 - \mathcal{H}^0Z^0 - \frac{1}{3}\mathcal{K}^0(Z^0)^2\right]\mathrm{tr}\,\mathsf{E} - \frac{1}{2}(Z^0)^2\left(1 - \frac{4}{3}\mathcal{H}^0Z^0\right)\mathrm{tr}\,\mathsf{K} + (Z^0)^2\,\mathrm{tr}\,\mathsf{E}\lambda^0 - \frac{1}{3}(Z^0)^3\,\mathrm{tr}\,\mathsf{K}\lambda^0}{1 - 2\mathcal{H}^0Z^0 + \mathcal{K}^0(Z^0)^2} + O(\varepsilon). \tag{A68}$$

We introduce the anticommutator $\langle\mathsf{M},\mathsf{N}\rangle$ of two matrices $\mathsf{M},\mathsf{N}$ by setting $\langle\mathsf{M},\mathsf{N}\rangle = (\mathsf{MN} + \mathsf{NM})/2$. With this notation, substituting Eq. (A68) into Eqs. (A67) and the result into the definition of $\hat{\mathsf{E}}$ in Eq. (A66b) yields

$$\begin{aligned}\hat{\mathsf{E}} = {} & \frac{[1 - 2\mathcal{H}^0Z^0 - \mathcal{K}^0(Z^0)^2]\,\mathsf{E} - Z^0\mathsf{K} + 2Z^0\langle\mathsf{E},\lambda^0\rangle + (Z^0)^2\langle\mathsf{K},\mathrm{adj}\,\lambda^0\rangle}{1 - 2\mathcal{H}^0Z^0 + \mathcal{K}^0(Z^0)^2} \\ & + \frac{Z^0\left[1 - \mathcal{H}^0Z^0 - \frac{1}{3}\mathcal{K}^0(Z^0)^2\right]\mathrm{tr}\,\mathsf{E} - \frac{1}{2}(Z^0)^2\left(1 - \frac{4}{3}\mathcal{H}^0Z^0\right)\mathrm{tr}\,\mathsf{K} + (Z^0)^2\,\mathrm{tr}\,\langle\mathsf{E},\lambda^0\rangle - \frac{1}{3}(Z^0)^3\,\mathrm{tr}\,\langle\mathsf{K},\lambda^0\rangle}{[1 - 2\mathcal{H}^0Z^0 + \mathcal{K}^0(Z^0)^2]^2}(\lambda^0 - \mathcal{K}^0Z^0\mathsf{I}) + O(\varepsilon).\end{aligned} \tag{A69a}$$

For the axisymmetric deformations in Sec. II, using the identifications (A31c) and (A32b) of the axisymmetric shell and curvature strains in terms of the components of the general shell and curvature strain tensors used here and Eq. (A29) to switch between curvature strains, we find that $\hat{E}^s{}_s = a_{(1)}$ and $\hat{E}^\phi{}_\phi = b_{(1)}$, where $a_{(1)}, b_{(1)}$ are defined in Eqs. (47). Comparing Eqs. (A66b) and (55) then shows that the general result derived here is consistent with the result for axisymmetric deformations obtained in Sec. II.

The next step in the derivation is to substitute Eq. (A69a), finally, into Eq. (A66b) and hence Eqs. (26). To express the resulting expansion of the energy density $e$ in terms of the first- and second-order invariants that can be constructed from $\boldsymbol{\lambda}^0$, $\mathbf{E}$, $\mathbf{K}$ only, we need to make two more general observations:

*Lemma 5.* Let $\mathsf{U},\mathsf{V},\mathsf{W}$ be 2×2 matrices. Then

$$2\,\mathrm{tr}\,(\langle\mathsf{U},\mathsf{V}\rangle\mathsf{W}) = \mathrm{tr}\,(\langle\mathsf{U},\mathsf{V}\rangle)\,\mathrm{tr}\,\mathsf{W} + \mathrm{tr}\,(\langle\mathsf{V},\mathsf{W}\rangle)\,\mathrm{tr}\,\mathsf{U} + \mathrm{tr}\,(\langle\mathsf{W},\mathsf{U}\rangle)\,\mathrm{tr}\,\mathsf{V} - \mathrm{tr}\,\mathsf{U}\,\mathrm{tr}\,\mathsf{V}\,\mathrm{tr}\,\mathsf{W}.$$

*Proof.* The proof proceeds by direct calculation. We write

$$\mathsf{U} = \begin{pmatrix} U_{11} & U_{12} \\ U_{21} & U_{22}\end{pmatrix}, \quad \mathsf{V} = \begin{pmatrix} V_{11} & V_{12} \\ V_{21} & V_{22}\end{pmatrix}, \quad \mathsf{W} = \begin{pmatrix} W_{11} & W_{12} \\ W_{21} & W_{22}\end{pmatrix}$$

and compute

$$\begin{aligned}2\,\mathrm{tr}\,(\langle\mathsf{U},\mathsf{V}\rangle\mathsf{W}) &= 2U_{11}V_{11}W_{11} + U_{21}V_{12}W_{11} + U_{12}V_{21}W_{11} + U_{21}V_{11}W_{12} + U_{11}V_{21}W_{12} + U_{22}V_{21}W_{12} + U_{21}V_{22}W_{12} + U_{12}V_{11}W_{21} \\ &\quad + U_{11}V_{12}W_{21} + U_{22}V_{12}W_{21} + U_{12}V_{22}W_{21} + U_{21}V_{12}W_{22} + U_{12}V_{21}W_{22} + 2U_{22}V_{22}W_{22} \\ &= (U_{11}V_{11} + U_{21}V_{12} + U_{12}V_{21} + U_{22}V_{22})(W_{11} + W_{22}) + (V_{11}W_{11} + V_{21}W_{12} + V_{12}W_{21} + V_{22}W_{22})(U_{11} + U_{22}) \\ &\quad + (U_{11}W_{11} + U_{21}W_{12} + U_{12}W_{21} + U_{22}W_{22})(V_{11} + V_{22}) - (U_{11} + U_{22})(V_{11} + V_{22})(W_{11} + W_{22}) \\ &= \mathrm{tr}\,(\mathsf{UV})\,\mathrm{tr}\,\mathsf{W} + \mathrm{tr}\,(\mathsf{VW})\,\mathrm{tr}\,\mathsf{U} + \mathrm{tr}\,(\mathsf{UW})\,\mathrm{tr}\,\mathsf{V} - \mathrm{tr}\,\mathsf{U}\,\mathrm{tr}\,\mathsf{V}\,\mathrm{tr}\,\mathsf{W}.\end{aligned}$$

By the symmetry of trace, this completes the proof. ■

*Corollary 4.* Let $\mathsf{U},\mathsf{V},\mathsf{W}$ be 2×2 matrices. Then

$$\mathrm{tr}\,(\langle\mathsf{UV},\mathsf{WV}\rangle) = \mathrm{tr}\,(\langle\mathsf{U},\mathsf{V}\rangle)\,\mathrm{tr}\,(\langle\mathsf{V},\mathsf{W}\rangle) - \det\mathsf{V}\,[\mathrm{tr}\,(\langle\mathsf{U},\mathsf{W}\rangle) - \mathrm{tr}\,\mathsf{U}\,\mathrm{tr}\,\mathsf{W}].$$

*Proof.* Using Lemmata 4 and 5, we find

$$\begin{aligned}\mathrm{tr}\,(\langle\mathsf{UV},\mathsf{WV}\rangle) &= 2\,\mathrm{tr}\,[(\langle\mathsf{U},\mathsf{V}\rangle)\mathsf{WV}] - \mathrm{tr}\,[\mathsf{V}^2(\mathsf{UW})]\\ &= \{\mathrm{tr}\,(\mathsf{UV})\,\mathrm{tr}\,(\mathsf{WV}) + \mathrm{tr}\,[\mathsf{U}(\mathsf{WV})]\,\mathrm{tr}\,\mathsf{V} + \mathrm{tr}\,[\mathsf{V}(\mathsf{WV})]\,\mathrm{tr}\,\mathsf{U} - \mathrm{tr}\,\mathsf{U}\,\mathrm{tr}\,\mathsf{V}\,\mathrm{tr}\,(\mathsf{WV})\} - \{\mathrm{tr}\,\mathsf{V}\,\mathrm{tr}\,[\mathsf{V}(\mathsf{UW})] - \det\mathsf{V}\,\mathrm{tr}\,(\mathsf{UW})\}\\ &= \mathrm{tr}\,(\mathsf{UV})\,\mathrm{tr}\,(\mathsf{WV}) + [\mathrm{tr}\,\mathsf{V}\,\mathrm{tr}\,(\mathsf{VW}) - \det\mathsf{V}\,\mathrm{tr}\,\mathsf{W}]\,\mathrm{tr}\,\mathsf{U} - \mathrm{tr}\,\mathsf{U}\,\mathrm{tr}\,\mathsf{V}\,\mathrm{tr}\,(\mathsf{WV}) + \det\mathsf{V}\,\mathrm{tr}\,(\mathsf{UW})\\ &= \mathrm{tr}\,(\mathsf{UV})\,\mathrm{tr}\,(\mathsf{WV}) + \det\mathsf{V}[\mathrm{tr}\,(\mathsf{UW}) - \mathrm{tr}\,\mathsf{U}\,\mathrm{tr}\,\mathsf{W}],\end{aligned}$$

which, again by the symmetry of trace, finishes the proof. ■

To simplify expressions in subsequent calculations, it will be convenient to rewrite the expression for the effective strain $\hat{\mathsf{E}}$ in Eq. (A69a) as

$$\hat{\mathsf{E}} = e_1\mathsf{E} + e_2\mathsf{K} + e_3\langle\mathsf{E},\lambda^0\rangle + e_4\langle\mathsf{K},\mathrm{adj}\,\lambda^0\rangle + E(\lambda^0 - \mathcal{K}^0Z^0\mathsf{I}) + O(\varepsilon), \tag{A69b}$$

in which $e_1, e_2, e_3, e_4$ are functions of $Z^0$ and $\mathcal{H}^0, \mathcal{K}^0$ only, and $E$ additionally depends on $\mathrm{tr}\,\mathsf{E}$, $\mathrm{tr}\,\mathsf{K}$, $\mathrm{tr}\langle\mathsf{E},\lambda^0\rangle$, $\mathrm{tr}\langle\mathsf{K},\lambda^0\rangle$. Explicit expressions for $e_1, e_2, e_3, e_4$ are easily extracted from Eq. (A69a). It follows that

$$\mathrm{tr}\,\hat{\mathsf{E}} = e_1\,\mathrm{tr}\,\mathsf{E} + e_2\,\mathrm{tr}\,\mathsf{K} + e_3\,\mathrm{tr}\,\langle\mathsf{E},\lambda^0\rangle + e_4\,\mathrm{tr}\,\langle\mathsf{K},\mathrm{adj}\,\lambda^0\rangle + 2E(\mathcal{H}^0 - \mathcal{K}^0Z^0) + O(\varepsilon), \tag{A70a}$$

$$\begin{aligned}\mathrm{tr}\,\hat{\mathsf{E}}^2 &= e_1^2\,\mathrm{tr}\,\mathsf{E}^2 + e_2^2\,\mathrm{tr}\,\mathsf{K}^2 + e_3^2\,\mathrm{tr}\,\langle\mathsf{E},\lambda^0\rangle^2 + e_4^2\,\mathrm{tr}\,\langle\mathsf{K},\mathrm{adj}\,\lambda^0\rangle^2 + E^2[\mathrm{tr}\,(\lambda^0)^2 - 4\mathcal{H}^0\mathcal{K}^0Z^0 + 2(\mathcal{K}^0Z^0)^2] + 2e_1e_2\,\mathrm{tr}\,\langle\mathsf{E},\mathsf{K}\rangle\\ &\quad + 2e_1e_3\,\mathrm{tr}\,\langle\mathsf{E},\langle\mathsf{E},\lambda^0\rangle\rangle + 2e_1e_4\,\mathrm{tr}\,\langle\mathsf{E},\langle\mathsf{K},\mathrm{adj}\,\lambda^0\rangle\rangle + 2e_1E(\mathrm{tr}\,\langle\mathsf{E},\lambda^0\rangle - \mathcal{K}^0Z^0\,\mathrm{tr}\,\mathsf{E}) + 2e_2e_3\,\mathrm{tr}\,\langle\mathsf{K},\langle\mathsf{E},\lambda^0\rangle\rangle\\ &\quad + 2e_2e_4\,\mathrm{tr}\,\langle\mathsf{K},\langle\mathsf{K},\mathrm{adj}\,\lambda^0\rangle\rangle + 2e_2E(\mathrm{tr}\,\langle\mathsf{K},\lambda^0\rangle - \mathcal{K}^0Z^0\,\mathrm{tr}\,\mathsf{K}) + 2e_3e_4\,\mathrm{tr}\,\langle\langle\mathsf{E},\lambda^0\rangle,\langle\mathsf{K},\mathrm{adj}\,\lambda^0\rangle\rangle\\ &\quad + 2e_3E(\mathrm{tr}\,\langle\lambda^0,\langle\mathsf{E},\lambda^0\rangle\rangle - \mathcal{K}^0Z^0\,\mathrm{tr}\,\langle\mathsf{E},\lambda^0\rangle) + 2e_4E(\mathrm{tr}\,\langle\lambda^0,\langle\mathsf{K},\mathrm{adj}\,\lambda^0\rangle\rangle - \mathcal{K}^0Z^0\,\mathrm{tr}\,\langle\mathsf{K},\mathrm{adj}\,\lambda^0\rangle) + O(\varepsilon).\end{aligned} \tag{A70b}$$

Expressing Eqs. (A66b) and hence (26) in terms of first- and second-order invariants only requires simplifying the different traces of higher-order expressions appearing in Eqs. (A70). We do so by applying Lemmata 3, 4, 5, and Corollary 4 repeatedly to find

$$\mathrm{tr}\,\langle\mathsf{K},\mathrm{adj}\,\lambda^0\rangle = 2\mathcal{H}^0\,\mathrm{tr}\,\mathsf{K} - \mathrm{tr}\,\langle\mathsf{K},\lambda^0\rangle,\quad \mathrm{tr}\,(\lambda^0)^2 = 4(\mathcal{H}^0)^2 - 2\mathcal{K}^0, \tag{A71a}$$

$$\mathrm{tr}\,\langle\mathsf{E},\langle\mathsf{E},\lambda^0\rangle\rangle = \mathrm{tr}\,\mathsf{E}\,\mathrm{tr}\,\langle\mathsf{E},\lambda^0\rangle + \mathcal{H}^0[\mathrm{tr}\,\mathsf{E}^2 - (\mathrm{tr}\,\mathsf{E})^2],\quad \mathrm{tr}\,\langle\mathsf{K},\langle\mathsf{K},\mathrm{adj}\,\lambda^0\rangle\rangle = \mathcal{H}^0[\mathrm{tr}\,\mathsf{K}^2 + (\mathrm{tr}\,\mathsf{K})^2] - \mathrm{tr}\,\mathsf{K}\,\mathrm{tr}\,\langle\mathsf{K},\lambda^0\rangle, \tag{A71b}$$

$$\mathrm{tr}\,\langle\lambda^0,\langle\mathsf{E},\lambda^0\rangle\rangle = 2\mathcal{H}^0\mathrm{tr}\,\langle\mathsf{E},\lambda^0\rangle - \mathcal{K}^0\,\mathrm{tr}\,\mathsf{E},\ \mathrm{tr}\,\langle\lambda^0,\langle\mathsf{K},\mathrm{adj}\,\lambda^0\rangle\rangle = \mathcal{K}^0\,\mathrm{tr}\,\mathsf{K}, \tag{A71c}$$

and

$$\mathrm{tr}\,\langle\mathsf{E},\lambda^0\rangle^2 = (\mathcal{H}^0)^2\,\mathrm{tr}\,\mathsf{E}^2 + \mathcal{H}^0\,\mathrm{tr}\,\mathsf{E}\,\mathrm{tr}\,\langle\mathsf{E},\lambda^0\rangle - \left[(\mathcal{H}^0)^2 + \tfrac{1}{2}\mathcal{K}^0\right](\mathrm{tr}\,\mathsf{E})^2 + \tfrac{1}{2}[\mathrm{tr}\,\langle\mathsf{E},\lambda^0\rangle]^2, \tag{A71d}$$

$$\mathrm{tr}\,\langle\mathsf{K},\mathrm{adj}\,\lambda^0\rangle^2 = (\mathcal{H}^0)^2\,\mathrm{tr}\,\mathsf{K}^2 - 3\mathcal{H}^0\,\mathrm{tr}\,\mathsf{K}\,\mathrm{tr}\,\langle\mathsf{K},\lambda^0\rangle + \left[3(\mathcal{H}^0)^2 - \tfrac{1}{2}\mathcal{K}^0\right](\mathrm{tr}\,\mathsf{K})^2 + \tfrac{1}{2}[\mathrm{tr}\,\langle\mathsf{K},\lambda^0\rangle]^2, \tag{A71e}$$

$$\mathrm{tr}\,\langle\mathsf{E},\langle\mathsf{K},\mathrm{adj}\,\lambda^0\rangle\rangle = \mathcal{H}^0(\mathrm{tr}\,\langle\mathsf{E},\mathsf{K}\rangle + \mathrm{tr}\,\mathsf{E}\,\mathrm{tr}\,\mathsf{K}) - \tfrac{1}{2}(\mathrm{tr}\,\langle\mathsf{E},\lambda^0\rangle\,\mathrm{tr}\,\mathsf{K} + \mathrm{tr}\,\langle\mathsf{K},\lambda^0\rangle\,\mathrm{tr}\,\mathsf{E}), \tag{A71f}$$

$$\mathrm{tr}\,\langle\mathsf{K},\langle\mathsf{E},\lambda^0\rangle\rangle = \mathcal{H}^0(\mathrm{tr}\,\langle\mathsf{E},\mathsf{K}\rangle - \mathrm{tr}\,\mathsf{E}\,\mathrm{tr}\,\mathsf{K}) + \tfrac{1}{2}(\mathrm{tr}\,\langle\mathsf{E},\lambda^0\rangle\,\mathrm{tr}\,\mathsf{K} + \mathrm{tr}\,\langle\mathsf{K},\lambda^0\rangle\,\mathrm{tr}\,\mathsf{E}), \tag{A71g}$$

$$\begin{aligned}\mathrm{tr}\,\langle\langle\mathsf{E},\lambda^0\rangle,\langle\mathsf{K},\mathrm{adj}\,\lambda^0\rangle\rangle &= [(\mathcal{H}^0)^2 + \mathcal{K}^0]\,\mathrm{tr}\,\langle\mathsf{E},\mathsf{K}\rangle - \left[(\mathcal{H}^0)^2 + \tfrac{1}{2}\mathcal{K}^0\right]\mathrm{tr}\,\mathsf{E}\,\mathrm{tr}\,\mathsf{K} + \tfrac{1}{2}\mathcal{H}^0(\mathrm{tr}\,\langle\mathsf{E},\lambda^0\rangle\,\mathrm{tr}\,\mathsf{K} + \mathrm{tr}\,\langle\mathsf{K},\lambda^0\rangle\,\mathrm{tr}\,\mathsf{E})\\ &\quad - \tfrac{1}{2}\,\mathrm{tr}\,\langle\mathsf{E},\lambda^0\rangle\,\mathrm{tr}\,\langle\mathsf{K},\lambda^0\rangle.\end{aligned} \tag{A71h}$$

Inserting Eqs. (A71) into Eqs. (A70), and the result into Eqs. (A66b) and (26) as announced, we finally obtain

$$\begin{aligned}e = C\varepsilon^2\{&(\alpha_1\,\mathrm{tr}\,\mathbf{E}^2 + \alpha_2(\mathrm{tr}\,\mathbf{E})^2 + \alpha_3\,\mathrm{tr}\,\mathbf{E}\,\mathrm{tr}\,\langle\mathbf{E},\boldsymbol{\lambda}^0\rangle + \alpha_4[\mathrm{tr}\,\langle\mathbf{E},\boldsymbol{\lambda}^0\rangle]^2) + (\beta_1\,\mathrm{tr}\,\langle\mathbf{E},\mathbf{K}\rangle + \beta_2\,\mathrm{tr}\,\mathbf{E}\,\mathrm{tr}\,\mathbf{K} + \beta_3\,\mathrm{tr}\,\mathbf{E}\,\mathrm{tr}\,\langle\mathbf{K},\boldsymbol{\lambda}^0\rangle\\ &+ \beta_4\,\mathrm{tr}\,\mathbf{K}\,\mathrm{tr}\,\langle\mathbf{E},\boldsymbol{\lambda}^0\rangle + \beta_5\,\mathrm{tr}\,\langle\mathbf{E},\boldsymbol{\lambda}^0\rangle\,\mathrm{tr}\,\langle\mathbf{K},\boldsymbol{\lambda}^0\rangle) + (\gamma_1\,\mathrm{tr}\,\mathbf{K}^2 + \gamma_2(\mathrm{tr}\,\mathbf{K})^2 + \gamma_3\,\mathrm{tr}\,\mathbf{K}\,\mathrm{tr}\,\langle\mathbf{K},\boldsymbol{\lambda}^0\rangle + \gamma_4[\mathrm{tr}\,\langle\mathbf{K},\boldsymbol{\lambda}^0\rangle]^2)\} + O(\varepsilon^3),\end{aligned} \tag{A72}$$

in which the stretching coefficients $\alpha_1, \alpha_2, \alpha_3, \alpha_4$, the coupling coefficients $\beta_1, \beta_2, \beta_3, \beta_4, \beta_5$, and the bending coefficients $\gamma_1, \gamma_2, \gamma_3, \gamma_4$ are rational functions of $Z^0$ and $\mathcal{H}^0, \mathcal{K}^0$, so depend on the intrinsic configuration only. Explicitly,

$$\alpha_1 = \left[\frac{1 - \mathcal{K}^0(Z^0)^2}{1 - 2\mathcal{H}^0Z^0 + \mathcal{K}^0(Z^0)^2}\right]^2, \tag{A73a}$$

$$\gamma_1 = \left[\frac{Z^0(1 - \mathcal{H}^0Z^0)}{1 - 2\mathcal{H}^0Z^0 + \mathcal{K}^0(Z^0)^2}\right]^2. \tag{A73b}$$

The much more complicated explicit expressions for the remaining coefficients in Eq. (A72) are not edifying, and therefore not presented here.

We have been able to use tensor traces rather than matrix traces in this expressions since $\boldsymbol{\lambda}^0, \mathsf{E}, \mathsf{K}$ represent $\boldsymbol{\lambda}^0, \mathbf{E}, \mathbf{K}$ with respect to $\mathcal{B}^0 \otimes (\mathcal{B}^0)^*$. This stresses the tensorial invariance of the theory. The anticommutators in Eq. (A72) could of course be simplified using the symmetry of trace, but we have not done so to emphasize their symmetry.

#### *f. Averaging over the transverse coordinate*

The volume element in the intrinsic configuration $\mathcal{V}^0$ is, by definition and using intrinsic volume conservation and Eq. (A35b),

$$\begin{aligned} dV^0 &= \sqrt{\frac{\det \mathsf{G}^0}{\det \mathsf{G}}}\, dV \\ &= \sqrt{\det \mathsf{G}^0}\left(\frac{dS}{\sqrt{g}}\right) d\zeta \\ &= \varepsilon[1 + 2\mathcal{H}^0 Z^0 + \mathcal{K}^0 (Z^0)^2]\, dS\, dZ^0 + O(\varepsilon^2), \end{aligned} \tag{A74}$$

where $dV$ is the volume element of the undeformed configuration $\mathcal{V}$ and $dS$ is the surface element of the undeformed midsurface $\mathcal{S}$. From Eq. (26), the elastic energy of the shell is therefore

$$\mathcal{E} = \iint_{\mathcal{S}} \hat{e}\, dS, \tag{A75a}$$

in which, at leading order,

$$\hat{e} = \varepsilon \int_{-H^0/2}^{H^0/2} e(Z^0)[1 + 2\mathcal{H}^0 Z^0 + \mathcal{K}^0 (Z^0)^2]\, dZ^0 \tag{A75b}$$

is the effective two-dimensional energy density. In the integral limits, $H^0$ is determined in terms of the undeformed thickness $h$ of the shell by Eq. (A37b).

Since the coefficient functions $\alpha_1, \alpha_2, \alpha_3, \alpha_4, \beta_1, \beta_2, \beta_3, \beta_4, \beta_5$, and $\gamma_1, \gamma_2, \gamma_3, \gamma_4$ that appear in Eq. (A72) are rational functions of $Z^0$, the integral with respect to $Z^0$ in Eq. (A75b) can be performed in closed form. However, even the integrals of the "simple" coefficients given in Eqs. (A73) are extremely cumbersome, so the closed-form expression of Eq. (A75b) is not given here. For this reason, the theory for large bending deformations is likely to be most useful for deformations with some additional symmetry, such as the axisymmetric deformations discussed in Sec. II.

### 3. Limit of small bending deformations

We conclude our calculations by discussing the limit of small bending deformations. In this limit, $\boldsymbol{\lambda}^0 \to \mathbf{O}$, and hence $\mathcal{H}^0, \mathcal{K}^0 \to 0$, and the effective strain in Eq. (A69a) reduces to the rather simpler form

$$\hat{\mathsf{E}} = \mathsf{E} - Z^0 \mathsf{K} + O(\varepsilon), \tag{A76}$$

and so Eqs. (A66b) and (26) yield

$$\begin{aligned} e =& C\varepsilon^2\{[\operatorname{tr} \mathbf{E}^2 + (\operatorname{tr} \mathbf{E})^2] - 2Z^0(\operatorname{tr}\langle \mathbf{E}, \mathbf{K}\rangle + \operatorname{tr} \mathbf{E} \operatorname{tr} \mathbf{K}) \\ &+ (Z^0)^2[\operatorname{tr} \mathbf{K}^2 + (\operatorname{tr} \mathbf{K})^2]\} + O(\varepsilon^3), \end{aligned} \tag{A77}$$

where we have again replaced matrix traces with the corresponding tensor traces. Moreover, Eq. (A37b) shows that, in this limit, $H^0 = h$, and so Eq. (A75b) becomes

$$\begin{aligned} \hat{e} &= \varepsilon \int_{-h/2}^{h/2} e(Z^0)\, dZ^0 \\ &= \varepsilon^3\left\{Ch[\operatorname{tr} \mathbf{E}^2 + (\operatorname{tr} \mathbf{E})^2] + \frac{Ch^3}{12}[\operatorname{tr} \mathbf{K}^2 + (\operatorname{tr} \mathbf{K})^2]\right\} + O(\varepsilon^4), \end{aligned} \tag{A78}$$

which recovers the tensorial form of the incompressible limit of Koiter's shell theory [57].

## APPENDIX B: DERIVATION OF THE GOVERNING EQUATIONS FOR AXISYMMETRIC DEFORMATIONS

In this Appendix, we derive the governing equations for axisymmetric deformations by varying the elastic energy (57a). Similar derivations are given in our previous work [9,10] for the elastic theories considered there, but here, we will keep the explicit asymptotic scalings in the derivation. From Eq. (63) and considering leading-order terms only,

$$\delta\hat{e} = \varepsilon(n_s\, \delta E_s + n_\phi\, \delta E_\phi) + m_s\, \delta K_s + m_\phi\, \delta K_\phi, \tag{B1}$$

wherein the shell stresses and shell moments are

$$n_s = C\varepsilon^2 h[\bar{\alpha}_{ss} E_s + \bar{\alpha}_{s\phi} E_\phi + h(\bar{\beta}_{ss} K_s + \bar{\beta}_{s\phi} K_\phi)], \tag{B2a}$$

$$n_\phi = C\varepsilon^2 h[\bar{\alpha}_{\phi s} E_s + \alpha_{\phi\phi} E_\phi + h(\beta_{\phi s} K_s + \beta_{\phi\phi} K_\phi)], \tag{B2b}$$

$$m_s = C\varepsilon^3 h^2[\bar{\beta}_{ss} E_s + \beta_{\phi s} E_\phi + h(\gamma_{ss} K_s + \gamma_{s\phi} K_\phi)], \tag{B2c}$$

$$m_\phi = C\varepsilon^3 h^2[\bar{\beta}_{s\phi} E_s + \beta_{\phi\phi} E_\phi + h(\gamma_{\phi s} K_s + \gamma_{\phi\phi} K_\phi)], \tag{B2d}$$

since $\bar{\alpha}_{s\phi} = \bar{\alpha}_{\phi s}$, $\gamma_{s\phi} = \gamma_{\phi s}$. Now, from the definitions of the shell and curvature strains in Eqs. (60) and (62),

$$\delta E_s = \frac{\sec\tilde{\psi}\, \delta\tilde{r}' + \tilde{f}_s \tan\tilde{\psi}\, \delta\tilde{\psi}}{\varepsilon f_s^0}, \quad \delta E_\phi = \frac{1}{\varepsilon f_\phi^0}\left(\frac{\delta\tilde{r}}{r}\right), \tag{B3a}$$

and

$$\delta K_s = \frac{\delta\tilde{\psi}'}{(f_s^0)^2 f_\phi^0}, \quad \delta K_\phi = \frac{1}{f_s^0 (f_\phi^0)^2}\left(\frac{\cos\psi}{r}\delta\psi\right). \tag{B3b}$$

Hence, on letting

$$N_s = \frac{n_s}{\tilde{f}_\phi f_s^0}, \qquad N_\phi = \frac{n_\phi}{\tilde{f}_s f_\phi^0}, \tag{B4a}$$

$$M_s = \frac{m_s}{\tilde{f}_\phi (f_s^0)^2 f_\phi^0}, \quad M_\phi = \frac{m_\phi}{\tilde{f}_s f_s^0 (f_\phi^0)^2}, \tag{B4b}$$

we obtain, from Eq. (57a) and using Eqs. (12),

$$\begin{aligned} \frac{\delta\mathcal{E}}{2\pi} &= [\![\tilde{r} N_s \sec\tilde{\psi}\, \delta\tilde{r} + \tilde{r} M_s\, \delta\tilde{\psi}]\!] \\ &- \int_{\mathcal{C}}\left[\left(\frac{d}{ds}(\tilde{r} M_s) - \tilde{r}\tilde{f}_s N_s \tan\tilde{\psi} - \tilde{f}_s M_\phi \cos\tilde{\psi}\right)\delta\tilde{\psi}\right] ds \\ &- \int_{\mathcal{C}}\left[\left(\frac{d}{ds}(\tilde{r} N_s \sec\tilde{\psi}) - \tilde{f}_s N_\phi\right)\delta\tilde{r}\right] ds, \end{aligned} \tag{B5}$$

from which we read off the governing equations and boundary conditions.

As in standard shell theories [26], the apparent singularity in the resulting equations is removed by introducing the transverse shear tension, $T = -N_s \tan\tilde{\psi}$, and we obtain, using Eqs. (13) and (16),

$$\frac{dN_s}{ds} = \tilde{f}_s\left(\frac{N_\phi - N_s}{\tilde{r}}\cos\tilde{\psi} + \tilde{\kappa}_s T\right), \tag{B6a}$$

$$\frac{dM_s}{ds} = \tilde{f}_s\left(\frac{M_\phi - M_s}{\tilde{r}}\cos\tilde{\psi} - T\right). \tag{B6b}$$

Moreover, by differentiating the definition of $T$ and using Eq. (B6a), we find

$$\frac{\mathrm{d}T}{\mathrm{d}s} = -\tilde{f}_s\left(\tilde{\kappa}_s N_s + \tilde{\kappa}_\phi N_\phi + T\frac{\cos\tilde{\psi}}{\tilde{r}}\right). \tag{B6c}$$

Together with the relations

$$\frac{\mathrm{d}\tilde{r}}{\mathrm{d}s} = \tilde{f}_s\cos\tilde{\psi}, \qquad \frac{\mathrm{d}\tilde{\psi}}{\mathrm{d}s} = \tilde{f}_s\tilde{\kappa}_s \tag{B7}$$

from Eqs. (13) and (16), Eqs. (B6) determine the deformed configuration of the shell. Having solved these equations, integrating the otherwise redundant shape equation $\tilde{z}' = \tilde{f}_s \sin\tilde{\psi}$ from Eqs. (13) determines the shape of the shell completely.

### Numerical solution of Eqs. (B6)

We conclude the derivation of the governing equations for axisymmetric deformations with two remarks on the numerical solution of Eqs. (B6).

First, we note that Eqs. (B6) are singular where $\tilde{r} = 0$. At such a point, geometric continuity implies $\tilde{\psi} = 0$. Hence $T = 0$ there by definition, and $N_\phi = N_s$ for regularity in Eq. (B6a). Moreover, by applying l'Hôpital's rule to the definitions in Eqs. (12) and (16), $\tilde{f}_s = \tilde{f}_\phi$, $\tilde{\kappa}_s = \tilde{\kappa}_\phi$. Hence Eqs. (B6) are replaced with

$$\frac{\mathrm{d}N_s}{\mathrm{d}s} = 0, \qquad \frac{\mathrm{d}M_s}{\mathrm{d}s} = 0, \qquad \frac{\mathrm{d}T}{\mathrm{d}s} = -\tilde{f}_s\tilde{\kappa}_s N_s, \tag{B8}$$

of which the first two follow by reflection across the axis of symmetry, and the last follows by applying l'Hôpital's rule to Eq. (B6c) and using the previous observations and Eqs. (B7).

Second, as discussed in Refs. [9,10], too, at each stage of the numerical solution, $\tilde{f}_s, \tilde{f}_\phi, \tilde{\kappa}_s, \tilde{\kappa}_\phi$ must be determined from $\tilde{r}, \tilde{\psi}, M_s, N_s$. To begin with, $\tilde{f}_\phi, \tilde{\kappa}_\phi$ and hence $E_\phi, K_\phi$ are computed directly from $\tilde{r}, \tilde{\psi}$ using their definitions (60) and (62). We can then compute $\tilde{f}_s, \tilde{\kappa}_s$ by noting that, once $\tilde{f}_\phi, E_\phi, K_\phi$ are known, the definitions of $N_s, M_s$ in Eqs. (B2a), (B2c), and (B4) define a system of linear equations for $E_s, K_s$. Its solution and definitions (60) and (62) yield $\tilde{f}_s$ and finally $\tilde{\kappa}_s$. We can then compute $N_\phi, M_\phi$ using Eqs. (B2b), (B2d), and (B4), and thus continue the numerical integration. Moreover, if $\tilde{r} = 0$, we similarly obtain two linear equations for $\tilde{f} = \tilde{f}_s = \tilde{f}_\phi$ and $\tilde{k} = \tilde{f}_s\tilde{\kappa}_s = \tilde{f}_\phi\tilde{\kappa}_\phi$, from the solution of which the numerical integration can be continued.

Varying the energy with respect to geometric variables, as we have done above, obviates the problem of elastic compatibility. This is the question—independent of the problem of incompatibility of the intrinsic configuration [17] that we have discussed when setting up the geometry of the intrinsic configuration—whether a deformation exists that produces a given set of strains and that provides one of the Föppl–von Kármán equations of plate theory [15]. In this context, this discussion of the numerical approach to solving Eqs. (B6) shows explicitly how they give rise to a compatible configuration and therefore how they avoid the problem of elastic compatibility.

## APPENDIX C: NEO-HOOKEAN RELATIONS AS THE THIN SHELL LIMIT OF GENERAL CONSTITUTIVE RELATIONS

In this final Appendix, we show that the effective two-dimensional constitutive relations resulting from Eq. (A66b),

$$e = C\varepsilon^2[(\operatorname{tr}\hat{\mathsf{E}})^2 + \operatorname{tr}\hat{\mathsf{E}}^2] + O(\varepsilon^3), \tag{C1}$$

are general and therefore do not only apply to the incompressible neo-Hookean three-dimensional constitutive relations assumed in Eqs. (26). To prove this, we consider, following Ref. [21], incompressible isotropic energy densities expressible as a general power series

$$e = \frac{1}{2}\sum_{m=0}^{\infty}\sum_{n=0}^{\infty} C_{mn}(\mathcal{I}_1 - 3)^m(\mathcal{I}_2 - 3)^n, \tag{C2}$$

where $\mathcal{I}_1 = \operatorname{tr}\mathbf{C}$ and $\mathcal{I}_2 = (\mathcal{I}_1^2 - \operatorname{tr}\mathbf{C}^2)/2$ are the first two invariants of the Cauchy–Green tensor $\mathbf{C} = \mathbf{F}^\top\mathbf{F}$. We may set $C_{00} = 0$ without loss of generality. The requirement that $e \geqslant 0$ for small, linearly elastic deformations [32] then leads to $C_{10} + C_{01} \geqslant 0$. For $C_{10} + C_{01} = 0$, the material has no linear elastic response (i.e. zero bulk modulus); we do not consider that case, and therefore assume that $C_{10} + C_{01} > 0$.

Using a result of Ref. [21] and the notation of Appendix A, the Cauchy stress tensor for this material is

$$\mathbf{T} = 2[e_{,\mathcal{I}_1}\mathbf{F} + e_{,\mathcal{I}_2}(\mathcal{I}_1\mathbf{F} - \mathbf{FC})]\mathbf{F}^\top - P\mathbf{I}, \tag{C3a}$$

and hence the morphoelastic Piola–Kirchhoff tensor introduced in Eq. (28) is

$$\mathbf{P} = \mathbf{T}\tilde{\mathbf{F}}^{-\top} = 2[e_{,\mathcal{I}_1}\mathbf{F} + e_{,\mathcal{I}_2}(\mathcal{I}_1\mathbf{F} - \mathbf{FC})](\mathbf{F^0})^{-\top} - P\tilde{\mathbf{F}}^{-\top}. \tag{C3b}$$

In Eqs. (C3), $\tilde{\mathbf{F}}$, $\mathbf{F^0}$, and $\mathbf{F} = \tilde{\mathbf{F}}(\mathbf{F^0})^{-1}$ are given by Eqs. (A14b), (A17b), and (A22), respectively, and $P = P_{(0)} + O(\varepsilon)$ is pressure. (We now use an uppercase letter to denote pressure to emphasize that it is scaled differently to Appendix A; in the notation used there, $P = Cp$.)

*(i) Expansion and partial solution at order O(1).* From the leading-order expansion of $\mathbf{F}$ in Eq. (A40) and using Corollary 2 and $\tilde{\mathsf{g}} = \mathsf{g}^0 + O(\varepsilon)$ from Eq. (A26b), we compute

$$\mathbf{F}^\top = \left(\begin{array}{c|c} (\mathsf{g}^0)^{-1}\mathsf{B}^\top\mathsf{g}^0 & (\mathsf{g}^0)^{-1}\mathsf{w} \\ \hline \mathsf{v}^\top\mathsf{g}^0 & c \end{array}\right) + O(\varepsilon), \tag{C4}$$

in which $\mathsf{B}, \mathsf{v}, \mathsf{w}, c$ are given by Eqs. (A41), and thence

$$\mathbf{C} = \left(\begin{array}{c|c} (\mathsf{g}^0)^{-1}\mathsf{B}^\top\mathsf{g}^0\mathsf{B} + (\mathsf{g}^0)^{-1}\mathsf{w}\mathsf{w}^\top & (\mathsf{g}^0)^{-1}\mathsf{B}^\top\mathsf{g}^0\mathsf{v} + c(\mathsf{g}^0)^{-1}\mathsf{w} \\ \hline \mathsf{v}^\top\mathsf{g}^0\mathsf{B} + c\mathsf{w}^\top & \mathsf{v}^\top\mathsf{g}^0\mathsf{v} + c^2 \end{array}\right) + O(\varepsilon), \tag{C5}$$

In particular,

$$\mathcal{I}_1 = \operatorname{tr}\left((\mathsf{g}^0)^{-1}\mathsf{B}^\top\mathsf{g}^0\mathsf{B}\right) + \mathsf{w}^\top(\mathsf{g}^0)^{-1}\mathsf{w} + \mathsf{v}^\top\mathsf{g}^0\mathsf{v} + c^2 + O(\varepsilon). \tag{C6}$$

Since the incompressibility condition is independent of the constitutive relations, its leading-order expansion (A42) still holds true. Using this and the leading-order expansions (A43) and (A44b) and writing $e_{,\mathcal{I}_1} = E_1 + O(\varepsilon)$, $e_{,\mathcal{I}_2} = E_2 + O(\varepsilon)$, Eq. (C3b)

yields

$$\mathsf{P}=\frac{1}{\zeta^0{}_{,\zeta}}\left(\begin{array}{c|c} O(1) & \begin{array}{l}2\{E_1+E_2[\mathrm{tr}\,((\mathsf{g}^0)^{-1}\mathsf{B}^\top\mathsf{g}^0\mathsf{B})+\mathsf{w}^\top(\mathsf{g}^0)^{-1}\mathsf{w}]\}\mathsf{v}\\ \quad-2E_2[\mathsf{B}(\mathsf{g}^0)^{-1}\mathsf{B}^\top\mathsf{g}^0\mathsf{v}+c\mathsf{B}(\mathsf{g}^0)^{-1}\mathsf{w}]+P_{(0)}(\det\mathsf{B})(\mathsf{g}^0)^{-1}\mathsf{B}^{-\top}\mathsf{w}\end{array}\\ \hline O(1) & 2c[E_1+E_2\mathrm{tr}\,((\mathsf{g}^0)^{-1}\mathsf{B}^\top\mathsf{g}^0\mathsf{B})]-2E_2\mathsf{w}^\top(\mathsf{g}^0)^{-1}\mathsf{B}^\top\mathsf{g}^0\mathsf{v}-P_{(0)}\det\mathsf{B}\end{array}\right)+O(\varepsilon). \tag{C7}$$

Writing $\mathbf{P}=\mathbf{P}_{(0)}+\varepsilon\mathbf{P}_{(1)}+O(\varepsilon^2)$, the leading-order boundary condition is $\mathbf{P}_{(0)}\boldsymbol{n}\equiv\mathbf{0}$, similary to Appendix A. Hence, from Eqs. (A42) and (C7), the leading-order problem is

$$c-\mathsf{w}^\top\mathsf{B}^{-1}\mathsf{v}=(\det\mathsf{B})^{-1}, \tag{C8a}$$

$$2\{E_1+E_2[\mathrm{tr}\,((\mathsf{g}^0)^{-1}\mathsf{B}^\top\mathsf{g}^0\mathsf{B})+\mathsf{w}^\top(\mathsf{g}^0)^{-1}\mathsf{w}]\}\mathsf{v}-2E_2(\mathsf{B}(\mathsf{g}^0)^{-1}\mathsf{B}^\top\mathsf{g}^0\mathsf{v}+c\mathsf{B}(\mathsf{g}^0)^{-1}\mathsf{w})+P_{(0)}(\det\mathsf{B})(\mathsf{g}^0)^{-1}\mathsf{B}^{-\top}\mathsf{w}=0, \tag{C8b}$$

$$2c[E_1+E_2\,\mathrm{tr}\,((\mathsf{g}^0)^{-1}\mathsf{B}^\top\mathsf{g}^0\mathsf{B})]-2E_2\mathsf{w}^\top(\mathsf{g}^0)^{-1}\mathsf{B}^\top\mathsf{g}^0\mathsf{v}-P_{(0)}\det\mathsf{B}=0. \tag{C8c}$$

These equations have a trivial solution

$$Z_{(0)}\equiv Z^0,\quad \mathsf{S}_{(0)}\equiv\mathsf{0},\quad P_{(0)}=C_{10}+2C_{01}, \tag{C9}$$

analogous to the leading-order solution found in Appendix A and for which, from Eqs. (A41), $\mathsf{B}=\mathsf{I}$, $\mathsf{v}=\mathsf{w}=\mathsf{0}$, $c=1$, and hence $\mathbf{C}=\mathbf{I}+O(\varepsilon)$, so $\mathcal{I}_1=\mathcal{I}_2=3+O(\varepsilon)$ and thus $E_1=C_{10}/2$, $E_2=C_{01}/2$ from Eq. (C2). We were not, however, able to show that this is the only solution of the nonlinear first-order differential equations for $Z_{(0)}$, $\mathsf{S}_{(0)}$ as functions of $Z^0$ provided by Eqs. (C8) that satisfies the conditions $Z_{(0)}=0$, $\mathsf{S}_{(0)}=\mathsf{0}$ on the midsurface $Z^0=0$. In this respect, our solution of the leading-order problem remains partial.

Our failure to solve Eqs. (C8) emphasizes once again that what distinguishes these problems of large bending deformations from classical problems in elastic shell theories is the fact that the leading-order problem for large bending deformations is not trivial. In fact, were a second solution of Eqs. (C8) to exist, global energy considerations would select the solution; this would open a new can of worms in the analysis.

*(ii) Expansion at order $O(\varepsilon)$.* At this stage, we take Eqs. (C9) as the solution of the leading-order problem (C8) and proceed thence. In particular, the deformation gradient still has an expansion of the form in Eq. (A57). Hence Eq. (A64) still holds true, and we compute

$$\mathbf{C}=\left(\begin{array}{c|c}\mathsf{I}+\varepsilon[2\mathsf{E}+\mathsf{B}_{(1)}+(\mathsf{g}^0)^{-1}\mathsf{B}_{(1)}^\top\mathsf{g}^0] & \varepsilon[\mathsf{v}_{(1)}+(\mathsf{g}^0)^{-1}\mathsf{w}_{(1)}]\\ \hline \varepsilon[\mathsf{v}_{(1)}^\top\mathsf{g}^0+\mathsf{w}_{(1)}^\top] & 1+2\varepsilon c_{(1)}\end{array}\right)+O(\varepsilon^2), \tag{C10a}$$

$$\mathbf{C}^2=\left(\begin{array}{c|c}\mathsf{I}+2\varepsilon[2\mathsf{E}+\mathsf{B}_{(1)}+(\mathsf{g}^0)^{-1}\mathsf{B}_{(1)}^\top\mathsf{g}^0] & O(\varepsilon)\\ \hline O(\varepsilon) & 1+4\varepsilon c_{(1)}\end{array}\right)+O(\varepsilon^2), \tag{C10b}$$

whence

$$\mathcal{I}_1=3+\varepsilon[2(\mathrm{tr}\,\mathsf{E}+\mathrm{tr}\,\mathsf{B}_{(1)}+c_{(1)})]+O(\varepsilon^2), \tag{C11a}$$

$$\mathcal{I}_2=3+\varepsilon[4(\mathrm{tr}\,\mathsf{E}+\mathrm{tr}\,\mathsf{B}_{(1)}+c_{(1)})]+O(\varepsilon^2). \tag{C11b}$$

The incompressibility condition being independent of the constitutive relations, Eq. (A59b) and hence the first of Eqs. (A63a) still hold. The latter implies $\mathcal{I}_1=\mathcal{I}_2=3+O(\varepsilon^2)$. Thus

$$e=\tfrac{1}{2}[C_{10}(\mathcal{I}_1-3)+C_{01}(\mathcal{I}_2-3)]+O(\varepsilon^4), \tag{C12}$$

and, in particular, $e_{,\mathcal{I}_1}=C_{10}/2+O(\varepsilon^2)$, $e_{,\mathcal{I}_2}=C_{01}/2+O(\varepsilon^2)$. In this way, the constitutive relations have reduced, up to smaller corrections, to those of a Mooney–Rivlin solid [17]. Moreover, Eq. (A60a) and hence Eqs. (A60b) and (A61b) still hold. Since $P=C_{10}+2C_{01}+O(\varepsilon)$, it follows that

$$\mathbf{P}=\left(\begin{array}{c|c}O(\varepsilon) & \varepsilon\dfrac{C_{10}+C_{01}}{c^0_{(0)}}[\mathsf{v}_{(1)}+(\mathsf{g}^0)^{-1}\mathsf{w}_{(1)}]+O(\varepsilon^2)\\ \hline O(\varepsilon) & O(\varepsilon)\end{array}\right)\implies \mathbf{P}_{(0)}=\mathbf{O},\ \mathbf{P}_{(1)}\boldsymbol{n}=\left(\begin{array}{c}\dfrac{C_{10}+C_{01}}{c^0_{(0)}}[\mathsf{v}_{(1)}+(\mathsf{g}^0)^{-1}\mathsf{w}_{(1)}]\\ \hline O(1)\end{array}\right). \tag{C13}$$

Similarly to Appendix A, the boundary conditions now imply $\mathbf{P}_{(1)}\boldsymbol{n}\equiv\mathbf{0}$, so, noting that $c^0_{(0)}>0$ and $C_{10}+C_{01}>0$, the second of Eqs. (A63a) also still holds.

*(iii) Expansion at order $O(\varepsilon^2)$.* Since the expansion (A59b) of the incompressibility condition still holds, Eqs. (A63a) still imply Eq. (A63b) and hence Eq. (A66a). Meanwhile, Eqs. (A63a) and (C10a) show that the off-diagonal terms in Eq. (A65) are

in fact of order $O(\varepsilon^2)$, so it follows from Eq. (A65) that

$$\begin{aligned}\mathrm{tr}\,\mathbf{C}^2 =\ &\mathrm{tr}\,\big\{\mathsf{I}+\varepsilon\big(2\mathsf{E}+\mathsf{B}_{(1)}+(\mathsf{g}^0)^{-1}\mathsf{B}_{(1)}^\top\mathsf{g}^0\big)+\varepsilon^2\big[2\big(\mathsf{E}\mathsf{B}_{(1)}+(\mathsf{g}^0)^{-1}\mathsf{B}_{(1)}^\top\mathsf{g}^0\mathsf{E}\big)+\mathsf{B}_{(2)}+(\mathsf{g}^0)^{-1}\mathsf{B}_{(2)}^\top\mathsf{g}^0+(\mathsf{g}^0)^{-1}\mathsf{B}_{(1)}^\top\mathsf{g}^0\mathsf{B}_{(1)}\\
&+(\mathsf{g}^0)^{-1}\mathsf{w}_{(1)}\mathsf{w}_{(1)}^\top\big]+O(\varepsilon^3)\big\}^2+\big[1+2\varepsilon c_{(1)}+\varepsilon^2\big(2c_{(2)}+c_{(1)}^2+\mathsf{v}_{(1)}^\top\mathsf{g}^0\mathsf{v}_{(1)}\big)+O(\varepsilon^3)\big]^2+O(\varepsilon^4)\\
=\ &3+4\varepsilon(c_{(1)}+\mathrm{tr}\,\mathsf{E}+\mathrm{tr}\,\mathsf{B}_{(1)})+2\varepsilon^2\,\big[2\,\mathrm{tr}\,\mathsf{E}^2+4\,\mathrm{tr}\,(\mathsf{E}\mathsf{B}_{(1)})+4\,\mathrm{tr}\,\big(\mathsf{E}(\mathsf{g}^0)^{-1}\mathsf{B}_{(1)}^\top\mathsf{g}^0\big)+\mathrm{tr}\,\mathsf{B}_{(1)}^2+2\,\mathrm{tr}\,\big(\mathsf{B}_{(1)}(\mathsf{g}^0)^{-1}\mathsf{B}_{(1)}^\top\mathsf{g}^0\big)\\
&+2\,\mathrm{tr}\,\mathsf{B}_{(2)}+\mathsf{w}_{(1)}^\top(\mathsf{g}^0)^{-1}\mathsf{w}_{(1)}+3c_{(1)}^2+2c_{(2)}+\mathsf{v}_{(1)}^\top\mathsf{g}^0\mathsf{v}_{(1)}\big]+O(\varepsilon^3)\\
=\ &3+4\varepsilon^2\big\{2(\mathrm{tr}\,\mathsf{E}+\mathrm{tr}\,\mathsf{B}_{(1)})^2+2\,\mathrm{tr}\,\mathsf{E}^2+\mathrm{tr}\,\mathsf{B}_{(1)}^2+\mathrm{tr}\,\big((\mathsf{g}^0)^{-1}\mathsf{B}_{(1)}^\top\mathsf{g}^0\mathsf{B}_{(1)}\big)+2\big[\mathrm{tr}\,(\mathsf{E}\mathsf{B}_{(1)})+\mathrm{tr}\,\big(\mathsf{E}(\mathsf{g}^0)^{-1}\mathsf{B}_{(1)}^\top\mathsf{g}^0\big)\big]\big\}+O(\varepsilon^3),\end{aligned}\tag{C14}$$

using Eqs. (A63) and Lemma 4, similarly to the calculations leading up to Eq. (A66a).

Finally, if we write $\mathcal{I}_1=3+\varepsilon^2 I_{(2)}+O(\varepsilon^3)$ using Eq. (A66a), then Eq. (C14) shows that $\mathrm{tr}\,\mathbf{C}^2=3+4\varepsilon^2 I_{(2)}+O(\varepsilon^3)$. These expansions imply that $\mathcal{I}_2=3+\varepsilon^2 I_{(2)}+O(\varepsilon^3)$. Equivalently, $\mathcal{I}_2-3=\mathcal{I}_1-3+O(\varepsilon^3)$. Hence, from Eq. (C12),

$$e=\frac{C}{2}(\mathcal{I}_1-3)+O(\varepsilon^3),\quad\text{with}\quad C=C_{10}+C_{01}>0.\tag{C15}$$

Up to smaller corrections, these are the neo-Hookean constitutive relations assumed in Eqs. (26) and throughout Sec. II and Appendix A, and which, as shown there, indeed reduce at order $O(\varepsilon^2)$ to the effective two-dimensional constitutive relations in Eq. (C1). Assuming that the trivial solution (C9) of the leading-order problem defined by Eqs. (C8) is unique, this proves our claim in Sec. IV, that these effective two-dimensional constitutive relations are general.
